# Supplementary material for: The pan-PPAR agonist lanifibranor improves cardiometabolic health in patients with metabolic dysfunction-associated steatohepatitis
Source: Nat Commun. 2024 May 10;15:3962. doi: 10.1038/s41467-024-47919-9 (PMC11087475; doi:10.1038/s41467-024-47919-9)
Supplement: Supplementary file 1 — Supplementary Information [file 41467_2024_47919_MOESM1_ESM.pdf]

SUPPLEMENTARY MATERIALS

TITLE: The pan-PPAR agonist lanifibranor improves cardiometabolic health in patients with metabolic dysfunction-associated steatohepatitis

AUTHORS: Michael P. Cooreman, Javed Butler, Robert P. Giugliano, Faiez Zannad, Lucile Dzen, Philippe Huot-Marchand, Martine Baudin, Daniel R. Beard, Jean-Louis Junien, Pierre Broqua, Manal F. Abdelmalek, Sven M. Francque.

Supplementary Tables

Supplementary Table 1. List of Independent Ethics Committee / Institutional Review Board

Country	CEC or LEC for Site	Ethical Committees (or IRB)	Chair Person
Australia	CEC - all Sites	Monash Health Human Research EthicsCommittee (EC00382):<br>Research Support ServicesMonash Health,<br>Level 2, Block 1, Monash Medical Centre 246 Clayton Road, Clayton, Victoria, 3168.<br>Email: research@monashhealth.org	Deborah DELL (Manager)
Australia	Site 1201	Research Support ServicesMonash Health,<br>Level 2, Block 1, Monash Medical Centre 246 Clayton Road, Clayton, Victoria, 3168.<br>Email: michael.kios@monashhealth.org	Deborah DELL (Manager)
Australia	Site 1202	NALHN Research Governance OfficerResearch Secretariat<br>Northern Adelaide Local Health NetworkLyell McEwin Hospital<br>Clinical Trials Unit, Level 2 Haydown Road<br>Elizabeth Vale SA 5112<br>Email: healthnalhnrgo@sa.gov.au	Lorraine CICHON
Australia	Site 1203	Research Services Research GovernanceOfficer<br>Executive Administration Lower GroundFloor<br>Dr. James Mayne Building<br>Royal Brisbane and Women's HospitalCampus<br>Herston Qld 4029 Generic Email: RBWH-RGO@health.qld.gov.au<br>www.health.qld.gov.au/metronorth	Janani Balakrishnan MCOM
Australia	Site 1204	Research Governance Officer<br>Southern Adelaide Local Health Network Flinders Medical Centre, Bedford Park SA5042<br>(WARD 6C Room 6A219)<br>Email: karen.saxty@sa.gov.au http://www.sahealth.sa.gov.au/SALHNresearch	Karen SAXTY BHlthSc (Hons)
Australia	Site 1205	Research Governance OfficerLevel 2, Education Building<br>Fiona Stanley Hospital<br>14 Barry Marsh ParadeMurdoch, WA 6150<br>Email: SMHS.RGO@health.wa.gov.au	Cathy GARTNER
Austria	CEC - Site 1001 (no patient screened in Site 1002)	Ethikkommission der MedizinischenUniversität Wien,<br>Borschkegasse 8b/E06,1090 Wien, Österreich<br>Tel: +43(0)1 404 00-21470;<br>Fax: +43(0)1404 00-16900<br>Email: ethik-kom@meduniwien.ac.at http://ethikkommission meduniwien.ac.at/	Jürgen ZEZULA
Belgium	CEC - Site 0001	Comité voor Medische Ethiek<br>UZA, Wilrijkstraat 10, B-2650 Edegem<br>Tel +32 3 821 30 00<br>Email: EthischComite@uza.bewww.uza.be	Peter MICHIELSEN
Belgium	LEC - Site 0002	Comité d'Ethique hospitalo-facultaire Promenade de l'Alma 51 bte B1.43.031200 Bruxelles<br>Email: commission.ethique-saintluc@uclouvain.be<br>Tel: 02/ 764 55 14<br>https://www.saintluc.be/recherche/comite-ethique-accueil.php	Jean-Marie MALOTEAUX
Belgium	LEC - Site 0003	Comité d'éthique Hospitalo-facultaireErasme-ULB<br>Route de Lennik, 8081070 Bruxelles<br>Tel: +32 (0) 2 555 37 07;<br>Fax: +32 (0) 2 555 46 20<br>Email: Comite.ethique@erasme.ulb.ac.behttp://www.hopitalerasme.be/ethique	Prof. J.-M. BOEYNAEMs
Belgium	LEC - Site 0004	Commissie voor Medische ethiekUniversitair Ziekenhuis Gent,<br>C. Heymanslaan 10,9000 Gent<br>Tel: +32 9 332 22 66<br>Email: ethisch.comite@uzgent.be https://www.uzgent.be/nl/home/Paginas/home.aspx	Prof. Dr. D.MATTHYS
Belgium	LEC - Site 0005	Comité Medische Ethiek Ziekenhuis Oost-Limburg<br>Secretariaat Comité Medische EthiekSchiepse Bos 6<br>3600 GENK<br>Tel: 089 32 15 09;<br>Fax: 089 32 79 00 (clear mention CME)<br>Email: ec.submission@zol.be https://www.zol.be/comite-medische-ethiek	Patrick NOYENS
Bulgaria	CEC	Ethics Committee for Clinical Trials, 8, Damyan Gruev str., 1303 Sofia, Bulgaria	Stefan DIMITROV, MD
Bulgaria	LEC - Site 1501	Local Ethics Committee at UMHAT "Sv. Ivan Rilski" EAD, 15 Akad. I. Geshov Blvd.,1431 Sofia, Bulgaria,<br>Tel: +35929523554	Assoc. Prof. ZHELEV
Bulgaria	LEC - Site 1502	Local Ethics Committee at UMHAT "Tsaritsa Yoanna - ISUL" EAD, 8, ByaloMore Str., 1527 Sofia, Bulgaria,<br>Tel: +35929432170	Prof. Boryana DELIISKA
Bulgaria	LEC - Site 1503	Local Ethics Committee at "Acibadem CityClinic MHAT Tokuda"EAD, 51B NikolaVaptzarov Blvd, 1407 Sofia, Bulgaria,<br>Tel.: +35924034000	Rozalina BALABANSKA, MD
Bulgaria	LEC - Site 1504	Local Ethics Committee at UMHAT "SvetaAnna" - Sofia AD, 1, Dimitar Mollov Str., 1750 Sofia, Bulgaria	Nikolay DOBREV, MD
Bulgaria	LEC - Site 1505	Local Ethics Committee at Military Medical Academy - MHAT - Sofia, 3 Georgi Sofiyski Str., 1606 Sofia, Bulgaria	Col. Prof. Ventsislav MUTAFCHIISKI

Country	CEC or LEC for Site	Ethical Committees (or IRB)	Chair Person
Bulgaria	LEC - Site 1506	Local Ethics Committee at DCC "AlexanDr.ovska" EOOD, 1 Georgi Sofyiskistr., 1431 Sofia, Bulgaria,<br>Tel: +35929230815	Nataliya TEMELKOVA, MD
Bulgaria	LEC - Site 1507	Acibadem City Clinic University Hospital EOOD, UIC 202139132, with address of business 127 Okolovrasten pat Str., Sofia andcorresponding address of the Second clinical base: 66A Tsarigradsko shosse Blvd., 1784 Sofia, Bulgaria.	Dr Victoria Sasheva Dimitrova Biliana GENOVA*
Canada	Site 1301 and 1306	McGill University Health Centre (MUHC)REB<br>5100, boul. de Maisonneuve Ouest, 5th floorMontréal, Québec, H4A 3T2<br>Tel: 514-934-1934, ext-71461<br>Email: renaud.boulanger@muhc.mcgill.ca	PAGE Sonya; MANIATIS Thomas; LEBOUCHÉ Bertrand; BOULANGER Renaud; CANTINI Franca
Canada	Site 1302	Conjoint Health Research Ethics board(CHREB)<br>Research Services Office<br>2500 University Dr.ive NW, Calgary, AB,T2N1N4<br>Tel: (403) 220-7990; Fax: (403) 289-0693<br>Email: chreb@ucalgary.ca	STACEY A. Page, PhD
Canada	Site 1303	RESEARCH ETHICS OFFICE<br>HEALTH RESEARCH ETHICS BOARD<br>308 Campus Tower, 8625 – 112 St Edmonton, Alberta, Canada T6G IK8<br>Tel: 780.492.0459; Fax: 780.492.9429<br>Email: reoffice@ualberta.cawww.reo.ualberta.ca	Dr. Shane KIMBER<br>Dr. Glen PEARSON<br>Dr. Don MORRISH<br>Dr. Robert RENNIE<br>Dr. Anthony JOYCE
Canada	Site 1304	Western University<br>Health Science Research Ethics Board(HSREB)<br>Room 5150 Support Services Building, 1393Western Road<br>London, Ontario, Canada, N6G I G9<br>Tel: 519-661-2161<br>Email: ethics@uwo.ca	GILBERT, Joseph MD<br>(Jones, Philip MD Matsui, Doreen MD)
Canada	Sites 1305 and 1307	Advarra Institutional Review Board (IRB)–Advancing Better Research,<br>Stacey Neshevich phone 905-841-2257 Email: stacey.neshevich@advarra.com 372 Hollandview Trail, Suite 300, Aurora,ON L4G 0A5 Canada	Sara HARNISH, J.D., ExecutiveChair
Czech Republic	Site 0901 Urbanek	Etické komise<br>Fakultní nemocnice v MotoleV úvalu 84<br>150 06 Praha 5 - MotolCzech Republic<br>Tel.: +420 224 431 195; Fax: +420 224 431 196<br>Email: etickakomise@fnmotol.cz	MUDr. Vratislav ŠMELHAUS
Czech Republic	Site 0902 Sperl	Ethics Committee of IKEM and TNThomayer Hospital<br>Vídeňská 800<br>140 59 Prague 4 - KrčCzech Republic<br>Tel: 26108 3481<br>Email: alena.hruba@ftn.cz	Prof. MUDr. Vladimír STANĚK, CSc.
Czech Republic	Site 0903 Hejda	Research Site s.r.o.<br>Slovanská 27<br>326 00 Plzeň<br>Czech Republic<br>Tel - fax: +420 377 320 027<br>Email: eticka.komise@researchSite.cz	MUDr. Luboš JANŮ<br>Ph.D.
France	CEC - all Sites (No LEC)<br>[Sites 0201 to 0217]	Comité de Protection des Personnes Ouest IIIBat. Vie La Santé – 1er étage porte 101<br>CHU de Poitiers - 2 rue de la milétrie86021 Poitiers Cedex<br>Tel: 05.49.45.21.57; Fax: 05 .49.46.12.62<br>Email: cpp-ouest3@chu-poitiers.fr	Prof. Denis FRASCA
Germany	CEC - 0801 Schattenberg	Ethik-Kommission Landesärztekammer Rheinland-PfalzDeutschhausplatz 3 - 55116 Mainz<br>Postfach 29 26 - 55019 Mainz<br>Tel: 06131 28822-62; Fax: 06131 28822-66<br>Email: wagner@laek-rlp.de<br>Web: http://www.laek-rlp.de/ausschuesse-kommissionen/ethikkommission/	Univ.-Prof. Dr. med. Dipl. Ing. Stephan LETZEL
Germany	LEC - 0802 Geier	Ethics Committee of the University ofWürzburg<br>Institute for Pharmacology and ToxicologyVersbacher Str. 9<br>97078 Würzburg<br>Tel: 0931 31 48315; Fax: 0931 31 87520<br>Email: ethikkommission@uni-wuerzburg.de	Prof. Dr. med. R.JAHNS
Germany	LEC - 0803 Merle	Ethikkommission HeidelbergAlte Glockengießerei 11/1 69115 Heidelberg<br>Tel: +49 6221 56264-72; Fax: +49 6221 56264-80<br>Email: Marion.Teichmann@med.uni-heidelberg.de<br>http://www.medizinische-fakultaet-hd.uni-heidelberg.de/ethikkommission	Dr. med. Dr. hc Thomas STROWITZKI
Germany	LEC - 0805 Trautwein	Ethik-Kommission an der Medizinischen Fakultät der Rheinisch-Westfälischen Technischen Hochschule Aachen (RWTHAachen).<br>Universitätsklinikum AachenPauwelsstraße 30<br>52074 Aachen.<br>Tel: +49 241 80-89963; Fax: +49 241 80-82012<br>Email: ekaachen@ukaachen.de	Günther SCHMALZING

Country	CEC or LEC for Site	Ethical Committees (or IRB)	Chair Person
Germany	LEC 0807-Boettler	ETHIK-KOMMISSION<br>Albert-Ludwigs-Universität Freiburg Engelberger Straße 21, 79106 Freiburg<br>Tel: (+49) 0761 270-72500;<br>Fax: (+49) 0761 270-72630<br>Email: ekfr.amg@uniklinik-freiburg.de<br>http://www.ethik-kommission.uniklinik-freiburg.de	Prof. Dr. Rudolf KORINTHENBERG
Germany	LEC - 0808 Heinzow	Ethics Commission of the MedicalAssociation Westphalia-Lippe and the Westphalian Wilhelms University ofMünster<br>Gartenstrasse 210 - 214D-48147 Münster<br>Tel: +49 (0) 2 51/929 – 2460;<br>Fax: +49 (0) 2 51/929 - 2478<br>Email: ethikkommission @ aekwl.de	Univ.-Prof. Dr. med. Wolfgang E. BERDEL
Italy	CEC - 0101 Bugianesi	Comitato Etico Interaziendale A.O.U. Cittàdella Salute e della Scienza di Torino/ A.O.Ordine Mauriziano/ASL Città di Torino Corso Bramante 88/90<br>10126 - Torino<br>Tel: 011.633.6820; Fax: 011.633.4171<br>Email: comitatoetico@cittadellasalute.to.it	Dr. Marcello MADDALENA
Italy	LEC - 0102 Svegliati	Comitato Etico Regionale delle Marche Azienda Ospedaliero-Universitaria OspedaliRiuniti di Ancona<br>Via Conca n. 71<br>60126 Torrette di Ancona<br>Tel: +39 071 596 3667<br>Email: comitato.etico@ospedaliriuniti.marche.it	Prof. Paolo PELAIA
Italy	LEC - 0103 Miele	Comitato Etico<br>Fondazione Policlinico Universitario A.Gemelli<br>Università Cattolica del Sacro CuoreLargo Agostino Gemelli 8<br>00168 Roma<br>Fondazione Policlinico UniversitarioAgostino Gemelli IRCCS<br>Università Cattolica del Sacro Cuore Largo Agostino Gemelli 8, 00168 Roma<br>Tel: +39 06 30155556; Fax: +39 06 30155345<br>Email: comitato.etico@policlinicogemelli.it	Prof. Gigliola SICA
Italy	LEC - 0105 Craxi	Comitato Etico Palermo 1Via del Vespro 129 90127 Palermo<br>Tel: 0916555210; Fax: 0916553747<br>Email: bioetica@policlinico.pa.it	Prof. Salvatore LEONE
Italy	LEC - 0107 Lampertico	Comitato Etico Milano Area B Ospedale Maggiore Policlinico,Via F. Sforza n. 28<br>20122 Milano<br>Tel 02-55032982; Fax 02-55036618<br>Email federica.massacesi@policlinico.mi.it	Gaetana MUSERRA
Italy	LEC - 0108 Mangia	Comitato etico<br>Fondazione Casa Sollievo della SofferenzaIRCCS<br>Opera di San Pio da Pietrelcina 71013 San Giovanni Rotondo FG<br>Tel 0882 410831; Fax 0882410813<br>Email comitatoetico@operapaDr.epio.it	Luigi RENNA
Mauritius	1401	Name: Ethics committee<br>AdDr.ess: Bacha Building, 2nd floor, PortLouis<br>Tel: +230 52553636, +230 57525264<br>Fax: not available<br>email: kdhurmah@govmu.org	Dr. Satyabhoosun DOMAH
Poland	CEC and LEC - 0701 /Flisiak (National Coordinator)/ - Site did not screen patients	Komisja Bioetyczna Uniwersytetu Medycznego w Białym Stoku, Kilińskiego 1,15-089 Białystok<br>Tel: +48 85 748 54 07<br>Fax: +48 85 748 55 08<br>Email: prorektorkl@umb.edu.pl	Prof. Dr. hab. Otylia Kowal-BIELECKA
Poland	LEC - 0702 /Tomasiewicz/	Komisja Bioetyczna przy Uniwersytecie Medycznym w Lublinie, Al. Racławickie 1,20-059 Lublin;<br>Tel.: +48 81 448 52 13;<br>Fax: +48 81 448 52 11; Email:komisja.bioetyczna@umlub.pl	Dr. hab. n. med. Marcin OLAJOSSY
Poland	LEC - 0703 /Piekarska/	Komisja Bioetyki ds. Badań na ludziach przyUniwersytecie Medycznym, Pl. Hallera 1B, 90-647 Łódź;<br>Tel: +48 42 272 52 43, +48 42 272 52 44,<br>+48 785 911 596;<br>Email: bioetyka@umed.lodz.pl	Prof. Dr. hab. Józef Dr. ZEWOSKI
Poland	LEC - 0704 /Napora/	Dolnośląska Izba Lekarska we Wrocławiu Komisja Bioetyczna, Kazimierza Wielkiego45, 50-077 Wrocław;<br>Tel. +48 71 798 80 74, +48 607 552 143;<br>Email: kb@dilnet.wroc.pl	Dr. n. med. Włodzimierz BEDNORZ
Poland	LEC - 0705 /Hartleb/	Komisja Bioetyczna Śląskiego UniwersytetuMedycznego w Katowicach, Poniatowskiego15, 40-055 Katowice;<br>Tel. +48 32 208 36 42, +48 32 208 35 46;<br>Fax: +48 32 208 36 94;<br>Email: kombioet@sum.edu.pl	Prof. Dr. hab. n. med. Bogusław OKOPIEŃ

Country	CEC or LEC for Site	Ethical Committees (or IRB)	Chair Person
Slovenia	Site 1701	Republic of Slovenia National MedicalEthics Committee, Ministry of Health, Štefanova ulica 5, Sl-1000 Ljubljana;<br>Tel: +386 01 478 69 13; Fax: +386 01 478 60 58;<br>Email: kme.mz@gov.si	Dr. Božidar VOLJC
Slovenia	Site 1702	Republic of Slovenia National Medical Ethics Committee, Ministry of Health, Štefanova ulica 5, Sl-1000 Ljubljana; Tel:<br>+386 01 478 69 13; Fax: +386 01 478 60 58;<br>Email: kme.mz@gov.si	Dr. Božidar VOLJC
Spain	CEC - Sites 0501; 0502; 0503; 0504; 0505	CEIm Provincial de Sevilla<br>Avda. Dr. FeDr.iani, 3 – Unidad de Investigación 2ª planta Sevilla 41009 SevillaEspaña<br>Tel. 600 162 458; Fax. 955 00 80 15<br>Email: administracion.eecc.hvm.sspa@juntadeandalucia.es	Dr. Víctor Sánchez MARGALET
Switzerland	CEC Site 0401	Kantonale Ethikkommission für dieForschung<br>Murtenstrasse 31<br>3010 Bern<br>Tel: +41 31 633 70 70; Fax: +41 31 633 70 71<br>Email: info.kek.kapa@gef.be.chwww.be.ch/kek	Dr. med. Christian SEILER
Switzerland	Site 0402	Commission Cantonale d'éthique de larecherche Genève (CCER)<br>Rue ADr.ien-Lachenal 8<br>1207 Genève<br>Tel: +41 22 5465101<br>Email: ccer@etat.ge.ch	Prof. Bernard HIRSCHEL
Switzerland	Site 0403	Comitato etico cantonale Ticinoc/o Ufficio di sanità<br>Via Orico 5<br>6501 Bellinzona<br>Tel: +41 91 814 30 57<br>Email: dss-ce@ti.ch	Giovan Maria ZANINI
UK	REC - Sites 0301; 0302; 0303	North East - Newcastle & North Tyneside 1Research Ethics Committee<br>NHSBT Newcastle Blood Donor CentreHolland Drive<br>Newcastle upon TyneNE2 4NQ<br>Tel: 0207 104 8089<br>Email: newcastlenorthtyneside1.rec@hra nhs.uk	Mr Paddy STEVENSON
USA	LEC - Site 1607	DUHS Institutional Review Board2424 Erwin Road<br>Durham NC 919-668-5111	John FALLETTA
USA	LEC - Site 1603	IRB for Health Sciences Research University of Virginia PO box 800483Charlottesville VA 434-924-9634	MEDARD H.T. Ng
USA	LEC - Site 1612	UCSD Institutional Review Boards9452 Medical Center Drive<br>La Jolla CA 858-246-4777	Kip KANTELO
USA	LEC - Site 1604	Advarra IRB<br>6940 Columbia Gateway Drive #110 Columbia MD<br>410-884-2900	Tony DAVIS
USA	CEC - Sites 1609, 1616,1611, 1613, 1602, 1605, 1614, 1615, 1601, 1606	WIRB Copernicus IRB<br>5000 Centregreen Way STE 200Cary NC<br>888-303-2224	Donald A. DEIESO

*The activity of the Ethics committee for medical and scientific research established at Acibadem City Clinic University hospital EOOD terminated on 15 October 2018; NA: Not applicable; UK: United Kingdom; USA: United States of America.

Supplementary Table 2. Patient baseline demographics and characteristics, by treatment group (n = 247 patients)

Category<br>Parameter (unit)<br>n (%) or mean ± standard deviation		Placebo<br>-<br>N = 81	Lanifibranor<br>800 mg<br>N = 83	Lanifibranor<br>1200 mg<br>N = 83
Demographics	Female	41 (51%)	54 (65%)	49 (59%)
	Male	40 (49%)	29 (35%)	34 (41%)
	Age (years)	53.4 (13.1)	55.0 (10.4)	52.2 (13.8)
	White	74 (91.4%)	80 (96.4%)	78 (94.0%)
Body Mass Index (kg/m²)		32.8 (5.1)	32.5 (5.5)	33.3 (5.6)
	Obese	54 (67%)	49 (59%)	58 (70%)
Type 2 diabetes	Yes	35 (43%)	33 (40%)	35 (42%)
	No	46 (57%)	50 (60%)	48 (58%)
	If No, Prediabetes*	11 (24%)	20 (40%)	16 (33%)
Metabolic syndrome**		56 (69%)	64 (77%)	59 (71%)
	High waist circumference	78 (96%)	81 (98%)	75 (90%)
	High triglycerides	54 (67%)	48 (58%)	53 (64%)
	Low HDL-C	39 (48%)	36 (43%)	40 (48%)
	Pre-diabetic condition	46 (57%)	55 (66%)	52 (63%)
	Hypertension	49 (60%)	53 (64%)	51 (61%)
Insulin resistance	FIL (pmol/L)	234.0 ± 254.6	231.9 ± 191.9	274.7 ± 321.2
	HOMA-IR***	8.9 ± 9.6	9.5 ± 9.6	10.9 ± 14.7
Glycemic control	FG (mmol/L)	6.0 ± 1.6	6.2 ± 1.8	5.9 ±1.1
Lipid metabolism	Triglycerides (mmol/L)	2.0 ± 0.8	1.9 ± 0.9	2.0 ± 0.9
	HDL-C (mmol/L)	1.2 ± 0.3	1.3 ± 0.3	1.2 ± 0.3
	LDL-C (mmol/L)	2.9 ± 1.0	2.9 ± 1.1	2.9 ± 1.0
Apolipoprotein	APO-A1 (mg/dl)	139.5 ± 20.4	148.3 ± 24.8	141.5 ± 24.8
	APO-B (mg/dl)	110.1 ± 30.6	110.1 ± 31.3	108.23 ± 30.4
	APO-B/APO-A1	0.8 ± 0.3	0.8 ± 0.2	0.8 ± 0.3
	APO-C3 (ug/ml)	119.5 ± 43.1	125.1 ± 50.8	124.7 ± 45.2
Systemic inflammation	hs-CRP (mg/l)	4.0 ± 4.1	5.1 ± 6.2	5.4 ± 5.1
	Ferritin (µg/L)	254.6 ± 233.3	237.5 ± 238.2	230.9 ± 229.0
Blood pressure	Diastolic BP (mmHg)	80.8 ± 9.0	81.2 ± 10.6	80.5 ± 11.8
	Systolic BP (mmHg)	131.5 ± 15.0	131.5 ± 13.8	131.0 ± 13.6
Patient treated with statins at baseline		13 (16%)	18 (22%)	18 (22%)
	LDL-C (mmol/L) under statins	2.2 ± 0.8	2.3± 0.6	2.5 ± 1.0
	LDL-C (mmol/L) not under statins	3.0 ± 1.0	3.1 ± 1.1	3.0 ± 0.9

* Defined as FG level between 5.6 and 6.9 mmol/L (100 to 125 mg/dL);
** Metabolic syndrome criteria:

- High waist circumference (≥94 cm [37 in] in males, ≥80 cm [31.5 in] in females),
- High triglycerides (≥150 mg/dL (1.7 mmol/L) or hypertriglyceridemia / hyperlipidemia / dyslipidemia reported as medical history),
- Low HDL-C (<40 mg/dL (1.0 mmol/L) in males, <50 mg/dL (1.3 mmol/L) in females),
- Diabetic/pre-diabetic condition (presence of T2D, or FG ≥100 mg/dL [5.6 mmol/L] if no T2D),
- Hypertension (reported as medical history ongoing at baseline);

*** Patients treated with sulphonylureas were removed from HOMA-IR related analyses;
APO=apolipoprotein, BP=blood pressure, FG=fasting glucose, FIL=fasting insulin levels, HDL=high density lipoprotein, HOMA-IR=homeostasis model assessment of insulin resistance, hs-CRP=high-sensitivity C-reactive protein, LDL=low density lipoprotein, T2D=type-2 diabetes.

Supplementary Table 3. Glycemic control at EOT in patients without T2D at baseline, by treatment group

Parameter n (%)	Patients with prediabetes at baseline N = 41			Normoglycemic patients at baseline N = 83		
	Placebo	Lanifibranor 800 mg	Lanifibranor 1200 mg	Placebo	Lanifibranor 800 mg	Lanifibranor 1200 mg
Fasting glucose (EOT)						
N	9	18	14	31	24	28
<5.6 mmol/L	1 (11%)	12 (67%)	10 (71%)	23 (74%)	24 (100%)	28 (100%)
95%CI	[0% - 32%]	[45% - 88%]	[48% - 95%]	[59% - 90%]	[100% - 100%]	[100% - 100%]
[5.6-6.9] mmol/L	6 (67%)	5 (28%)	4 (29%)	8 (26%)	0 (0%)	0 (0%)
95%CI	[36% - 97%]	[7% - 48%]	[5% - 52%]	[10% - 41%]	-	-
>6.9 mmol/L	2 (22%)	1 (6%)	0 (0%)	0 (0%)	0 (0%)	0 (0%)
95%CI	[0% - 49%]	[0% - 16%]	-	-	-	-
p-values vs placebo*		0.013	0.009	-	0.007	0.005
Fasting insulin level status (EOT)						
N	8	18	14	29	24	26
Normal (≤173 pmol/L)	3 (37%)	14 (78%)	12 (86%)	19 (66%)	20 (83%)	24 (92%)
95%CI	[4% - 71%]	[59% - 97%]	[67% - 100%]	[48% - 83%]	[68% - 98%]	[82% - 100%]
Elevated (>173 pmol/L)	5 (63%)	4 (22%)	2 (14%)	10 (34%)	4 (17%)	2 (8%)
95%CI	[29% - 96%]	[3% - 41%]	[0% - 33%]	[17% - 52%]	[2% - 32%]	[0% - 18%]
p-values vs placebo**		0.078	0.052	-	0.103	0.010
HOMA-IR*** status (EOT)						
N	8	17	14	28	24	26
Normal (≤3)	0 (0%)	4 (23%)	3 (21%)	5 (18%)	10 (42%)	15 (58%)
95%CI	[0% - 0%]	[3% - 44%]	[0% - 43%]	[4% - 32%]	[22% - 61%]	[39% - 77%]
Elevated (>3)	8 (100%)	13 (77%)	11 (79%)	23 (82%)	14 (58%)	11 (42%)
95%CI	[100% - 100%]	[56% - 97%]	[57% - 100%]	[68% - 96%]	[39% - 78%]	[23% - 61%]
p-values vs placebo**		0.269	0.273	-	0.059	0.002

Population includes patients with available fasting glucose levels at baseline and EOT (n = 124); prediabetes is defined as fasting glucose level within 5.6–6.9 mmol/L; * p-values from two-sided Chi² test vs. placebo comparing glucose <5.6 mmol/L and ≥5.6 mmol/L; ** p-values from two-sided Chi² or Fisher tests vs. placebo; no adjustment for multiple comparisons was performed; *** patients treated with sulphonylureas were removed from HOMA-IR related analyses; CI=confidence interval, EOT=end of treatment, HOMA-IR=homeostatic model assessment of insulin resistance, T2D=type-2 diabetes.

Supplementary Table 4. High-sensitivity C-reactive protein over time, by treatment group (n = 247 patients)

		Placebo<br>-<br>N = 81	Lanifibranor<br>800 mg<br>N = 83	Lanifibranor<br>1200 mg<br>N = 83
Hs-CRP (mg/L) at baseline	N	80	83	83
	Mean ± SD	4.0 ± 4.2	5.1 ± 6.2	5.4 ± 5.1
	95%CI	[3.1; 5.0]	[3.7; 6.4]	[4.3; 6.5]
	Median	3.2	3.2	3.3
	Min; Max	0.3; 25.6	0.3; 36.6	0.3; 20.8
	p-values vs placebo*		0.361	0.101
Hs-CRP at baseline, in categories**	N	72	72	74
	<1 mg/L	11 (15%)	10 (14%)	9 (12%)
	95%CI	[13% - 32%]	[6% - 22%]	[6% - 21%]
	[1 – 3] mg/L	23 (32%)	26 (36%)	25 (34%)
	95%CI	[21% - 43%]	[25% - 47%]	[23% - 45%]
	>3 mg/L	38 (53%)	36 (50%)	40 (54%)
	95%CI	[41% - 64%]	[38% - 62%]	[43% - 65%]
Evolution from [1 – 3] mg/L	N	23	26	25
	[1 – 3] to <1 mg/L	3 (13%)	9 (35%)	11 (44%)
	95%CI	[0% - 27%]	[16% - 53%]	[25% - 63%]
	Stay [1 – 3] mg/L	15 (65%)	13 (50%)	12 (48%)
	95%CI	[46% - 85%]	[31% - 69%]	[28% - 68%]
	[1 – 3] to >3 mg/L	5 (22%)	4 (15%)	2 (8%)
	95%CI	[5% - 39%]	[2% - 29%]	[0% - 19%]
Evolution from >3 mg/L	N	38	36	40
	>3 to ≤3 mg/L	10 (26%)	16 (44%)	15 (38%)
	95%CI	[12% - 40%]	[28% - 61%]	[22% - 53%]
	Stay >3 mg/L	28 (74%)	20 (56%)	25 (63%)
	95%CI	[60% - 88%]	[39% - 72%]	[47% - 78%]

* p-values from two-sided Student or Wilcoxon tests, depending on the normality; no adjustment for multiple comparisons was performed; ** Cardiovascular risk is defined as high if hs-CRP >3 mg/L, intermediate if within 1-3 mg/L, and low if <1 mg/L; CI=confidence interval, EOT=end of treatment, hs-CRP=high-sensitivity C-reactive protein, SD=standard deviation.

Supplementary Table 5. CMH and hepatic markers absolute change from baseline according to weight-change group at EOT, by treatment group

EOT absolute change (unit) Mean ± SD [95%CI]	Weight relative change at EOT				
	Lanifibranor (800+1200 mg)			Placebo*	
	Stable (≤2.5%) N = 73	Moderate increase (2.5% - 5%) N = 23	Increase (≥5%) N = 48	Stable (≤2.5%) N = 61	Increase (≥2.5%) N = 12
Lipid metabolism and apolipoprotein levels					
TG (mmol/L)	-0.42 ± 0.97 [-0.64; -0.19]	-0.44 ± 0.57 [-0.68; -0.19]	-0.45 ± 0.60 [-0.63; -0.28]	0.03 ± 1.02 [-0.23; 0.30]	0.12 ± 0.71 [-0.33; 0.57]
HDL-C (mmol/L)	0.15 ± 0.23 [0.09; 0.20]	0.13 ± 0.23 [0.02; 0.23]	0.12 ± 0.20 [0.06; 0.18]	0.02 ± 0.20 [-0.03; 0.07]	0.01 ± 0.14 [-0.08; 0.10]
APO-B (mg/dL)	-9.66 ± 15.76 [-13.48; -5.85]	-13.04 ± 25.36 [-24.01; -2.08]	-14.56 ± 24.12 [-21.98; -7.13]	-2.58 ± 13.08 [-5.96; 0.80]	-0.08 ± 30.21 [-19.28; 19.11]
APO-B/ APO-A1	-0.08 ± 0.12 [-0.10; -0.05]	-0.06 ± 0.15 [-0.13; 0.00]	-0.07 ± 0.21 [-0.14; -0.01]	-0.01 ± 0.16 [-0.05; 0.03]	-0.01 ± 0.20 [-0.14; 0.12]
APO-C3 (µg/mL)	-10.72 ± 37.90 [-19.69; -1.75]	-7.30 ± 36.80 [-23.22; 8.61]	-9.33 ± 31.75 [-19.10; 0.45]	8.85 ± 37.76 [-0.82; 18.52]	19.08 ± 49.19 [-12.17; 50.34]
Insulin resistance					
FIL (pmol/L)	-122.6 ± 226.2 [-178.7; -66.6]	-98.1 ± 112.1 [-146.6; -49.6]	-155.2 ± 352.9 [-266.6; -43.8]	-24.8 ± 109.2 [-54.1; 4.4]	46.9 ± 110.2 [-31.9; 125.8]
HOMA-IR**	-5.6 ± 11.6 [-8.7; -2.5]	-5.3 ± 5.8 [-8.0; -2.6]	-5.3 ± 15.2 [-10.8; -0.5]	-0.4 ± 4.7 [-1.7; 1.0]	1.5 ± 4.4 [-1.9; 4.8]
Glycemic control					
FG (mmol/L)	-0.86 ± 1.34 [-1.17; -0.54]	-0.86 ± 0.81 [-1.21; -0.51]	-0.65 ± 1.76 [-1.17; -0.13]	0.26 ± 0.91 [0.02; 0.49]	0.04 ± 0.87 [-0.51; 0.59]
Systemic inflammation					
hs-CRP (mg/L)	-0.55 ± 4.82 [-1.67; 0.58]	-4.13 ± 7.61 [-7.42; -0.84]	-2.65 ± 4.57 [-3.98; -1.33]	0.63 ± 3.85 [-0.36; 1.62]	-0.08 ± 2.06 [-1.39; 1.23]
Ferritin (µg/L)	-64.95 ± 148.62 [-99.62; -30.27]	-74.83 ± 152.61 [-140.82; -8.83]	-84.44 ± 135.68 [-123.83; -45.04]	-13.05 ± 151.54 [-51.86; 25.76]	43.08 ± 134.97 [-42.67; 128.84]
Liver tests					
ALT (U/L)	-25.0 ± 48.5 [-36.3; -13.7]	-26.0 ± 22.1 [-35.6; -16.4]	-30.1 ± 40.2 [-41.7; -18.4]	-2.5 ± 29.9 [-10.2; 5.3]	20.9 ± 39.3 [-4.1; 45.9]
AST (U/L)	-10.9 ± 31.0 [-18.1; -3.6]	-12.9 ± 21.3 [-22.1; -3.7]	-21.0 ± 46.4 [-34.5; -7.5]	-1.2 ± 22.0 [-6.9; 4.5]	12.3 ± 20.6 [-0.7; 25.4]
GGT (U/L)	-33.2 ± 68.4 [-49.2; -17.3]	-28.0 ± 25.5 [-39.1; -17.0]	-40.8 ± 48.7 [-55.0; -26.7]	1.0 ± 22.1 [-4.6; 6.7]	12.0 ± 19.3 [-0.3; 24.3]
Adiponectin (µg/mL)	9.6 ± 10.4 [7.1; 12.1]	17.5 ± 20.6 [8.6; 26.4]	20.8 ± 16.6 [15.8; 25.8]	-0.2 ± 2.6 [-0.8; 0.5]	-0.5 ± 2.5 [-2.1; 1.2]
Diastolic BP (mmHg)	-3.3 ± 10.1 [-5.7; -1.0]	-1.9 ± 7.6 [-5.2; 1.4]	-4.2 ± 13.3 [-8.1; -0.4]	-0.8 ± 9.6 [-3.3; 1.7]	2.8 ± 8.9 [-2.9; 8.4]
NT-proBNP (pmol/L)	4.52 ± 10.85 [1.91; 7.12]	3.66 ± 8.39 [0.03; 7.29]	7.68 ± 13.62 [3.58; 11.77]	-0.98 ± 4.97 [-2.26; 0.29]	-0.73 ± 1.72 [-1.83; 0.36]
Steatosis					
CAP™ (dB/m)	-17.0 ± 55.6 [-33.1; -0.9]	-4.6 ± 61.4 [-40.1; 30.8]	-14.7 ± 55.1 [-34.6; 5.2]	-31.0 ± 8.0 [-21.4; 0.8]	-2.0 ± 61.5 [-4.4; 65.7]

Population includes patients with available weight change at EOT (n = 217 patients); * Only two weight change groups defined for placebo due to few patients ≥5% (n=4); ** patients treated with sulphonylureas were removed from HOMA-IR related analyses; APO=apolipoprotein, ALT=alanine aminotransferase, AST=aspartate aminotransferase, BP=Blood pressure, CAP™=controlled attenuation parameter, CI=confidence interval, CMH=cardiometabolic health, EOT=end of treatment, FG=fasting glucose, FIL=fasting insulin levels, GGT=gamma-glutamyl transferase, HDL=high density lipoprotein, HOMA-IR=homeostatic model assessment of insulin resistance, hs-CRP=high-sensitivity C-reactive protein, NT-proBNP=N-terminal pro b-type natriuretic peptide, SD=standard deviation, TG=triglycerides.

Supplementary Table 6. Adiponectin over time, by treatment group (n = 247 patients)

		Placebo<br>-<br>N=81	Lanifibranor<br>800 mg<br>N=83	Lanifibranor<br>1200 mg<br>N=83	Lanifibranor<br>Pooled<br>N=166	Total<br>N=247
Adiponectin (µg/mL) at baseline	N	81	80	83	163	244
	Mean ± SD	5.3 ± 4.2	5.0 ± 3.1	5.1 ± 3.2	5.1 ± 3.2	5.1 ± 3.5
	Median	4.6	4.2	4.4	4.3	4.5
	Min; Max	1.2; 30.8	1.3; 22.3	0.8; 14.7	0.8; 22.3	0.8; 30.8
Adiponectin at baseline	Low <5 µg/mL	48 (59%)	49 (61%)	50 (60%)	99 (61%)	147 (60%)
	Medium [5 – 10] µg/mL	27 (33%)	28 (35%)	23 (28%)	51 (31%)	78 (32%)
	High >10 µg/mL	6 (7%)	3 (4%)	10 (12%)	13 (8%)	19 (8%)
Adiponectin increase (fold) at EOT	N	72	66	73	139	211
	Mean ± SD	1.1 ± 0.3	3.8 ± 3.1	4.5 ± 3.5	4.2 ± 3.3	3.1 ± 3.1
	Median	1.0	2.8	3.5	3.0	2.1
	Min; Max	0.1; 2.5	0.8; 18.1	0.7; 17.4	0.7; 18.1	0.1; 18.1
Adiponectin increase at EOT	Unchanged <1.5-fold	65 (90%)	9 (14%)	4 (5%)	13 (9%)	78 (37%)
	Moderate [1.5 – 4] fold	7 (10%)	38 (58%)	38 (52%)	76 (55%)	83 (39%)
	High >4-fold	0 (0.0%)	19 (29%)	31 (42%)	50 (36%)	50 (24%)
EOT=end of treatment, Min=minimum, Max=maximum, SD=standard deviation.						

Supplementary Table 7. CMH and hepatic markers absolute change from baseline and histological improvement according to adiponectin-change group at EOT, in lanifibranor-treated patients

Category<br>EOT parameter (unit)<br>Mean ± standard deviation	Adiponectin fold at EOT		
	Unchanged <1.5-fold<br>N = 13	Moderate [1.5 – 4] fold<br>N = 76	High >4-fold<br>N = 50
Lipid metabolism and apolipoprotein levels			
Triglycerides (mmol/L)	-0.23 ± 0.62	-0.36 ± 0.96	-0.59 ± 0.51
HDL-C (mmol/L)	-0.03 ± 0.24	0.12 ± 0.19	0.22 ± 0.22
APO-B (mg/dL)	-3 ± 24	-9 ± 17	-19 ± 22
Insulin resistance			
FIL (pmol/L)	-17.3 ± 64.3	-115.4 ± 207.4	-177.8 ± 342.1
HOMA-IR*	-0.50 ± 3.04	-5.48 ± 10.72	-7.20 ± 14.88
Glycemic control			
Fasting glucose (mmol/L)	0.20 ± 0.79	-0.83 ± 1.33	-1.01 ± 1.63
HbA1c (%)	-0.1 ± 0.3	-0.4 ± 0.5	-0.6 ± 0.6
Systemic inflammation			
hs-CRP (mg/L)	1.02 ± 6.63	-1.73 ± 5.60	-2.90 ± 4.82
Ferritin (µg/L)	6.2 ± 177.3	-58.9 ± 99.4	-116.9 ± 183.7
Liver tests			
ALT (U/L)	11 ± 47	-28 ± 46	-35 ± 31
AST (U/L)	18 ± 53	-14 ± 28	-23 ± 38
GGT (U/L)	-4 ± 48	-35 ± 58	-45 ± 63
Steatosis			
CAP™ (dB/m)	16 ± 33	-0 ± 63	-37 ± 42
Histological improvement** [n (%)]			
CRN-NAS score improvement	5 (38.5%)	58 (85.3%)	44 (93.6%)
CRN-Steatosis improvement	4 (30.8%)	45 (66.2%)	37 (78.7%)
CRN-Inflammation improvement	4 (30.8%)	34 (50.0%)	30 (63.8%)
CRN-Ballooning improvement	4 (30.8%)	56 (82.4%)	43 (91.5%)
CRN-Fibrosis improvement	5 (38.5%)	26 (38.2%)	27 (57.4%)

Population includes lanifibranor-treated patients with available adiponectin change at EOT (n = 139); * patients treated with sulphonylureas were removed from HOMA-IR related analyses; ** Histological improvement is defined as at least one-point improvement according to NASH-CRN grading and staging; APO=apolipoprotein, ALT=alanine aminotransferase, AST=aspartate aminotransferase, CAP™=controlled attenuation parameter, CMH=cardiometabolic health, CRN=Clinical Research Network, EOT=end of treatment, FIL=fasting insulin levels, GGT=gamma-glutamyl transferase, HbA1c=hemoglobin A_{1c}, HDL=high density lipoprotein, HOMA-IR=homeostatic model assessment of insulin resistance, hs-CRP=high-sensitivity C-reactive protein, NAS=NAFLD activity score.

Supplementary Table 8. Absolute change in adiponectin (µg/mL) from baseline according to histological improvement at EOT, in lanifibranor-treated patients

Absolute change in adiponectin (µg/mL) at EOT<br>Mean ± standard deviation	Histological Improvement at EOT*		P-value**
	No	Yes	
CRN-NAS score	6.79 ± 10.29	16.42 ± 15.87	<0.001
CRN-Steatosis	8.92 ± 10.40	17.73 ± 16.74	0.002
CRN-Inflammation	11.89 ± 15.24	17.44 ± 15.33	0.003
CRN-Ballooning	7.45 ± 10.99	16.63 ± 15.92	<0.001
CRN-Fibrosis	12.40 ± 14.24	17.78 ± 16.51	0.024

Population includes lanifibranor-treated patients with available adiponectin and biopsy at baseline and EOT (n = 128); * Histological improvement is defined as at least one-point improvement according to NASH-CRN grading and staging; ** p-values from two-sided Student or Wilcoxon tests; no adjustment for multiple comparisons was performed; CRN=Clinical Research Network, EOT=end of treatment, NAS=NAFLD activity score.

Supplementary Table 9. Correlation between HOMA-IR decrease and liver tests improvement, in lanifibranor-treated patients

Spearman	ALT	AST	GGT
Coefficient	0.24	0.24	0.25
[95%CI]	[0.05 - 0.40]	[0.06 - 0.40]	[0.07 - 0.41]
two-sided p-value	0.011	0.011	0.008
Population includes lanifibranor-treated patients with available HOMA-IR and liver tests changes at EOT; patients treated with sulphonylureas were removed from the analyses (n = 127); no adjustment for multiple comparisons was performed; HOMA-IR=homeostatic model assessment of insulin resistance, ALT=alanine aminotransferase, AST=aspartate aminotransferase, CI=confidence interval, GGT=gamma-glutamyl transferase.			

Supplementary Table 10. Histological improvement according to HOMA-IR status at EOT, in lanifibranor-treated patients

Histological improvement* at EOT n (%)	HOMA-IR status at EOT		P-value**
	Normal (≤3) N = 44	Elevated (>3) N = 80	
CRN-NAS score improvement	38 (90.5%)	56 (76.7%)	0.066
CRN-Steatosis improvement	33 (78.6%)	44 (60.3%)	0.045
CRN-Inflammation improvement	27 (64.3%)	36 (49.3%)	0.120
CRN-Ballooning improvement	36 (85.7%)	56 (76.7%)	0.245
CRN-Fibrosis improvement	24 (57.1%)	27 (37.0%)	0.036

Population includes lanifibranor-treated patients with available HOMA-IR at EOT and biopsy at baseline and EOT; patients treated with sulphonylureas were removed from the analyses (n = 115); * histological improvement is defined as at least one-point improvement according to NASH-CRN grading and staging; ** p-values from two-sided Chi² or Fisher tests; no adjustment for multiple comparisons was performed; CRN=Clinical Research Network, EOT=end of treatment, HOMA-IR=homeostatic model assessment of insulin resistance, NAS=NAFLD activity score.

Supplementary Table 11. Correlation between HbA1c decrease and CMH markers improvement (relative changes), in lanifibranor-treated patients

Spearman	FIL	HOMA-IR*	FG	TG	HDL-C	APO-B
Coefficient	0.36	0.51	0.55	0.27	-0.21	0.23
[95%CI]	[0.20 - 0.50]	[0.36 - 0.63]	[0.42 - 0.65]	[0.12 - 0.42]	[-0.36 - -0.05]	[0.06 - 0.38]
two-sided p-value	<0.001	<0.001	<0.001	<0.001	0.011	0.008
Spearman	APO-B/APO-A1		ALT	AST	GGT	CAP™
Coefficient	0.26		0.35	0.28	0.33	0.17
[95%CI]	[0.10 - 0.41]		[0.20 - 0.49]	[0.12 - 0.42]	[0.18 - 0.47]	[-0.03 - 0.36]
two-sided p-value	0.002		<0.001	<0.001	<0.001	0.092

Population includes lanifibranor-treated patients with available HbA1c and CMH markers changes at EOT (n = 146); * patients treated with sulphonylureas were removed from HOMA-IR related analyses; no adjustment for multiple comparisons was performed.
APO=apolipoprotein, ALT=alanine aminotransferase, AST=aspartate aminotransferase, CAP™=controlled attenuation parameter, CMH=cardiometabolic health, FG=fasting glucose, FIL=fasting insulin levels, GGT=gamma-glutamyl transferase, HbA1c=hemoglobin A_{1c}, HDL=high density lipoprotein, HOMA-IR=homeostatic model assessment of insulin resistance, TG=Triglycerides.

Supplementary Table 12. Baseline CMH markers according to CAP™ at screening

Baseline parameter<br>Mean ± standard deviation [95%CI]	CAP™ at screening		P-value**
	≤ 302 dB/m<br>N = 48	> 302 dB/m<br>N = 125	
HOMA-IR*	7.8 ± 7.6 [5.3; 10.2]	11.9 ± 14.6 [9.1; 14.7]	0.003
HbA1c (%)	5.7 ± 0.6 [5.5; 5.9]	6.2 ± 0.8 [6.1; 6.3]	<0.001
Triglycerides (mmol/L)	1.72 ± 0.69 [1.52; 1.92]	2.04 ± 0.87 [1.89; 2.19]	0.012
HDL-C (mmol/L)	1.30 ± 0.27 [1.22; 1.38]	1.18 ± 0.27 [1.13; 1.23]	0.007
APO-A1 (mg/dL)	151 ± 25 [143; 158]	142 ± 22 [138; 146]	0.027
Diastolic blood pressure (mmHg)	77.5 ± 10.6 [74.5; 80.6]	82.3 ± 10.1 [80.5; 84.1]	0.007
ALT (IU/L)	56 ± 48 [42;70]	66 ± 36 [60; 72]	0.010
AST (IU/L)	38 ± 20 [32; 44]	52 ± 34 [46; 58]	0.003
Adiponectin (µg/mL)	5.8 ± 5.1 [4.3; 7.3]	4.9 ± 2.7 [4.4; 5.3]	0.583

Population includes patients with available CMH marker at baseline and CAP™ at screening (n = 171);
* patients treated with sulphonylureas were removed from HOMA-IR related analyses; ** p-values from two-sided Student or Wilcoxon tests; no adjustment for multiple comparisons was performed;
APO=apolipoprotein, ALT=alanine aminotransferase, AST=aspartate aminotransferase, CI=confidence interval, HbA1c=hemoglobin A_{1c}, HDL=high density lipoprotein, HOMA-IR=homeostatic model assessment of insulin resistance.

Supplementary Table 13. Lanifibranor treatment effect versus placebo on CMH and hepatic markers, according to obesity status (n = 247 patients)

Category<br>Parameters (unit), obesity status	Adjusted mean difference versus placebo at EOT (standard error), 95%CI, two-sided MMRM p-value	
	Lanifibranor 800 mg<br>$N_{Overall}$ = 83 / N_{T2D} = 33 / $N_{non\text{-}T2D}$ = 50	Lanifibranor 1200 mg<br>$N_{Overall}$ = 83 / N_{T2D} = 35 / $N_{non\text{-}T2D}$ = 48
Insulin resistance		
FIL (pmol/L), Ob<br>FIL (pmol/L), Nob	-102.23 (23.24),[-148.24; -56.23],<0.001<br>-32.40 (17.50),[-67.34; 2.55],0.068	-94.82 (22.70),[-139.75; -49.89],<0.001<br>-47.07 (18.29),[-83.60; -10.53],0.012
HOMA-IR*, Ob<br>HOMA-IR*, NOb	-5.38 (1.26),[-7.88; -2.89],<0.001<br>-0.89 (0.74),[-2.36; 0.59],0.233	-5.48 (1.21),[-7.89; -3.08],<0.001<br>-1.35 (0.77),[-2.90; 0.19],0.085
Glycemic control		
Fasting glucose (mmol/L), Ob<br>Fasting glucose (mmol/L), NOb	-1.24 (0.19),[-1.62; -0.86],<0.001<br>-0.66 (0.31),[-1.28; -0.04],0.039	-1.01 (0.18),[-1.37; -0.64],<0.001<br>-0.54 (0.33),[-1.20; 0.13],0.112
HbA1c (%), Ob<br>HbA1c (%), NOb	-0.60 (0.10),[-0.79; -0.41],<0.001<br>-0.21 (0.09),[-0.38; -0.05],0.014	-0.61 (0.09),[-0.79; -0.43],<0.001<br>-0.23 (0.09),[-0.41; -0.05],0.015
Lipid metabolism and apolipoprotein levels		
Triglycerides (mmol/L), Ob	-0.47 (0.14),[-0.74; -0.19],0.001	-0.42 (0.13),[-0.68; -0.15],0.003
Triglycerides (mmol/L), NOb	-0.69 (0.25),[-1.18; -0.19],0.008	-0.62 (0.26),[-1.15; -0.10],0.020
HDL-C (mmol/L), Ob	0.13 (0.04),[0.05; 0.21],0.001	0.09 (0.04),[0.02; 0.17],0.018
HDL-C (mmol/L), NOb	0.19 (0.06),[0.06; 0.31],0.003	0.11 (0.06),[-0.02; 0.24],0.086
LDL-C (mmol/L), Ob	-0.05 (0.12),[-0.29; 0.19],0.683	-0.13 (0.12),[-0.36; 0.10],0.259
LDL-C (mmol/L), NOb	0.15 (0.16),[-0.18; 0.47],0.364	0.38 (0.17),[0.03; 0.73],0.032
APO-A1 (mg/dL), Ob	-0.59 (3.49),[-7.48; 6.30],0.866	-5.01 (3.34),[-11.62; 1.60],0.136
APO-A1 (mg/dL), NOb	-0.50 (6.23),[-12.92; 11.93],0.937	-3.28 (6.39),[-16.02; 9.47],0.610
APO-B (mg/dL), Ob	-9.88 (3.54),[-16.87; -2.88],0.006	-11.95 (3.37),[-18.62; -5.28],0.001
APO-B (mg/dL), NOb	-8.08 (4.91),[-17.88; 1.72],0.105	-1.86 (5.38),[-12.60; 8.89],0.731
APO-B/APO-A1, Ob	-0.08 (0.03),[-0.14; -0.01],0.017	-0.08 (0.03),[-0.14; -0.02],0.011
APO-B/APO-A1, NOb	-0.07 (0.04),[-0.15; 0.01],0.106	-0.00 (0.04),[-0.09; 0.09],0.961
APO-C3 (ug/mL), Ob	-17.79 (7.01),[-31.66; -3.91],0.012	-19.45 (6.68),[-32.68; -6.23],0.004
APO-C3 (ug/mL), NOb	-20.56 (9.52),[-39.55; -1.57],0.034	-21.80 (9.94),[-41.62; -1.98],0.032
Systemic inflammation		
hs-CRP (mg/L), Ob	-2.49 (0.74),[-3.95; -1.04],0.001	-2.67 (0.71),[-4.08; -1.27],<0.001
hs-CRP (mg/L), Nob	-1.44 (1.25),[-3.93; 1.06],0.2542	0.80 (1.32),[-1.84; 3.44],0.5484
Ferritin (µg/L), Ob	-111.83 (25.83),[-162.92; -60.75],<0.001	-113.63 (24.82),[-162.72; -64.54],<0.001
Ferritin (µg/L), NOb	-27.99 (34.41),[-96.59; 40.61],0.419	23.47 (36.39),[-49.06; 96.01],0.521
Liver tests		
ALT (U/L), Ob	-27.52 (6.83),[-41.03; -14.01],<0.001	-26.19 (6.55),[-39.13; -13.24],<0.001
ALT (U/L), Nob	-19.46 (9.43),[-38.24; -0.68],0.042	-17.39 (10.06),[-37.45; 2.66],0.088
AST (U/L), Ob	-15.64 (4.68),[-24.92; -6.37],0.001	-15.24 (4.47),[-24.10; -6.38],0.001
AST (IU/L), NOb	-15.66 (10.50),[-36.81; 5.50],0.143	-6.64 (11.22),[-29.27; 15.98],0.557
GGT (U/L), Ob	-44.11 (7.01),[-57.97; -30.25],<0.001	-28.61 (6.65),[-41.76; -15.45],<0.001
GGT (U/L), NOb	-36.45 (11.55),[-59.55; -13.34],0.003	-29.90 (12.29),[-54.50; -5.31],0.018
Blood Pressure		
Diastolic (mmHg), Ob	-4.19 (2.02),[-8.18; -0.20],0.040	-1.49 (1.92),[-5.28; 2.31],0.439
Diastolic (mmHg), Nob	-2.91 (2.37),[-7.62; 1.80],0.223	-4.09 (2.53),[-9.13; 0.95],0.110
Systolic (mmHg), Ob	-3.49 (2.78),[-8.98; 2.01],0.211	0.68 (2.65),[-4.55; 5.92],0.796
Systolic (mmHg), Nob	-0.89 (3.31),[-7.49; 5.71],0.789	-2.82 (3.51),[-9.83; 4.18],0.424
Steatosis		
CAP™ (dB/m), Ob	2.43 (10.30),[-18.02; 22.87],0.814	-21.81 (10.01),[-41.70; -1.93],0.032
CAP™ (dB/m), Nob	-40.83 (14.46),[-69.92; -11.75],0.007	-29.79 (17.27),[-64.53; 4.96],0.091

* Patients treated with sulphonylureas were removed from HOMA-IR related analyses; Resulting from MMRM models using change from baseline as endpoint, the time, treatment, the diabetic status, the interaction (treatment*time) and the baseline value as fixed effects, a time repeated measure within each subject and an unstructured variance covariance matrix; no adjustment for multiple comparisons was performed. Similar results were obtained when considering concomitant use of metformin and statins as covariates in the statistical model [results not shown]; APO=apolipoprotein, ALT=alanine aminotransferase, AST=aspartate aminotransferase, CAP™=controlled attenuation parameter, CI=confidence interval, CMH=cardiometabolic health, EOT=end of treatment, FIL=fasting insulin levels, GGT=gamma-glutamyl transferase, HbA1c=hemoglobin A_{1c}, HDL=high density lipoprotein, HOMA-IR=homeostatic model assessment of insulin resistance, hs-CRP=high-sensitivity C-reactive protein, LDL=low density lipoprotein, MMRM=mixed model for repeated measures.

Supplementary Figures

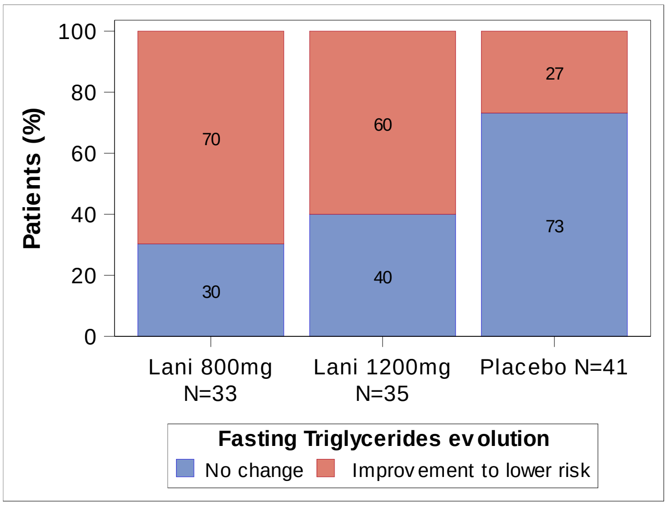

Supplementary Figure 1. Improvement of triglycerides at EOT according to treatment groups, in patients with high baseline values (>1.7 mmol/L), (n = 109 patients).
Triglycerides improvement to lower risk is indicated as follows: decrease below ≤1.7 mmol/L, red; no change otherwise, blue. Source data are provided as a Source Data file. EOT=end of treatment, Lani=Lanifibranor.

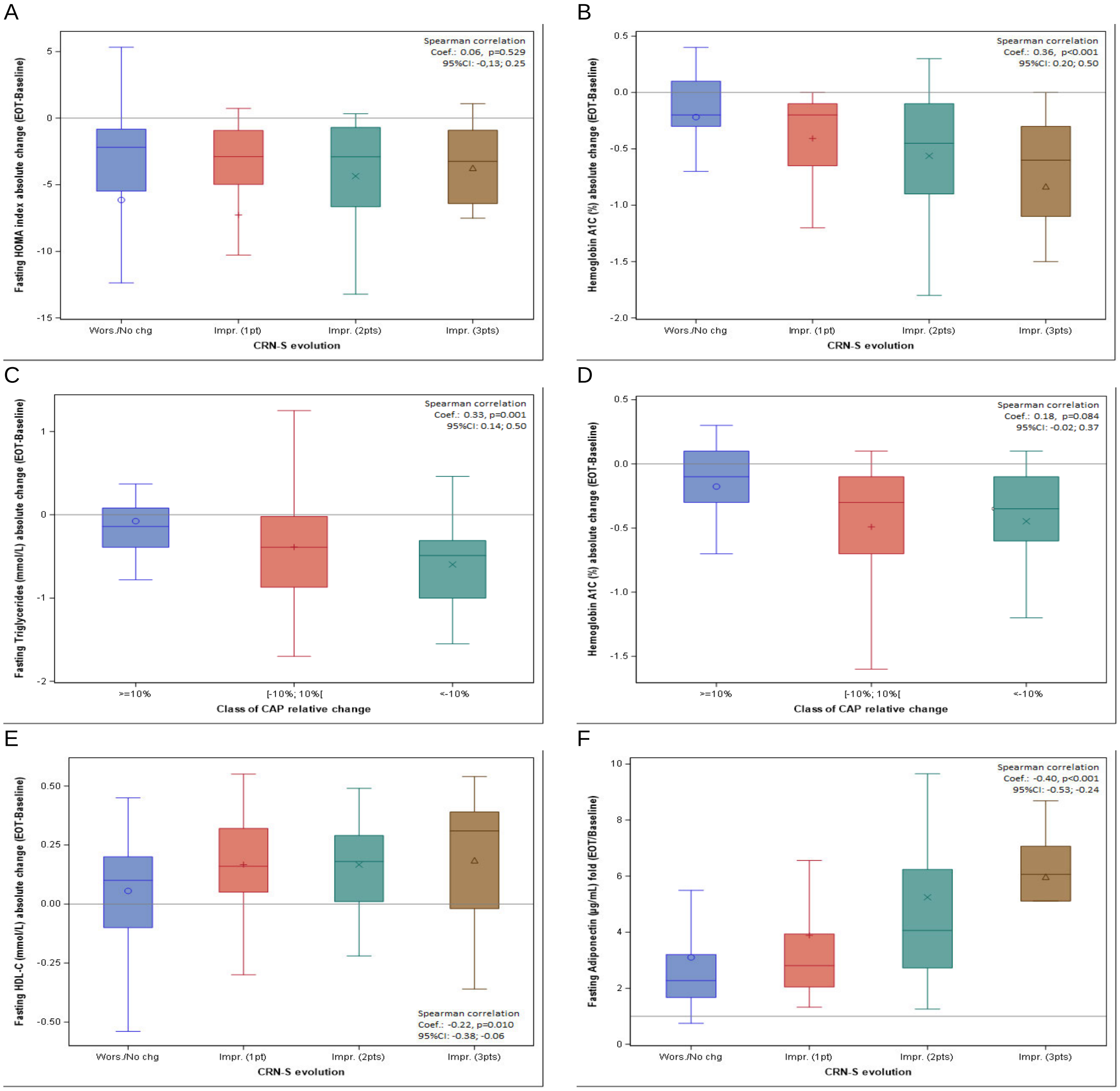

Supplementary Figure 2. CMH change from baseline according to hepatic steatosis improvement at EOT.

(**A**) HOMA-IR (n = 106 patients), (**B**) (n = 134 patients) and (**D**) HbA1c (n = 94 patients), (**C**) Triglycerides (n = 93 patients), (**E**) HDL-C (n = 133 patients) and (**F**) adiponectin (n = 128 patients). The box's length represents the IQR, the symbol (circle, plus, cross, triangle) inside the box represents the mean, the horizontal line inside the box represents the median, the whiskers extend to the minimum and maximum values included in [Q1-1.5*IQR; Q3+1.5IQR]. **A**, **B**, **E** and **F**: CRN-S evolution is indicated as follows: Wors/No chg, blue; Impr. (1pt), red; Impr. (2pts), green; Impr. (3pts), brown. **C** and **D**: CAP^{TM} relative change groups are indicated as follows: worsening ≥10%, blue; no change (within -10% and 10%), red; improvement ≥10%, green. P-values from two-sided Spearman correlation test using the corresponding continuous data. No adjustment for multiple comparisons was performed. Patients treated with sulphonylureas were removed from HOMA-IR related figures. Source data are provided as a Source Data file. CAP™ (or CAP)=controlled attenuation parameter, chg=change, CI=confidence interval, Coef.=coefficient, CRN=Clinical Research Network, EOT=end of treatment, HbA1c=Hemoglobin A1c, HDL=high density lipoprotein, HOMA-IR=homeostatic model assessment of insulin resistance, IQR=interquartile range, Impr.=improvement, p=p-value, pt=point Wors.=Worsening.

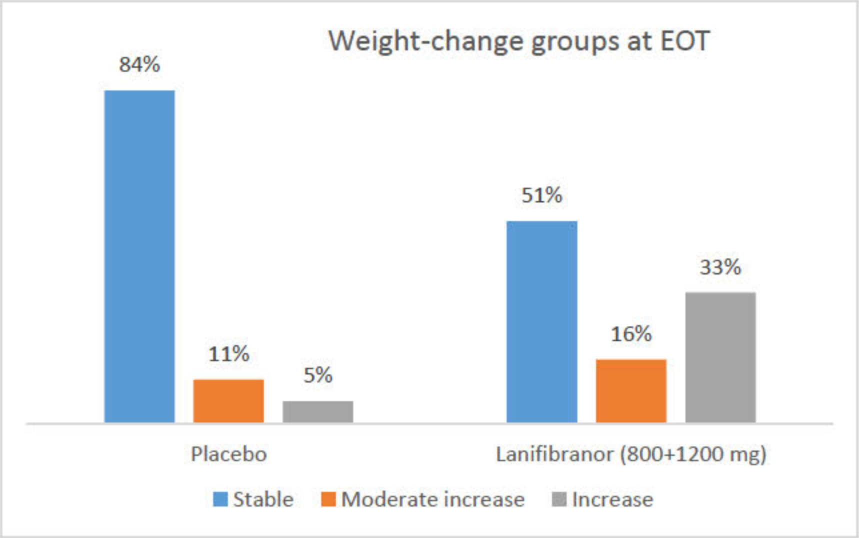

Supplementary Figure 3. Weight relative change at EOT, by treatment group, (n = 21 patients). Weight relative change groups are indicated as follows: stable (≤2.5%), blue; moderate (within 2.5% and 5%), orange; increase (≥5%), grey. Source data are provided as a Source Data file. EOT=end of treatment.

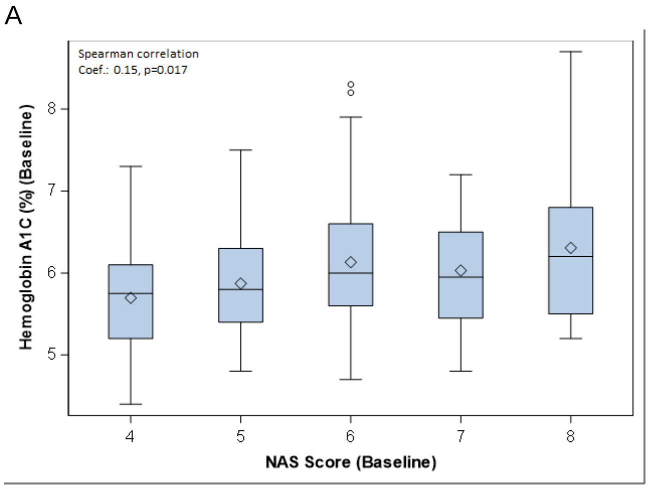

Supplementary Figure 4. Correlation of (A) MASH activity and (B) fibrosis with HbA1c, at baseline, (n = 245 and 241 patients, respectively).

The box's length represents the IQR, the diamond inside the box represents the mean, the horizontal line inside the box represents the median, the whiskers extend to the minimum and maximum values included in [Q1-1.5*IQR; Q3+1.5IQR], the circles outside the whiskers represent the outliers. P-value from two-sided Spearman correlation test. No adjustment for multiple comparisons was performed. Source data are provided as a Source Data file. Coef.=coefficient, HbA1c=hemoglobin A1c, IQR=interquartile range, NAS score=Clinical Research Network NAFLD activity score, p=p-value.

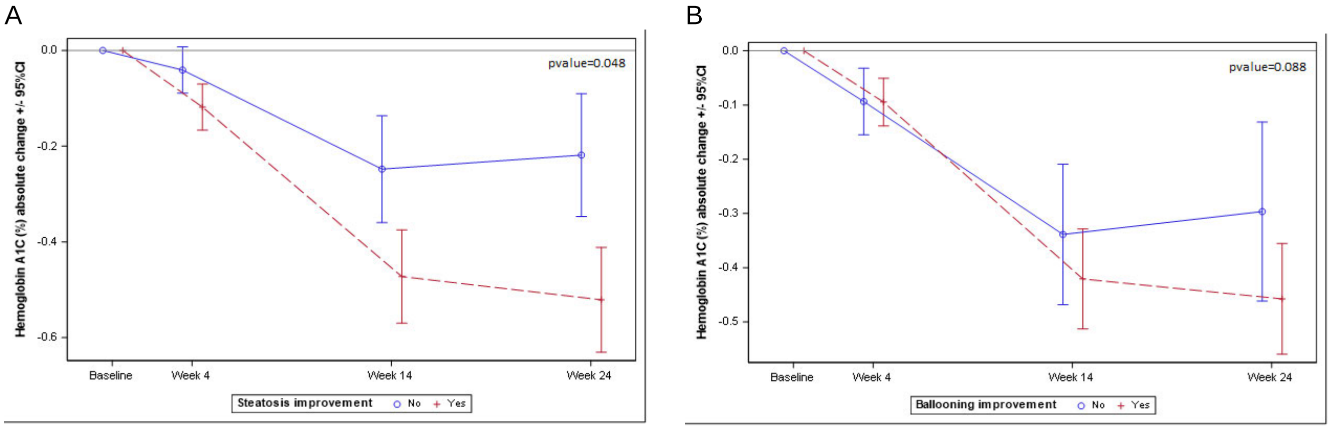

Supplementary Figure 5. Time course for HbA1c absolute change from baseline according to histological improvement at EOT, in lanifibranor-treated patients, (n = 142 patients).
(**A**) by CRN-Steatosis improvement (**B**) by CRN-Ballooning improvement. Data are plotted as mean ± 95%CI. Histological improvement groups are indicated as follows: improvement of at least one point at EOT compared to screening according to NASH-CRN grading, red; and no improvement otherwise, blue. P-values from two-sided Mixed Model for Repeated Measures using change from baseline as endpoint, and the histological improvement and baseline value as fixed effects. No adjustment for multiple comparisons was performed. Source data are provided as a Source Data file. CI=confidence interval, EOT=end of treatment, HbA1c=hemoglobin A1c.

STUDY PROTOCOL

Protocol identification: IVA_01_337_HNAS_16_002

EudraCT number: 2016-001979-70

Version 5.1 – March 25th, 2020

Study title

A randomized, double-blind, placebo-controlled, multicenter, dose-range, proof-of-concept, 24-week treatment study of IVA337 in adult subjects with nonalcoholic steatohepatitis (NASH)

Investigational medicinal product: IVA337 (lanifibranor)

Development phase: IIb

Study initiation date: Q1 2017

Study completion date: Q1 2020

Principal Investigators: **Sven Francque**, MD, PhD, Division of Gastroenterology and Hepatology, Antwerp University Hospital, Wilrijkstraat 10, B-2650 Edegem, Belgium

Manal F. Abdelmalek, MD, MPH, Professor of Medicine, NAFLD Clinical Research Program, Division of Gastroenterology / Hepatology, Duke University, 40 Duke Medicine Circle, Clinic 2H / 2J, Durham, NC 27710, USA

Sponsor: Inventiva S.A., 50 rue de DIJON, 21121 Daix, France

Sponsor Chief Medical Officer: [redacted]

Confidentiality statement

This information may not be used, published, or otherwise disclosed without the prior written authorization from the Sponsor.

Revision history

Version	Date	Main Revision summary
Version 1.0	June 28th, 2016	Initial version
Version 1.1	August 19th, 2016	Pregnancy test and IMP hypersensitivity inclusion/exclusion criteria update.<br>Add of "non treatment-emergent AE" definition.<br>"End of trial" definition update
Version 1.2 (France only)	January 5th, 2017	Addition of CPK and osteopenia as exclusion criteria
Version 1.2 (Switzerland only)	February 10th, 2017	Inclusion criteria 8 update (Contraceptive method)
Version 1.2 (Germany only)	February 10th, 2017	Inclusion criteria 9 update
Version 1.3 (Switzerland only)		Recruitment period update
Version 2.0	March 2nd, 2016	Update inclusion/exclusion criteria list with all country specific requests
Version 3.0	August 3rd, 2017	Recruitment period update
Version 4.0	November 2nd, 2017	Add pre-treatment liver biopsy as screening procedure when no biopsy performed less than 6 months before screening is available<br>Update of study screening procedures (including Liver MultiScan in selected sites)
Version 4.1	September 14th, 2018	Adding sites in the US, minor update of a few inclusion/non-inclusion criteria, corrections of typo errors and adaptation to US sites. Version applicable for US only.
Version 4.2	December 12th, 2018	Adding urine dipstick, to be performed on site, at each visit when a urine sample is collected.
Version 5.0	July 09th, 2019	Updating the secondary endpoints, safety and biomarkers analysis performed, clarification of some inclusion / exclusion parameters, clarification of prohibited concomitant medications (i.e. dosages, routes, washout period if relevant).
Version 5.1	March 25th, 2020	Updating exploratory parameters for immunohistochemistry endpoints

Synopsis

Study title: A randomized, double-blind, placebo-controlled, multicenter, dose-range, proof-of-concept, 24-week treatment study of IVA337 in adult subjects with nonalcoholic steatohepatitis (NASH).

Study code: IVA_01_337_HNAS_16_002.

Trial registration: 2016-001979-70

Protocol version and date: Version 5.1, (March 25th, 2020)

Development phase: IIb, Proof of Concept

Investigators & study location: Principal Investigators: **Pr. Sven Francque**, Antwerp University Hospital, Wilrijkstraat 10, B-2650 Edegem, Belgium.; Pr. **Manal F. Abdelmalek**, MD, MPH, Division of Gastroenterology / Hepatology, Duke University, 40 Duke Medicine Circle, Clinic 2H / 2J, Durham, NC 27710, USA.

Multi-center, at least 60 sites across Europe, United States of America (USA) and other countries worldwide (incl. Canada, Australia and Republic of Mauritius).

Studied period: First enrolment: Q1 2017. Last Patient Last Visit: Q1 2020.

Objectives: To assess the safety and the efficacy on the activity part of the Steatosis Activity Fibrosis (SAF) histological score (inflammation and ballooning) of a 24-week treatment with two doses of lanifibranor (800, 1200 mg/24h) in NASH adult patients.

Study design: Randomized (stratified on diabetes), placebo-controlled, double-blind, parallel-assignment, dose-range multicenter study.

Study duration: For each patient, the study duration will be an overall of 6 to 8 months (with a 3-day to 4-week selection period, a 24-week treatment period and a 4-week follow-up period).

Total number of subjects: At least 300 patients will be screened, to randomize 225 patients (75 patients/arm).

Diagnosis and main criteria for inclusion:

1. Adult subjects, age ≥18 years.
2. NASH histological diagnosis according to the currently accepted definition of both EASL and AASLD, requiring the combined presence of steatosis (any degree ≥ 5%) + lobular inflammation of any degree + liver cell ballooning of any amount, on a liver biopsy performed ≤ 6 months before screening in the study or at screening, and confirmed by central reading during the screening period *and*
    a. SAF Activity score of 3 or 4 (>2)
    b. SAF Steatosis score ≥ 1
    c. SAF Fibrosis score < 4
3. Subject agrees to have a liver biopsy performed after 24 weeks of treatment.
4. Compensated liver disease with the following hematologic and biochemical criteria before randomization:
    - ALT < 10xULN
    - Hemoglobin ≥ 110 g/L (11g/dL) for females and ≥ 120 g/L (12g/dL) for males

- White blood cell (WBC) > 2.5 10^9/L (2.5 10^3/µL)
- Neutrophil count > 1.5 10^9/L (1.5 10^3/µL)
- Platelets > 100 10^9/L (100 10^3/µL)
- Total bilirubin < 35 µmol/L (2.06 mg/dL). Patients with bilirubin ≥ 35 µmol/L (2.06 mg/dL) can be included if non-conjugated bilirubin in the setting of a Gilbert's syndrome.
- Albumin > 36 g/L (3.6 g/dL)
- International Normalized Ratio (INR) < 1.4
- Serum creatinin < 115 µmol/L (1.3 mg/dL) (men) or < 97 µmol/L (1.1 mg/dL) (women) or estimated glomerular filtration rate ≥ 60 mL/min/1.73m2

5. No other causes of chronic liver disease (autoimmune, primary biliary cholangitis, HBV, HCV, Wilson's, α-1-antitrypsin deficiency, hemochromatosis, etc…), considered to have an impact on the patient's safety or on the efficacy evaluation.
6. If applicable, have a stable type 2 diabetes, defined as HbA1c ≤ 8.5% and fasting glycemia <10 mmol/L (180 mg/dL), no introduction of new medication in the previous 6 months, and no new symptoms associated with decompensated diabetes in the previous 3 months. Minor modifications of anti-diabetic treatments or dosages are allowed if done in a context of stable type 2 diabetes, i.e. HbA1c ≤ 8.5% in the previous 6 months.
7. Have a stable weight since the liver biopsy was performed, defined by no more than a 5% loss of initial body weight.
8. Negative pregnancy test or post-menopausal. Women with childbearing potential (i.e. fertile, following menarche and until becoming post-menopausal unless permanently sterile) must be using a highly effective method of contraception (i.e. combined (estrogen and progestogen containing) hormonal/ progestogen-only hormonal contraception associated with inhibition of ovulation, intrauterine device, intrauterine hormone-releasing system, bilateral tubal occlusion, vasectomised partner). The contraceptive method will have to be followed for at least one menstruation cycle after the end of the study.
9. Subjects having given her/his written informed consent.

Exclusion criteria:

1. Evidence of another form of liver disease, considered to have an impact on the patient's safety or on the efficacy evaluation.
2. History of sustained excess alcohol ingestion in the year before the pre-study treatment biopsy: daily alcohol consumption > 30 g/day (3 drinks per day) for males and > 20 g/day (2 drinks per day) for females.
3. Unstable metabolic condition: Weight change > 5% in the last three months, diabetes with poor glycemic control (HbA1c > 8.5%), introduction of an antidiabetic or of an anti-obesity drug or restrictive bariatric (weight loss) surgery in the past 6 months prior to screening.
4. History of gastrointestinal malabsorptive bariatric surgery within less than 5 years or ingestion of drugs known to produce hepatic steatosis including oral corticosteroids (at dose >5mg/day prednisone equivalent), estrogens (at doses greater than those used for contraception or hormone replacement), tamoxifen, methotrexate, tetracycline or

amiodarone in the previous 6 months. Corticosteroids administered by routes other than oral are allowed. One short (<2 weeks) course of oral corticosteroids, more than 3 months before the pre-study treatment biopsy is also allowed.

5. Significant systemic or major illnesses other than liver disease, including congestive heart failure (class C and D of the AHA), unstable coronary artery disease, cerebrovascular disease, pulmonary disease, renal failure, organ transplantation, serious psychiatric disease, malignancy that, in the opinion of the Investigator, would preclude treatment with lanifibranor and/or adequate follow up.
6. HBs antigen >0, HCV PCR >0 (patients with a history of HCV infection can be included if HCV PCR is negative since more than 3 years), HIV infection.
7. Pregnancy or lactation or inability to adhere to adequate contraception in women of child-bearing potential.
8. Active malignancy except cutaneous basocellular carcinoma.
9. Any other condition, which, in the opinion of the Investigator would impede competence or compliance or possibly hinder completion of the study.
10. Body mass index (BMI) >45 kg/m^2.
11. Type 1 diabetes and type 2 diabetic patient on insulin.
12. Diabetic ketoacidosis.
13. Fasting Triglycerides > 300 mg/dL (3.39 mmol/L).
14. Hemostasis disorders or current treatment with anticoagulants.
15. Contra-indication to liver biopsy.
16. History of, or current cardiac dysrhythmias and/or a history of cardiovascular disease event, including myocardial infarction, except patients with only well controlled hypertension. Any clinically significant ECG abnormality reported by central ECG reading, confirmed by the Investigator to be Clinically Significant.
17. Participation in any other investigational drug study within the previous 3 months.
18. Have a known hypersensitivity to any of the ingredients or excipients of the IMP including: Lactose monohydrate, Hypromellose, Sodium laurilsulfate, Sodium starch glycolate (type A), Magnesium stearate, Opadry™ II 85F18422
19. Be possibly dependent on the Investigator or the Sponsor (e.g., including, but not limited to, affiliated employee).
20. Creatine phosphokinase (CPK) >5 x ULN.
21. Patient with history of well-documented osteopenia. Patient treated with vitamin D and/or Calcium based supplements for preventive reasons can be included.

(The criteria below are applicable only for patients who will undergo a MRI/LMS in selected centers)

22. Claustrophobia to a degree that prevents tolerance of MRI scanning procedure. Sedation is permitted at discretion of Investigator.
23. Metallic implant of any sort that prevents MRI examination including, but not limited to: aneurysm clips, metallic foreign body, vascular grafts or cardiac implants, neural

stimulator, metallic contraceptive device, tattoo, body piercing that cannot be removed, cochlear implant; or any other contraindication to MRI examination.

Test product, dose and mode of administration:

Tablets of lanifibranor 400 mg, two doses of lanifibranor (800 and 1200 mg QD), i.e. 3 tablets of 400 mg/placebo per os, during breakfast, once daily (QD) will be evaluated.

Prohibited concomitant medications:

<u>If not specified, the following treatment must be stopped at the latest before randomization.</u>

- PPAR Gamma agonists, PPAR Alpha agonists (fibrates), ezetimibe,
- Bile salts chelators, Phytosterols, fish oils,
- Glucagon like peptide-1 receptor agonists (incl. liraglutide, exenatide),
- Insulin,
- Vitamins E (alpha-tocopherol),
- Anticoagulants (incl. warfarin, dabigatran, rivaroxaban, apixaban),
- Oral corticosteroids.

Allowable medications for standard care or precautions:

- ***Obesity***: stable weight since the liver biopsy was performed, defined by no more than a 5 % loss of initial body weight (see inclusion criteria 7).
- ***Treatment used for the underlying medical condition***

  Treatments are allowed within certain restrictions (described above and below) and provided that they have been kept at ***<u>stable doses for at least 6 months before the pre-treatment biopsy</u>***.

  Type 2 diabetes:

  - Metformin,
  - Dipeptidyl peptidase-4 inhibitors,
  - Sodium-glucose transport protein 2 inhibitors: canagliflozin, dapagliflozin and empagliflozin.

  Minor modifications of anti-diabetic treatments or dosages are allowed if done in the context of stable type 2 diabetes, i.e. HbA1c ≤ 8.5% in the previous 6 months.

  Hyperlipidemia: only statins at stable doses within the past 3 months before pre-study treatment biopsy will be allowed.

  Antiplatelets agents: The antiplatelets agents (incl. low-dose aspirin, ticlopidine, clopidogrel, prasugrel, ticagrelor) are allowed.

  Herbal supplements and Others Supplementation: Herbal preparations or vitamin supplements with no exact composition known should not be started during the course of the study as they could be liver toxic.

- ***Other Medications***

  Medications other than the IMP and those mentioned above should only be started during the course of the study with the agreement of the Investigator in order to avoid interference with study assessments. The need for other medication may lead to the exclusion of the patient from the study.

If a symptomatic medication is needed to treat adverse events related to IMP, the Investigator will inform the Sponsor about the concomitant medication given.

Duration of treatment: IMP will be given for 24 weeks.

Parameters assessed during the study: Continuous recording of adverse events and concomitants therapies, physical examination, vital signs, ECG (12-leads), haematology, blood biochemistry and urinalysis, liver enzymes, QOL questionnaires including the Flinders Fatigue scale (FFS) and the SF-36v2 health survey, Transient Elastography (TE) (mandatory before the liver biopsy, when performed at screening) and Controlled Attenuation Parameter (CAP), central reading of liver biopsies, biobank for specific markers of inflammation, lipids, glucids, fibrosis, bone remodeling and lanifibranor plasma trough levels and only at screening in selected sites Magnetic Resonance Imaging (MRI) /Liver MultiScan (LMS) measuring Liver Inflammation and Fibrosis (LIF) score and hepatic fat content MRI-PDFF (proton density fat fraction).

Primary efficacy criterion: SAF activity score, i.e. the addition of hepatocyte ballooning score (0 to 2) and lobular inflammation score (0 to 2). Assessment of the difference between any verum groups versus placebo.

Statistical methods and Safety:

Sample size*:* The expected rate of responders (decrease of 2 points of SAF activity score) was estimated to 10% in the placebo group by a panel of clinical expert. An excess rate of responders of 20% was assessed beyond any doubt as clinically pertinent since the chance of being responder is three time larger in the best dose and the Number Needed to Treat (NNT) is as low as 5. The sample required to reach a power of at least 80% is 72 patients per group with a two-sided alpha of 0.025 (adjustment for multiplicity) and has been rounded to 75. Consequently, 225 patients will be randomized.

Randomization*:* Patients will be randomized 1:1:1 (Placebo, 800 mg lanifibranor, 1200 mg lanifibranor) and stratified on presence/absence of type 2 diabetes to receive either dose of lanifibranor or a matching placebo.

Safety variables*:* The review of safety and tolerance will be performed on the safety population. The safety analysis will be based on the reported AEs and other safety information. The effect of the demographic differences (gender, age, etc.) and risk factors of clinical relevance will be explored.

Efficacy variables:

Primary endpoint: The primary endpoint is a binary outcome (responder / non responder). A responder is defined as a decrease from baseline to week 24 of at least 2 points of the SAF activity score combining hepatocellular inflammatory and ballooning score without fibrosis progression. Responder rates will be compared between the placebo and IMP groups at the end of the treatment period (week 24) using a Cochran Mantel Haenzel test stratified on diabetes.

Secondary endpoints:

The following changes from baseline to 24 weeks of treatment will be evaluated:

- NASH improvers defined as subjects with a decrease of at least 2 points in NAS, and no worsening in fibrosis from baseline to end of treatment (week 24).

- Percent of patients with resolution of NASH (Steatosis without ballooning and with or without mild inflammation) and no worsening of fibrosis) from baseline to end of treatment (week 24).
- Percent of patients with a change in components of SAF score from baseline to end of treatment (week 24):
    - Steatosis: -1 point
    - Lobular inflammation: -1 point
    - Ballooning: -1 point
- Percent of patients with at least 1- point improvement of fibrosis score on a 4-point scale (SAF) without worsening of NASH, defined as no increase for ballooning, or inflammation, or steatosis, using the SAF scoring system, from baseline to end of treatment (week 24).
- Change in fibrosis score on a 4-points scale (SAF) and modified Ishak: - 1 point from baseline to end of treatment (week 24).
- Liver enzymes (ALT, AST, γGT) change from baseline to end of treatment (week 24).
- Inflammatory markers (fibrinogen, hs-CRP, alpha2 macroglobulin and haptoglobin levels) change from baseline to end of treatment (week 24).
- Glucose metabolism (fasting glucose and insulin, HOMA index and, in subjects with T2DM, HbA1c) change from baseline to end of treatment (week 24).
- Main plasma lipids levels (TC, HDL-C, calculated LDL-C, TG and apoA1) change from baseline to end of treatment (week 24).
- Adiponectin change from baseline to end of treatment (week 24).

Exploratory endpoints:

- Specific markers of inflammation, lipids, glucids, fibrosis, bone remodeling change from baseline to end of treatment (week 24).
- Transient Elastography (TE) and Controlled Attenuation Parameter (CAP) change from baseline to end of treatment (week 24)
- If deemed necessary: Immunohistochemistry: change in the semiquantitative score of ballooning, stellate cell activation, or any other analysis which will be considered relevant in the scope of the study, i.e. to understand the safety and efficacy of lanifibranor in patients with NASH, from baseline to end of treatment (week 24).

The Hochberg procedure will be applied to take into account multiplicity of testing (no adjustment for safety parameters). Adjustment for multiplicity will be done for the two treatment group comparisons (high dose vs placebo and low dose vs placebo) but not for the number of studied parameters.

Pharmacokinetic variables: Trough plasma levels of lanifibranor will be assessed at V1 (week 4) and V3 (week 24).

Study procedures:

For the patients with a liver biopsy performed within 6 months before screening, all diagnosis tests regarding the elimination of other causes of liver disease should have been

performed before the patient is considered for inclusion in the study as part of the routine medical care and will not be performed specifically for the study.

For the patients without a liver biopsy performed within 6 months before screening, the screening phase will be done in successive steps to avoid unnecessary biopsy:

Step 1: Review of the medical history, concomitant medications, physical examination, electrocardiogram, biological sampling and FibroScan (mandatory). Patients will be withdrawn from the study at this step in case of TE > 12.5 kPa (i.e. high probability of cirrhosis).

Step 2: MR/Liver MultiScan technology, only in selected sites. Patients will be withdrawn from the study at this step in case of LIF < 2 or MRI-PDFF ≤5%.

Step 3: A liver biopsy will be performed. In all cases, liver biopsy will be performed upon medical indication and according to the standard practice of the site.

After validation of the histological criteria by the central reading assessment, patients can be randomized.

All patients will undergo six visits, screening V-1 (-8 weeks to -2 weeks), randomization V0 (week 0), interim V1 (week 4), interim V2 (week 14), end-of-treatment V3 (week 24) and follow-up V4 (4 weeks after end-of-treatment). For all visits except for screening, time windows are defined up to +/- 3 days compared to V0 and are indicated in the Table 1 and Table 2. The screening visit time window is of 2 to 4 weeks before V0 when slides from a biopsy performed within the past 6 months are available, and of 2 to 8 weeks when the pre-treatment biopsy is part of the screening procedures.

Patients will be randomized to either dose of lanifibranor or placebo at V0 and treated for 24 weeks until V3.

A physical examination will be performed at each visit. An ECG will be done at screening, V1 and V3. Adverse events and concomitant medications will be recorded throughout the study.

A liver biopsy will be performed at V3.

The laboratory assessments performed at screening and end-of-treatment will include: haematology (CBC), blood biochemistry (ALT, AST, γGT, alkaline phosphatase, total bilirubin, conjugated bilirubin, ultra-sensitive CRP, total cholesterol, triglycerides, HDL cholesterol, LDL cholesterol, creatinine, fasting glucose, insulin, HgbA1c, albumin, CPK, sodium, potassium, calcium), and two serum bank samples.

Urine Samples will be collected at V0 *(including urine pregnancy test),* V2, V3 and V4. Urine analysis by dipstick evaluating hematuria will be done on site. Urine samples will then be stored for further assessment and/or for safety evaluation.

Trough plasma levels of lanifibranor will be assessed at V1 and V3.

Figure 1: NATIVE Study Design and Screening process

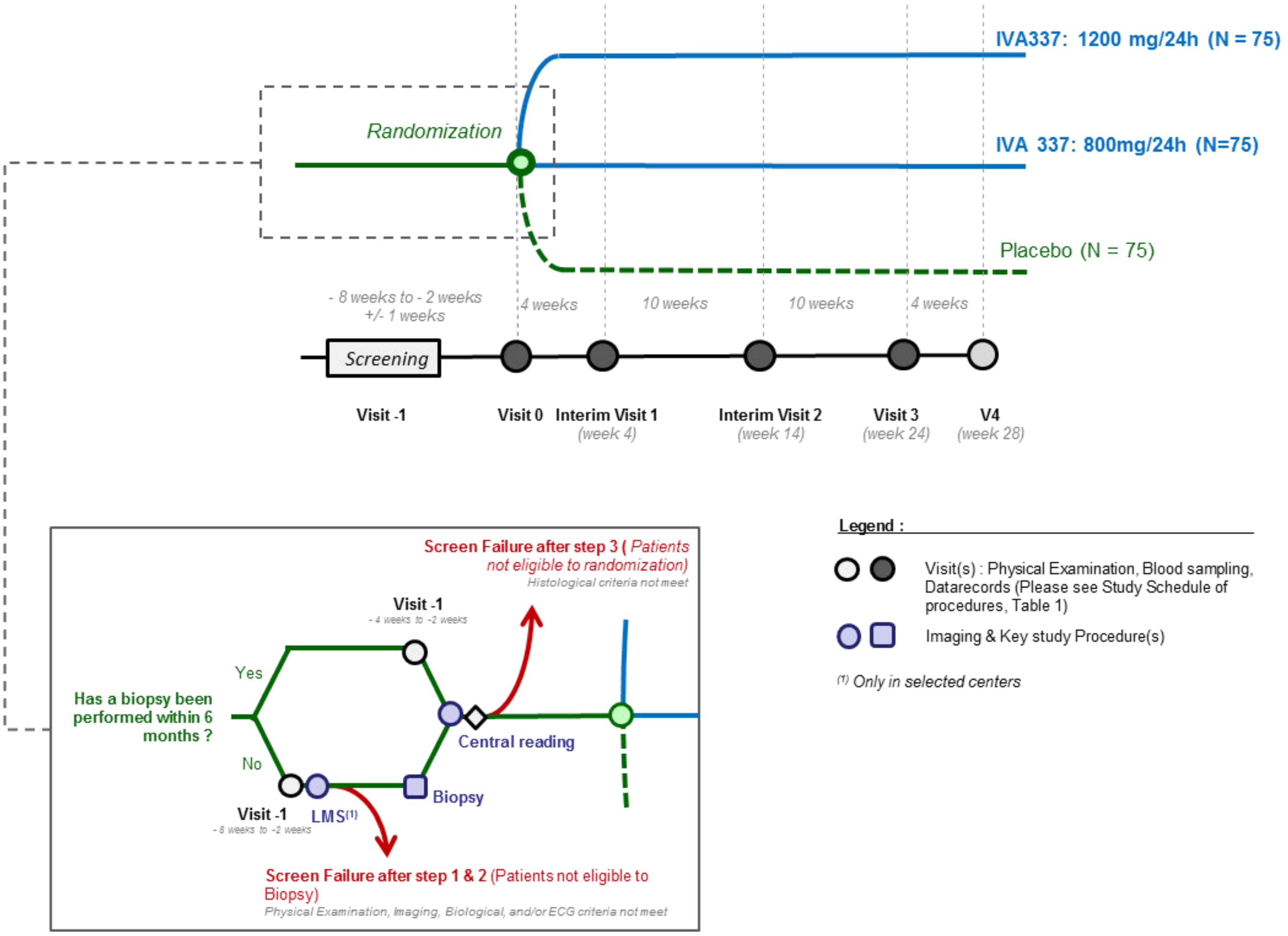

Table 1: Study Schedule of procedures (if a liver biopsy of less than 6 months is available before screening)

**Study Period**		**Screening**	**Treatment**				**Follow up**
**Visit**		**V-1**	**V0**	**V1**	**V2**	**V3**	**V4**
**Weeks (target+/-3days, referred to V0)**		**-4 to -2**	**0**	**4**	**14**	**24**	**28**
Informed consent		X					
**Inclusion/exclusion criteria**		X	X				
Demographics, Medical history		X					
Alcohol consumption		X	X	X	X	X	X
Physical examination/ vital signs:	*height (inclusion only), weight, waist circumference, SBP, DBP, HR*	X	X	X	X	X	X
12-lead Electrocardiogram		X		X		X	
Pregnancy test *((a): Blood test, βHCG; (b): Urine Test)*		X(a)	X(b)				
HIV serology		X					
Concomitant medications		X	X	X	X	X	X
Quality of life questionnaires			X			X	
Urine Samples			X		X	X	X
Blood Samples		X	X	X	X	X	X
FibroScan™ (if available)		X				X	
Central Reading of liver biopsy to confirm NASH diagnosis		X					
Liver biopsy at end of treatment						X	
**Primary efficacy evaluation: Inflammation and ballooning SAF**		X(c)				X	
**Secondary efficacy evaluation**							
NAS score and other liver histology indices		X				X	
Inflammatory markers	*fibrinogen, hs-CRP, alpha2-macroglobulin, haptoglobin levels*		X			X	
Glucose metabolism	*fasting glucose and in subjects with T2DM: HbA1c*	X	X	X	X	X	X
	Insulin, HOMA, Peptide C, Fructosamine		X			X	
Lipids	*Lipids: TC, HDL-C, calculated LDL-C and TG*	X	X	X	X	X	X
	FFA, adiponectin, Leptin and apoA1		X			X	
Chemistry	*Plasma Iron, Transferrin, Ferritin*	X				X	
Fibrosis markers	*TIMP-1, TIMP-2, Cytokeratin K18, hyaluronic acid, P3NP, FGF 21*		X			X	
**Exploratory criteria Biobank**							
Other biomarkers	*IL-6, IL-13, IL-17A, IL1B, TNF-a, INFg, APO B, APO C3, MMP2, MMP9, ProC3 (non-exhaustive list)*		X			X	
Genotype	*PNPLA3, TM6FS2*		X(d)				
**Safety assessments**							
Hematology	*WBC (+diff. Count), RBC, hemoglobin, MCV, hematocrit, Platelets, reticulocytes, CCMH, Hematocrit*	X	X	X	X	X	X
Inflammatory markers	*Nt-ProBNP*		X			X	
Chemistry	*creatinine, urea, albumin, CPK*	X	X	X	X	X	X
Liver tests	*AST, ALT, ALP, GGT, total bilirubin*	X	X	X	X	X	X
Bone	*B-Crosslaps, Osteocalcin*		X			X	
Coagulation factors	*INR*	X	X	X	X	X	X
Urine dipstick	*Hematuria*		X		X	X	X
**Pharmacokinetics:** IVA337 (+ metabolites) trough sampling				X		X	
**Adverse events**			X	X	X	X	X
**Dispense study treatment**			X	X	X		
**Compliance check**				X	X	X	

(c) Central reading on biopsy within the 6 months prior to screening to confirm NASH diagnosis;
(d) Only for patients having consent for genetic testing;

Table 2: Study Schedule of procedures (if a liver biopsy is NOT available before screening)

Study Period		Screening			Treatment				Follow up
**Visit**			V-1		V0	V1	V2	V3	V4
**Weeks (target +/- 3 days, referred to V0)**			-8 to -4		0	4	14	24	28
**Screening Step**		1	2	3					
Informed consent		X							
**Inclusion/exclusion criteria**		X			X				
Demographics, Medical history		X							
Alcohol consumption		X			X	X	X	X	X
Physical examination/ vital signs:	*height (inclusion only), weight, waist circumference, SBP, DBP, HR*	X			X	X	X	X	X
12-lead Electrocardiogram		X				X		X	
Pregnancy test *(Blood test, βHCG)*		X		X(a)					
Pregnancy test *(Urine Test)*					X				
HIV serology		X							
Concomitant medications		X			X	X	X	X	X
Quality of life questionnaires					X			X	
Urine Samples					X		X	X	X
Blood Samples		X		X(a)	X	X	X	X	X
LiverMultiScan/ Magnetic Resonance (in selected sites)			X(b)						
FibroScan™		X						X	
Liver biopsy				X(c)				X	
**Primary efficacy evaluation: Inflammation and ballooning SAF**				X				X	
**Secondary efficacy evaluation**									
NAS score and other liver histology indices				X				X	
Inflammatory markers	*fibrinogen, hs-CRP, alpha2-macroglobulin, haptoglobin levels*				X			X	
Glucose metabolism	*fasting glucose and in subjects with T2DM: HbA1c*	X		X(a)	X	X	X	X	X
	Insulin, HOMA, Peptide C, Fructosamine				X			X	
Lipids	*Lipids: TC, HDL-C, calculated LDL-C and TG*	X		X(a)	X	X	X	X	X
	FFA, adiponectin, Leptin and apoA1				X			X	
Chemistry	*Plasma Iron, Transferrin, Ferritin*	M			X			X	
Fibrosis markers	*TIMP-1, TIMP-2, Cytokeratin K18, hyaluronic acid, P3NP, FGF 21 (non-exhaustive list)*				X			X	
**Exploratory criteria Biobank**									
Other biomarkers	*IL-6, IL-13, IL-17A, IL1B, TNF-a, INFg, APO B, APO C3, MMP2, MMP9, ProC3 (non-exhaustive list)*				X			X	
Genotype	*PNPLA3, TM6FS2*				X(d)				
**Safety assessments**									
Hematology	*WBC (+diff. Count), RBC, hemoglobin, MCV, hematocrit, Platelets, reticulocytes, CCMH, Hematocrit*	X		X(a)	X	X	X	X	X
Inflammatory markers	*Nt-ProBNP*				X			X	
Chemistry	*creatinine, urea, albumin, CPK*	X		X(a)	X	X	X	X	X
Liver tests	*AST, ALT, ALP, GGT, total bilirubin*	X		X(a)	X	X	X	X	X
Bone	*B-Crosslaps, Osteocalcin*				X			X	
Coagulation factors	*INR*	X		X(a)	X	X	X	X	X
Urine dipstick	*Hematuria*				X		X	X	X
**Pharmacokinetics**: lanifibranor (+ metabolites) trough sampling						X		X	
**Adverse events**					X	X	X	X	X
**Dispense study treatment**					X	X	X		
**Compliance check**						X	X	X	

(a) to be done only if there are more than 4 weeks between V-1 and V0;
(b) to be done after checking inclusion / exclusion criteria, incl. lab tests , ECG and Fibroscan;
(c) to be done after checking the results of the Fibroscan and/or the Liver MultiScan/ Magnetic Resonance (in selected centers);
(d) Only for patients having consent for genetic testing;

Table of contents

REVISION HISTORY 2
SYNOPSIS 3
TABLE OF CONTENTS 13
LIST OF FIGURES 16
LIST OF TABLES 16
LIST OF ABBREVIATIONS AND DEFINITIONS OF TERMS 17
1. INTRODUCTION 21
1.1 Non Alcoholic Steato-Hepatitis (NASH) 21
1.2 Peroxisome Proliferator-Activated Receptor (PPAR) in NASH 21
1.3 Investigational product 23
1.4 Summary of lanifibranor Preclinical and clinical data 24
1.5 Rationale of the Study Design 28
2. INVESTIGATORS AND STUDY ADMINISTRATIVE STRUCTURE 31
2.1 Investigators and Other Participants 31
2.2 Sponsors Representatives 31
2.3 Pharmacovigilance and safety Institution 31
2.4 Other relevant Institutions 32
3. ETHICAL AND LEGAL CONSIDERATIONS 32
3.1 Institutional Review Board (IRB)/Independent Ethics Committee (IEC) 32
3.2 Protocol Amendments 33
3.3 Ethical Conduct of the Study 33
3.4 Good Clinical Pratice Responsibilities 33
3.5 Reporting of safety issues breaches of the protocol or ICH GCP 33
3.6 Insurance 34
3.7 Patient Information and Consent 34
3.8 Premature Termination 34
3.9 Definition of end of the trial 34
4. STUDY OBJECTIVES AND INVESTIGATIONAL PLAN 35
4.1 Study Design 35
4.2 Study Population 36
4.3 Study Medication 40
5. MEASUREMENTS ASSESSED AND STUDY FLOW CHART 42
5.1 Table of study procedures and assessments 42

5.2 Specific procedures for Assessment of outcomes 43
5.3 Procedures at each visit 46
6. SAFETY ASSESSMENT 49
6.1 Adverse Events 49
6.2 Serious Adverse Events or Reactions 50
6.3 Safety Variables 52
6.4 Data Safety Monitoring Committee 56
7. PHARMACOKINETIC ASSESSMENTS 57
7.1 IMP Concentration Measurements 57
7.2 Non-Compartimental Pharmacokinetic analysis 58
8. DATA QUALITY ASSURANCE 58
8.1 Monitoring 58
8.2 Data recording 59
8.3 Data management 59
8.4 Independent Audit 60
8.5 Regulatory Inspection 60
9. STATISTICAL METHODS 60
9.1 Efficacy Assessments 60
9.2 Sample Size 61
9.3 Randomization 62
9.4 Protocol Deviations 62
9.5 Data sets Analyzed 62
9.6 Demographic and Other Baseline Characteristics 63
9.7 Safety Analysis 63
10. PATIENT WITHDRAWAL & REPLACEMENT 67
10.1 Patient Withdrawal 67
10.2 Unblinding Process 68
11. TRAINING & INFORMATION 69
11.1 Training 69
11.2 Information of the Investigator 69
12. RECORDS & DATA 69
12.1 Source Records & Data 69
12.2 Case Report Forms 69
13. CONFIDENTIALITY 70

13.1 Confidentiality of Patient Data....70
13.2 Confidentiality of Sponsor Data....70
14. REPORTING & PUBLICATION....70
14.1 Study Report....70
14.2 Disclosure of Data and Publications....70
15. RECORD KEEPING....71
15.1 Study Site Records....71
15.2 Study Master File....71
BIBLIOGRAPHY....72
APPENDICES....78

List of Figures

FIGURE 1: NATIVE STUDY DESIGN AND SCREENING PROCESS 10

FIGURE 2: LANIFIBRANOR REDUCES INFLAMMATION AND STEATOSIS IN A 3 WEEKS MCD MODEL 25

FIGURE 3: PHASE IIA BIOMARKERS OF LANIFIBRANOR PPAR ACTIVATION IN DIABETIC PATIENTS 29

FIGURE 4: IMP PACKAGING 41

FIGURE 5: HISTOLOGICAL GRADING USED IN SAF SCORE. GRADING OF STEATOSIS. 90

List of Tables

TABLE 1: STUDY SCHEDULE OF PROCEDURES (IF A LIVER BIOPSY OF LESS THAN 6 MONTHS IS AVAILABLE BEFORE SCREENING)..11

TABLE 2: STUDY SCHEDULE OF PROCEDURES (IF A LIVER BIOPSY IS NOT AVAILABLE BEFORE SCREENING) 12

TABLE 3: INTERSPECIES COMPARISON OF HUMAN AND ANIMAL PLASMA EXPOSURE TO LANIFIBRANOR IN GENERAL TOXICITY STUDIES..26

TABLE 4 : INTERSPECIES COMPARISON OF HUMAN AND ANIMAL PLASMA EXPOSURE TO LANIFIBRANOR IN SEGMENT II REPRODUCTIVE TOXICITY STUDIES..27

TABLE 5: SUMMARY OF LABELLING DETAILS 42

TABLE 6 : BLOOD VOLUME TAKEN AT EACH VISIT PER TYPE...44

TABLE 7 : CENTRAL LABORATORY TESTS FOR SAFETY ASSESSMENT 53

TABLE 8 : CENTRAL LABORATORY TESTS FOR EFFICACY ASSESSMENT 54

TABLE 9 : BIOBANK TESTS...55

List of Abbreviations and Definitions of terms

ADR	Adverse drug reaction
AE	Adverse event
AHA	American Heart Association
ALP	Alkaline phosphatase
ALT	Alanine amino transferase
AST	Aspartate amino transferase
AUC	Area Under the Curve
AUC[0→24h]	Area under the plasma concentration curve from administration to 24h
AUC[0→t]	Area under the plasma concentration curve from administration to last observed concentration at time t
AUC[0→∞]	Area under the plasma concentration curve from administration to infinite time bpm beat per minute
BCS	Biopharmaceutics Classification System
BMI	Body mass index
CA	Competent Authority
CAP	Controlled Attenuation Parameter
CEC	Competent Ethics Committee
CBC	Complete blood count
CCL4	Chemokine (C-C motif) ligand 4
CDaa	Choline deficient l-amino acid
CL	Central Laboratory
Cmax	Maximal plasma concentration
CPK	Creatine phosphokinase
CRF	Case Report Forms
CRN	Clinical Research Network
CRP	C reactive protein
CV	Coefficient of variation
D	Day
DBP	Diastolic Blood Pressure
DNA	Deoxyribonucleic acid
DcSSc	Diffuse cutaneous systemic sclerosis
ECG	Electrocardiogram
ECM	Extra-Cellular Matrix
eCRF	Electronical Case Report Forms

eGFR	estimated Glomerular Filtration Rate
EGF	Epidermal Growth Factor
EMA	European Medicines Agency
FDA	Food and Drug Administration
FFS	Flinders Fatigue Scale
γGT	Gamma glutamyl transpeptidase
GCP	Good Clinical Practice
h	hour(s)
HBV	B virus hepatitis
HCV	C virus hepatitis
HDL-C	High-density lipoprotein cholesterol
HDPE	High-density polyethylene
HFD	High-fat diet
HOMA	Homeostasis model assessment of insulin resistance
HR	Heart rate
HSC	Hepatic stellate cells
hs-CRP	High-sensitivity C-reactive protein
ICH	International Conference on Harmonisation of Technical Requirements for Registration of Pharmaceuticals for human Use
IEC	Independant ethics committee
IFN	Interferon
IL	Interleukin
IMP	Investigational medicinal product
in	inch
INR	International normalized ratio
ITT	Intention to Treat
IWRS	Interactive Web Response System
KO mice	Knock-out mice
lbs	pound
LDL-C	Low density lipoprotein cholesterol
LDLR	Low-density lipoprotein receptor
LIF	Liver Inflammation and Fibrosis
LMS	LiverMultiScan
MCD	Methionine Choline deficient
MCHC	Mean corpuscular haemoglobin concentration

MCP	Monocyte Chemoattractant Protein
MCV	Mean corpuscular volume
MedDRA	Medical Dictionnary for Regulatory Affairs
MHC	Mean hemoglobin concentration
MTD	Maximum tolerated dose
MRI	Magnetic resonance imaging
MRI-PDFF	MRI proton density fat fraction
NAFLD	Non-alcoholic fatty liver disease
NAS	NAFLD Activity Score
NASH	Nonalcoholic steatohepatitis
NNT	Number Needed to Treat
NOAELs	No observed adverse effects levels
P3NP	N-terminal Pro-peptide of type III Procollagen
PBO	Placebo
PCR	Polymerase chain reaction
PDGF	Platelet-Derived Growth Factor
PK	Pharmacokinetic(s)
PP	Per Protocol
PPAR	Peroxisome Proliferator-Activated Receptor
PQ	PQ interval: duration in milliseconds from the beginning of P wave to onset of ventricular depolarization (Q and R)
Pro-C3	N-terminal Pro-peptide of type III Collagen
PTM	Placebo to match
PTT	Partial Thromboplastin Time
QD	Once a day (Quaque Die (Latin: Daily))
QRS	QRS interval: duration in milliseconds of the QRS-complex
QT	QT interval: duration in milliseconds from the beginning of Q wave to the end of T wave
QTc	Corrected QT interval
RBC	Red blood cell
RNA	Ribonucleic acid
SAE	Serious adverse event
SAF	Steatosis Activity Fibrosis
SAP	Statistical Analysis Plan
SBP	Systolic blood pressure

SD	Standard deviation
SF36	Short Form (36) Health Survey
SMA	α-Smooth Muscle Actin
SO	Safety officer
TBARS	Thiobarbituric Acid Reactive Substances
TE	Transient Elastography
TC	Total cholesterol
$t_{1/2}$	plasma concentration half life
tlag	Time delay between drug administration and the onset of drug absorption
T2DM	Type 2 Diabetes Mellitus
TE	Transient Elastography
TEAE	Treatment-emergent adverse event
TG	Triglycerides
TGF	Transforming growth factorTmax Time passed since administration at which the maximum plasma concentration occurs
TNF	Tumor Necrosis Factor
TSC	Trial Steering Committee
UUO	Unilateral Ureteral obstruction
ULN	upper limit of the normal range
V	Visit
VEGF	Vascular Epidermal Growth Factor
WBC	White blood cell
WMA	World Medical Association
WT mice	Wild type mice

1. Introduction

This is a phase IIb, randomized, double-blind, placebo-controlled, multicenter, dose-range, 24-week treatment study of IVA337 (lanifibranor) in adult subjects with nonalcoholic steatohepatitis (NASH).

This introductory section describes briefly the underlying conditions of the patients to be included in the study, the scientific rationale for exploring lanifibranor in NASH, summarizes the lanifibranor preclinical and clinical data and provides a rationale for the study design.

1.1 Non Alcoholic Steato-Hepatitis (NASH)

Non-alcoholic steatohepatitis is a liver disease characterized by fatty infiltration of the liver together with signs of inflammation and hepatocyte injury. Although pathophysiology is complex, its primary cause is still believed to be insulin resistance, as NASH occurs mostly in individuals that are overweight/obese or have type 2 diabetes (1). Because of the significant increase in the incidence of obesity and diabetes, NASH is rapidly becoming one of the major and most prevalent causes of liver diseases, ranking in the top 3 hepatopathies in different countries and ethnic backgrounds. This trend is expected to increase in the years to come.

The prognosis of NASH is determined both by progression of hepatic disease and by extrahepatic complications (2). Hepatic fibrosis progression is the main hepatic complication of NASH (3,4) and can culminate in the occurrence of cirrhosis with its major risks and complications (5,6). Extrahepatic complications include an increased risk of cardiovascular events and cardiovascular morbidity that seems to be independent of other known cardiometabolic risk factors (7–9). Several studies have now shown that patients with NASH have reduced survival compared to the age and sex-matched general population (10) and that they are exposed to the risk of end-stage liver disease (5,9), hepatocellular carcinoma (5,11) and increased liver-related mortality (9).

Treatment of NASH is a significant unmet clinical need. While diet and lifestyle changes are always to be recommended as first-line therapy and can be efficient in reducing NASH histological lesions, most patients fail to reach these objectives and compliance with these measures is usually poor (12,13). Therefore, targeted pharmacological therapies are usually necessary. It is now recognized that insulin resistance is the main mechanism leading to fat accumulation in the liver and insulin-sensitizing agents such as glitazones (14) and metformin (15) have been proposed as putative treatments of NASH. However, although some studies suggest a moderate efficacy of glitazones (the early-phase studies have shown that at least half of treated patients have no or a suboptimal response to glitazones (16) definitive proof of their efficacy is lacking and regarding a meta-analysis demonstrated no benefit (17)(18).

It is now recognized that development and progression of NASH is the result of a multi-step process (19). Insulin resistance promotes the transition from normal to fatty liver (20) while numerous additional factors ("hits") trigger the development of steatohepatitis in patients with steatosis.

1.2 Peroxisome Proliferator-Activated Receptor (PPAR) in NASH

1.2.1 *Rationale for PPAR in NASH*

The PPARs are ligand-activated transcription factors belonging to the nuclear hormone receptor family. A wide range of ligands can bind to the PPAR receptors resulting in a broad range of cellular signaling and biological processes. They are involved in development, reproduction,

inflammation, immune function, metabolism, apoptosis, growth and cancer (21,22). There are 3 PPARs isotypes, PPARα, PPARβ/δ and PPARγ. PPARα is highly expressed in the liver, especially in hepatocytes, and is implicated in the fatty acid transport, β-oxydation and the inflammatory response (23,24). The fibrates are synthetics ligands of PPARα and are used to treat dyslipidemia. PPARβ/δ is known to be involved in hepatic glucose utilization, lipoprotein metabolism, proliferation and to also have an anti-inflammatory activity within the liver (25–27). PPARγ is essentially expressed in the adipose tissue where it regulates inflammation, increases insulin sensitivity and promotes fatty acid uptake (28). Glitazones are the most described chemical family of PPARγ synthetic ligands. They have been developed for their antidiabetic and insulin sensitizing effect.

The synthetic ligands of PPARs, fibrates and glitazones were hence developed and tested for the treatment of dyslipidemia, to improve glucose homeostasis and for type 2 diabetes mellitus. NASH is considered as the hepatic manifestation of the metabolic syndrome, which is a set of events that could lead to diabetes, cardiovascular disease, and cerebrovascular accident (29). PPARs are known to be involved in several aspects of Non-alcoholic fatty liver disease (NAFLD) such as the hepatic triglyceride accumulation, which is the hallmark of this disease. Moreover, data suggest that PPARs are involved in the process of fibrogenesis (30). In hepatic fibrosis, PPARs, and especially PPARγ, have a role in preventing the activation of hepatic stellate cells, the key cells of the hepatic fibrotic process (30,31).

In patients, pioglitazone, a PPARγ agonist, at the dose of 30 mg/day for 96 weeks, significantly reduced alanine aminotransferase (ALT) and aspartate aminotransferase (AST), as well as hepatic steatosis and lobular inflammation compared to placebo (32). In another study, at the dose of 45 mg/day for 24 weeks and in association with a 500 Kcal reduction diet, pioglitazone significantly decreased ALT and AST and was associated with a significant improvement of steatosis, ballooning necrosis and inflammation (14). Furthermore, PPARα expression negatively correlates with the severity of NASH (33).

Taken together the beneficial effects of PPARs in NAFLD, inflammation and in fibrosis suggest them as a promising candidate for the treatment of NASH

1.2.2 *PPARs in preclinical NASH model*

There is currently no experimental model replicating all the different aspect of human NASH. However, several models exist that reproduce part of, or even most of, the human pathology features. Among these models, the most frequently used is the Methionine Choline deficient (MCD) diet. The effects of several PPARs agonists have been tested in this model.

It had been demonstrated that PPARα knock-out mice (KO Mice) are more sensitive to develop steatohepatitis under MCD diet than wild type mice (WT mice) (34). In the same way, treatment with Wy-14643, a PPARα agonist, in a prophylactic or curative mode could prevent or reverse MCD-induced steatohepatitis (34,35). Moreover Wy-14643 could also reduce the hepatic fibrosis induced by MCD diet (35). In a model of APOE2 KO mice (fed with western high fat diet leading to steatosis and inflammation), the administration of fenofibrate prevents steatosis and expression of inflammatory markers (36).

A positive effect of PPARβ/δ has been demonstrated in the MCD diet in mice where GW501516 prevents steatosis inflammation (illustrated by a decrease in Ribonucleic acid (RNA) expression of TGF-β1, IL-6, IL-1β, MCP-1, TNF-α) and Hepatic Stellate Cells (HSC) activation (37). Moreover an exacerbated phenotype of Chemokine (C-C motif) ligand 4 (CCL4–induced) hepatotoxicity was observed in PPARβ/δ null mice including increase in serum ALT and increase in mRNA related to fibrosis such as α-Smooth Muscle Actin (α-SMA) (38).

The beneficial effect of PPARγ in NASH was demonstrated in the MCD diet model by overexpressing PPARγ by the administration of adenovirus carrying PPARγ. Mice overexpressing PPARγ demonstrated a prevention of steatosis, inflammation and fibrosis (39–41). The effect was stronger with the addition of rosiglitazone, a selective PPARγ agonist. On the contrary, mice deficient for PPARγ and treated with rosiglitazone or mice overexpressing PPARγ and treated with GW9662 (a PPARγ antagonist) presented a more severe steatohepatitis (39,40). Similarly to these studies, the administration of rosiglitazone prevented steatohepatitis and fibrosis in a model of middle-aged male low-density lipoprotein receptor (LDLR)(-/-) mice fed a high-fat diet (HFD)(42). The beneficial effect of PPARγ was also studied using pioglitazone, another PPARγ agonist. Pioglitazone demonstrated beneficial effects on steatohepatitis and a slight effect on fibrosis in a choline deficient L-amino acid defined diet (CDAA) rat model of NASH (43). In a model of HFD mice or rat and in a model of MCD mice, pioglitazone demonstrated beneficial effects on steatosis (44–46) as well as on insulin resistance. Pioglitazone has also been tested in a CCL4-induced liver fibrosis model where its effect is limited because it is dependent on the duration and severity of the disease (43). However, it is of importance to notice that it has been demonstrated by several publications that PPARγ plays a role in the fate of HSC cells. Indeed, a decreased expression of PPARγ in HSC is observed during the transdifferentiation from a quiescent state to an activated and proliferative state(47–50). PPARγ, PPARδ ligands (like the dual PPARα/δ GFT505 and KD3010) and PPARα, all protect to various extent, from CCL4-induced, MCD diet-induced and bile duct ligation-induced fibrosis (51,52).

1.3 Investigational product

IVA337, lanifibranor, a new chemical entity, (4-[1-(1,3-benzothiazol-6-ylsulfonyl)-5-chloro-1H-indol-2-yl]butanoic acid) is an almost white to light brown colored solid.

Structural formula, including relative and absolute stereochemistry:

Molecular Formula:	C19H15Cl N2O4S2
Molecular weight:	434.92
Chirality:	No asymmetric carbon
Polymorphism:	β form crystal

The Investigational Medicinal Product is a white to off-white bi-convex tablet, weighing approximately 927 mg and showing a breakline to facilitate the administration to patients. The film-coated tablet contains 400 mg of the active ingredient lanifibranor (IVA337) for an immediate release formulation.

Film-coated tablets with a core containing 900 mg of a physical mixture of lactose monohydrate, microcrystalline cellulose, pre-gelatinised starch and magnesium stearate serve as placebo.

The Investigational Medicinal Products used in this clinical phase IIb trial will be packaged in containers of High Density Polyethylene (HDPE) with proper HDPE closures fitted with a silica cartridge.

Based on the results of the ongoing stability studies, a shelf life of 2 years (storage at 25°C or below) is proposed for the Investigational Medicinal Product provided it is stored in the original package, the HDPE bottles.

Furthermore, the stability data at 40°C/75%RH demonstrate that temperature excursions (e.g. during shipment, storage) will not significantly affect the stability of lanifibranor 400 mg film-coated tablets.

To avoid the potential moisture ingress, which may happen during the use of the monthly pack, a twist-off cap integrated with a 2 g of silica gel has been selected for the closure. The data of the "in use test" 35 days at 25°C/60%RH open bottles have confirmed the stability of the Investigational Medicinal Product during its proposed clinical use.

1.4 Summary of lanifibranor Preclinical and clinical data

1.4.1 *Pharmacology*

NASH is a multifactorial and multi-step disease. A first component of this pathology includes the metabolic syndrome related to insulin resistance, triglyceride accumulation, obesity etc. A second component is the fibrosis. In our pre-clinical studies, we demonstrated the beneficial effect of lanifibranor in several component of the metabolic syndrome as well as in fibrosis.

Lanifibranor, in previous experiments, was shown to exert an anti-diabetic and antihyperlipidemic activity in db/db mice, ZDF rats, diet-induced obese mice and the WOKW rat model of metabolic syndrome. Furthermore, it increased serum apoA1 level in human apoA1 transgenic mice. In functional cellular tests, lanifibranor increased fatty acid β-oxidation, stimulated cholesterol efflux, induced the expression of aP2 and adiponectin genes as well as ABCA1, ABCG1 and LXRα genes, while it reduced MCP-1 secretion. All these effects are signature of the activation of the 3 PPAR receptor isoforms.

Recently, it was demonstrated *in vivo* that lanifibranor displays an antifibrotic activity in bleomycin-induced lung fibrosis, in bleomycin-induced skin fibrosis (in preventive and curative mode), in a model of unilateral ureteral obstruction (UUO)-induced kidney fibrosis and in CCL4-induced fibrosis in the liver. In the liver, lanifibranor is active in both preventive as well as curative mode, thus providing evidence for an anti-fibrotic effect in established fibrotic disease. In the liver it was demonstrated that lanifibranor at 15 and 30 mg/kg was able to inhibit in a dose dependent manner the collagen deposition within the liver (-42% and -73.16% respectively). Lanifibranor was also able to decrease the serum triglycerides and ALT.

HSC are the cells responsible for the secretion the Extra-Cellular Matrix (ECM) and especially the collagen when activated (myofibroblast transdifferenciation of HSC). The main cellular process underwent by the HSC in a fibrogenenic environment is proliferation and activation. The proliferation assay is based on PDGF and the activation is based on stiffness (stiffness of the plated plastic). PDGF is secreted by a variety of cell types and plays a central role in fibrogenesis. It induces HSC proliferation and migration, thus contributing to the increase of the number of matrix-secreting cells. During fibrosis, the growing deposition of ECM increases the stiffness of the liver and participates to myofibroblast transdifferentiation of HSC. Lanifibranor was able to fully inhibit the proliferation induced by PDGF and was able to prevent the overexpression of α-SMA and the hallmark of myofibroblast, induced by stiffness. These findings strongly support the anti-fibrotic action of lanifibranor in the target cells.

Activity of lanifibranor on liver steatosis and inflammation, two main components of NASH, was evaluated in a 3-week model of MCD diet in C57bl/6 mice (report in preparation). It was shown that lanifibranor prevented steatosis in a dose-dependent manner at the doses of 10 and 30 mg/kg (-88.38% and -97.89% respectively compared to vehicle, see Figure 2). Lanifibranor also prevented inflammation at the doses of 10 and 30 mg/kg (-44.03% and -74.49% respectively compared to vehicle). Lanifibranor also prevented the increase in ALT transaminase induced by MCD diet (-49.35% and 67.26% at 10 and 30 mg/kg respectively), demonstrating hepatoprotective effect.

Figure 2: Lanifibranor Reduces Inflammation and Steatosis in a 3 Weeks MCD Model

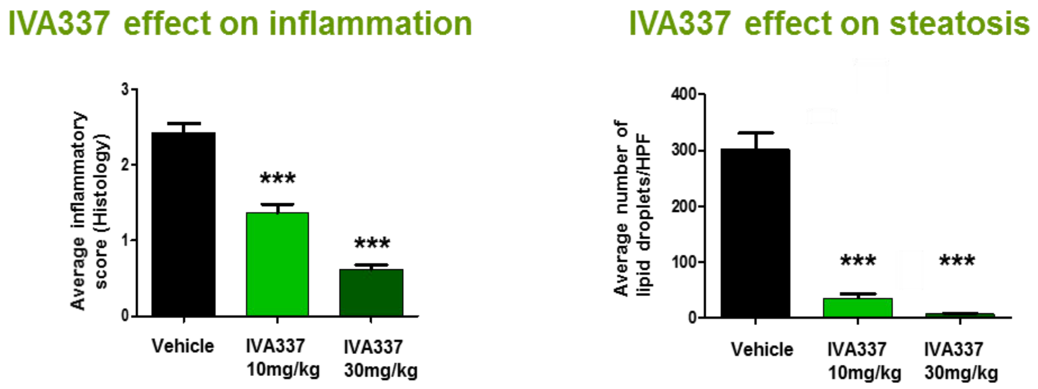

1.4.2 *Pharmacokinetics*

Lanifibranor is considered as a class II compound as regard to the Biopharmaceutics Classification System (BCS) Guidance: high permeability and low solubility. It was evidenced in all tested species that lanifibranor absorption is dependent on the drug substance form and formulation, leading to a bioavailability in animals from 15% up to >80%. An enterohepatic recycling was evidenced, and a food effect was observed in human in the presence of high fat breakfast.

After oral administration in human, maximum plasma levels (Cmax) of lanifibranor were reached at about 1.0-3.5 h, and terminal plasma elimination half-life was around 10-15 h. Both Area Under the Curve (AUC) and Cmax increased in a linear but slightly less than dose-proportional manner. Steady-state lanifibranor concentrations were generally achieved by Day 3 after daily dose administration, with no significant accumulation.

The mean apparent volume of distribution Vz/F ranged from 318 to 607 L in healthy subjects. Lanifibranor was extensively distributed in monkeys (~10 L/kg). IVA337 binds very highly to plasma proteins in all tested species (~99.9%) and no affinity to blood cells was observed. By autoradiography in rat, lanifibranor was shown to be mainly distributed in the excretory organs (liver, kidneys, lungs, gastrointestinal tract) and in placenta in pregnant rats with no affinity for the melanin tissues and with very limited central nervous system penetration.

Lanifibranor-related radioactivity elimination was mainly faecal (>85%) and the urinary excretion was minor (<7%) with a very low renal clearance. The excretion was essentially complete after 3 days.

AUC indicated approximately 23% of the radioactivity in plasma were related to lanifibranor, while 77% were related to its metabolites. Lanifibranor was observed *in vitro* to be metabolized either by enzymes of phase I (mainly via CYP isoforms CYP2C8, 2C19 and 3A4) and phase II (mainly via UGT 2B7, 1A1 and 1A3). No affinity of lanifibranor to uptake or efflux transporters as substrate was evidenced *in vitro*.

Among metabolites identified in plasma, faeces and urine, the main lanifibranor metabolites observed in human were the acyl glucuronide (IV1197736, with similar exposure to that of the parent compound), a monohydroxylated (IV1537661) and the benzothiazole ring opened lanifibranor derivative (IV1197347). All other metabolites were reported to represent less than 5% of the administered dose (14C-ADME study in human). The completion of the quantitative comparison of the animal versus human plasma exposure to these three metabolites is ongoing.

Lanifibranor was shown to be an inhibitor of CYP2C8 (IC_{50} 4µM, Ki 8µM), CYP2C9 (IC_{50} 8µM, Ki 21µM), CYP2C19 (IC_{50} 25µM) and CYP2B6 (IC_{50} 8µM). A limited risk of CYP induction could be expected *in vivo*, as only a slight CYP3A4 induction was observed at 20 µM of lanifibranor in one among all tested donors. No inhibition of xanthine and aldehyde oxydase inhibition by lanifibranor was evidenced. Lanifibranor directly inhibited UGT1A1, UGT1A3, UGT1A4, UGT1A6, UGT1A9, UGT2B7, UGT2B15 and UGT2B17 with IC_{50} values of 13, 26 to 38, 20, 83, 42, 33, 23 and 22 µM, respectively. Lanifibranor was shown to inhibit uptake and efflux transporters but with relatively high IC_{50} values: MDR1 (8.5 µM), BCRP (15.2 µM), BSEP (16.8 µM), OCT2 (267 µM), except for OATP1B1/3 (~1 µM). No significant drug-drug interaction was observed with Simvastatin (<2-fold), and no PK/genotype (CYP450 isoenzymes and UGTs isoform) relationship has been identified. There were a 22% increase in Cmax and 39% increase in AUC of lanifibranor in elderly subjects as compared to young subjects.

1.4.3 *Toxicology*

Lanifibranor has a very low acute toxicity (maximal non-lethal dose of 2000 mg/kg in rodents) and is not irritating to the skin.

Lanifibranor is devoid of genotoxic potential in the full international conference on harmonization (ICH) battery of genotoxicity assays. The three major human metabolites, IV1197736, IV1197347 and IV1537661, were also found devoid of genotoxic potential in a bacterial reverse mutation test and a mammalian cell assay.

Lanifibranor is devoid of deleterious effects on vital functions such as cardiovascular, central and autonomic nervous system and respiratory system. In a 9-week head-to-head comparative study in rats aimed at evaluating side effects of PPAR agonists such as plasma volume expansion and secondary cardiac hypertrophy, or skeletal muscle toxicity, daily doses of lanifibranor up to 1000 mg/kg compared favorably with other PPAR agonists rosiglitazone, muraglitazar and tesaglitazar. These observations were substantiated in repeated dose toxicity studies with lanifibranor in rats and monkeys.

No potential risks have been identified from pivotal toxicology studies with lanifibranor in rat and in monkey and segment II reproductive toxicity studies in rats and rabbits. All treatment-related findings (i.e. liver, bone marrow, adipose tissue) were reversible.

Table 3: Interspecies Comparison of Human and Animal Plasma Exposure to Lanifibranor in General Toxicity Studies

Human Plasma AUC at Estimated Active Dose (µg.h/mL)	Species	NOAEL Male-Female (mg/kg/d)	AUC at NOAEL Male-Female (µg h/mL)	Safety Margin
	Mouse*	25	225 - 278	~5
50	Rat**	2000 - 1000	798 - 1144	16 - 23
	Monkey##	1000	110 - 82	1.6 - 2.2

*: from 13-week toxicology study in CD-1 mice
**: from 13-week toxicology study in Wistar rats
##: from 26-week toxicology study in Cynomolgus monkeys

Table 4 : Interspecies Comparison of Human and Animal Plasma Exposure to Lanifibranor in Segment II Reproductive Toxicity Studies

Species/Strain	NOAEL (mg/kg/d)	Plasma Exposure (AUC0-24 as µg h/mL) Gender	lanifibranor
Han Wistar Rat	100	Female	437 (8.7)1
New Zealand White Rabbit	3	Female	105 (2.1)

[1] values in brackets show exposure margin factor to clinical exposure

The safety margins over expected human exposure in general toxicology studies were at least 20-fold in rats and 2-fold in monkeys (Table 3). The safety margins over expected human exposure established in segment II reproductive toxicity studies were 9-fold in rats and 2-fold in rabbits (Table 4). A preliminary 2-week pharmacokinetic and toxicity study in the mouse, where plasma exposures were markedly higher than those achieved in volunteers given the highest tested dose of 1500 mg/day for two weeks (approximately 40-fold) and higher than those achieved in the chronic rat study, has evidenced signs of toxicity for the kidney, the heart and the skeletal muscles, in particular myositis of skeletal muscles, minimal to moderate myocarditis and tubulo-interstitial nephritis. These organs are known targets for toxicity of PPAR agonists. However, a recently conducted 13-week study in mice established an adequate 5-fold safety margin over expected human exposure at a NOAEL (no observed adverse effects levels) of 25 mg/kg/day.

1.4.4 *Previous Lanifibranor Clinical Data*

Lanifibranor has previously been in clinical development up to Phase II for the treatment of type 2 diabetes. The development for diabetes was discontinued for strategic reasons.

Three clinical studies have been conducted in healthy subjects, one study in patients with type 2 diabetes, and one phase II study is ongoing in the treatment of diffuse cutaneous systemic sclerosis.

The objectives of study S337.1.001 were to assess pharmacokinetics, pharmacodynamics, safety and tolerability of lanifibranor after single increasing doses and after multiple increasing doses.

The objectives of study S337.1.002 were to study the effect of lanifibranor on the pharmacokinetics of simvastatin and simvastatin acid and to evaluate the pharmacokinetics of the two crystalline anhydrous solid alpha and beta forms of lanifibranor.

The objectives of study S337.1.005 were to assess the relative bioavailability of lanifibranor oral suspension and capsules (cross-over design), and to assess, *in vivo* in human, the completeness of excretion, routes and rates of elimination of radioactivity, the metabolic pathways, and identity of lanifibranor metabolites after single administration of 14C-IVA337.

The objectives of study S337.2.001 were to evaluate the safety, tolerability, pharmacokinetics and pharmacodynamics of lanifibranor in doses of 400 to 1200 mg daily over a 4-week period in patients with type 2 diabetes.

The ongoing study IVA_01_337_HSSC_15_001 is a randomized, double-blind, placebo-controlled, multicenter proof-of-concept trial of lanifibranor in the treatment of diffuse cutaneous systemic sclerosis (DcSSc).

In clinical trials performed in healthy volunteers and patients with type 2 diabetes, lanifibranor was safe and well tolerated. Treatments included single doses of 25 mg to 1000 mg, multiple doses of 150 mg to 1500 mg once daily for 14 consecutive days, and once daily doses of 400, 800, and

1400 mg for 4 weeks. The reported adverse events considered to have a causal relationship to the study drug included dizziness, postural dizziness, headache, hot flush, lethargy, somnolence, constipation, trends towards decreases in red blood cell count, haemoglobin and haematocrit in healthy volunteers, and hypochromic anaemia, constipation, urinary tract infection and headache in patients with type 2 diabetes. There were no clinically relevant time or dose-related changes on cardiac and muscle markers, liver enzymes or markers of renal function. The 12-lead electrocardiogram (ECG) did not show clinically significant changes.

1.5 Rationale of the Study Design

1.5.1 *General Considerations*

This study is a Phase IIb randomized (stratified on diabetes), double-blind, placebo-controlled, multicenter, dose-range, 24-week treatment study of lanifibranor in adult subjects with nonalcoholic steatohepatitis (NASH).

1.5.2 *Rationale for the Choice of Dose and Duration of Repeated Administration*

The evaluated treatment will be as tablets of lanifibranor 400 mg, two doses of lanifibranor (800 and 1200 mg once a day (QD)) versus placebo, i.e. three tablets (lanifibranor or indistinguishable placebo) per os, once daily, for 24 weeks. To improve bioavailability, drug intake is combined with food.

Doses have been selected based on the available biomarker (adiponectin, HDL cholesterol, triglycerides) results in diabetes type 2 patients (Phase IIa study):

- 800 mg per day: lowest and statistically significant active dose on the markers of the three PPAR α, δ and γ activity
- 1200 mg per day: dosage showing consistent activity on the markers of PPAR α, δ and γ activity, being well tolerated

Pharmacodynamic biomarkers of PPARs activation in diabetic patients (Phase IIa study) are shown in Figure 3.

Clinical, biological and ECG safety in healthy volunteers and diabetic patients has been shown to be good up to the top dose used in this trial or at doses that are higher. There is no toxicology target organ of major concern for the clinic.

Placebo is chosen as the comparator, as there is no licensed treatment for NASH.

Treatment duration has been set to 6 months. This is a phase IIb study and the aim to assess safety and efficacy in order to progress to a phase III. Improvement in ALT, AST, γGT as well as insulin resistance can be observed within a couple of months of treatment, as has been observed in several trials seen with other PPARs agonists (14,53), suggesting that treatment efficacy can be observed early in the course of treatment. Furthermore, histological changes, especially in necroinflammation, have been observed after a 6-month treatment with a PPARγ agonist (14). Therefore, taking into account the postulated end points, a treatment of 6 months in a proof of concept study is sufficient to assess study drug efficacy and will furthermore also limit the time on placebo for one third of the study patients.

The study only imposes 6 visits including the screen and follow-up visits and, apart from the liver biopsy at screening if no historical biopsy is available within 6 months of the screening visit, and at the end of the treatment and periodic blood sampling, there are no other invasive procedures. There is a very limited number of prohibited medications that should not interfere with standard

medical care of the patients. For obvious reasons, fibrates, as they are PPARα ligands, are not allowed, but dyslipidaemias can be treated with statins, which are allowed.

Figure 3: Phase IIa biomarkers of Lanifibranor PPAR activation in diabetic patients

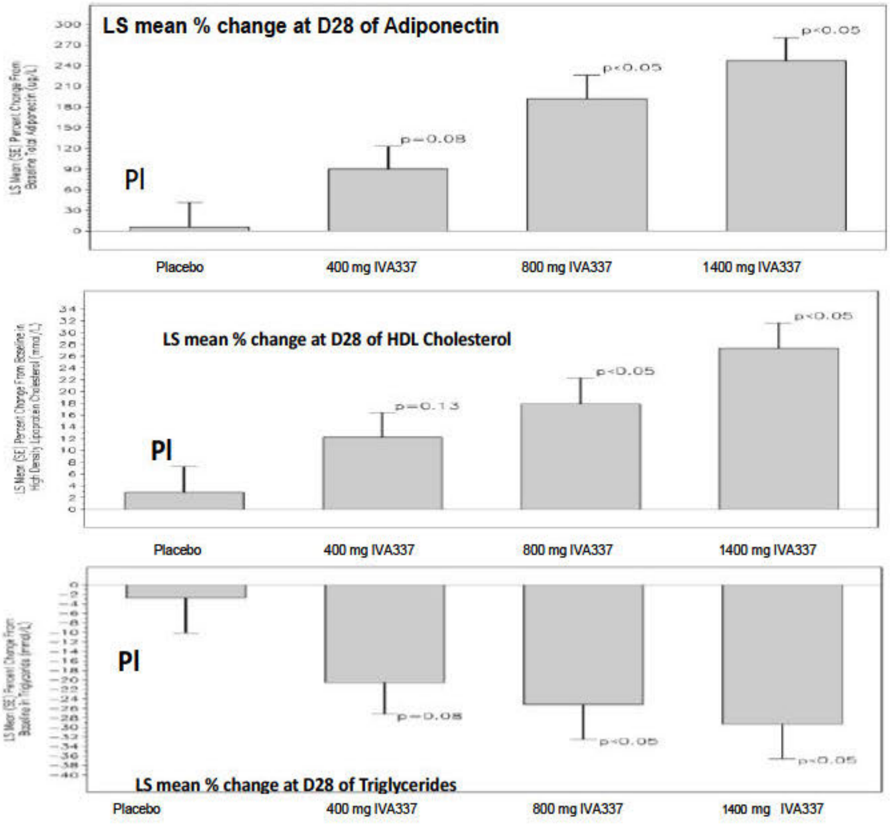

Two thirds of the patient are on verum treatment and can expect an improvement of their NASH and metabolic syndrome based on the results of previous clinical studies with PPARα, γ and α/δ agonists. The two doses chosen for the trial demonstrated significant improvement in biomarkers of PPAR α, γ and probably δ in diabetic patients.

As there is still no non-invasive tool sufficiently validated to accurately assess both the diagnosis as well as the treatment response in NASH, the only reliable tool to assess treatment efficacy is liver histology. As the primary endpoint is related to the reduction of necroinflammatory changes, a liver biopsy is indispensable to assess treatment efficacy and is therefore requested at the end of treatment. This is in line with the most recent recommendations on clinical trials in NASH (54,55). This is also in line with the design of the large Phase III trials that are currently ongoing (www.clinicaltrials.gov) and in which multiple biopsies are required for treatment efficacy assessment in large numbers of patients.

The study will require the usual specific informed consent form approved at each site to be signed by patients. The study does not impose any major change in the standard practice of liver biopsy at the site (see Appendix C). In a large (N=2084 biopsies performed for heterogeneous indications, mainly hepatitis C) nationwide French study published 15 years ago (56), no death was observed and the incidence of major complication was 0.57% (12 patients, vasovagal episode: 4, hemoperitoneum: 1 patient with cirrhosis, biliary peritonitis: 3, pneumothorax: 1, puncture of other viscera: 3). These data have been confirmed with more recent publications, except that the introduction of ultra-sound guidance decreased dramatically the risk of perforation of other organs

(57–61). The benefit/risk ratio of participation in the study is acceptable, i.e. out-patient procedure, in patients with controlled hemostatic parameters, no cirrhosis, etc. and other studies have been conducted with the same design.

The following section discusses the main histologic assessment criteria.

1.5.3 *Justification for using the Steatosis Activity Fibrosis (SAF) histological score*

Although there has been some evolution in the diagnostic criteria defining NASH, there is currently a large consensus, endorsed by renowned hepatologists and liver pathologists, and widely accepted in the liver community (62) that the diagnosis of NASH requires the combination of all three of the following histological features: steatosis, ballooning/clarification of hepatocytes, and lobular inflammation (Appendix C).

While steatosis is considered to be a relatively benign lesion, ballooning and inflammation define the so-called "activity" of NASH. Activity is very likely driving fibrogenesis, which is the main histological long term prognostic factor for liver-related and also non-liver-related mortality associated with NASH. Reducing activity to prevent fibrogenesis is the treatment goal evaluated in this clinical trial.

At present, no non-invasive markers have been shown to be accurate enough to diagnose NASH or to assess treatment effect. More specifically, no non-invasive markers have been validated to assess the different components of the diagnosis of NASH or to assess the evolution of these different components upon treatment. Only for steatosis, a reliable non-invasive technique, namely MR spectroscopy, has been sufficiently validated. Steatosis is, however, only one component of the definition of NASH, and, as outlined before, the other aspects of the disease, especially activity and fibrosis, are pivotal in terms of prognosis and treatment efficacy. Liver biopsy, although an invasive procedure, hence remains the gold standard to establish NASH diagnosis and there is no surrogate marker available to assess the different features of NASH that constitute its definition or that allow to reliably assess treatment efficacy as it is defined in the present trial, as well as in other Phase II trials. Also in the current Food and Drug Administration (FDA) and European Medicines Agency (EMA) approved Phase III trials, treatment efficacy is defined based on histological criteria and consequently liver biopsy is a prerequisite to accurately evaluate both the baseline liver status of the patient as well as the efficacy of the treatment after a predefined treatment period.

The activity sub score of the SAF score has been chosen as the main assessment criteria (63).The SAF has been developed by the European FLIP Pathology Consortium (64).

This scoring system assesses semi-quantitatively and separately Steatosis, Activity and Fibrosis as described in detail in the following section.

The Activity score of SAF is the addition of hepatocyte ballooning, the hallmark of NASH, and inflammation (both assessed from 0 to 2). By giving an equal weight to both lesion and not including steatosis into this score, a grade of activity $\geq$ 2 ensures to get at least 95% of NASH (63). Using Activity score A $\geq$3 ensures also that only the most severe NASH, which are those prone to develop fibrosis, will be selected.

This selection of NASH cases with severe activity cannot be done as accurately by using the NAS score (the NAFLD Activity Score that is part of the NASH Clinical Research Network (NASH CRN) Scoring System) (65), a composite score that is the unweighted sum of the score of steatosis (from 0 to 3), ballooning (from 0 to 2) and inflammation (from 0 to 3). Since this score includes steatosis, which is supposed to be a benign lesion, and underweight ballooning (graded from 0 to 2) compared to steatosis and inflammation (each being graded from 0 to 3), this score does not reflect activity of the disease as accurately as does the activity score of SAF.

Finally, the criteria chosen for the definition of ballooning in the SAF score have been shown to be highly reproducible, which guarantees also the robustness of these histological criteria (64).

2. Investigators and Study Administrative Structure

2.1 Investigators and Other Participants

The study will be performed in at least 60 sites across Europe, United States of America (USA) and other countries worldwide (incl. Canada, Australia and Republic of Mauritius).

Principal Investigators: **Pr. Sven Francque**, MD, PhD, Division of Gastroenterology and Hepatology, Antwerp University Hospital, Wilrijkstraat 10, B-2650 Edegem, Belgium; Pr. **Manal F. Abdelmalek**, MD, MPH, Division of Gastroenterology / Hepatology, Duke University, 40 Duke Medicine Circle, Clinic 2H / 2J, Durham, NC 27710, USA.

2.2 Sponsors Representatives

Sponsor: Inventiva S.A,
50 rue de DIJON, 21121 Daix, France
Tel: +33 (0) 380 447 631
Fax: +33 (0) 380 447 561

Chief Medical Officer (CMO): [redacted]

Person authorized to sign protocol on behalf of the Sponsor: [redacted]

Clinical Research Physician: [redacted]

The conduct of the study is delegated to the monitoring institution [redacted] except for Bulgaria, where the conduct of the study is delegated to [redacted], for Republic of Mauritius and La Réunion island, where the conduct of the study is delegated to [redacted], for USA, where the conduct of the study is delegated to [redacted] and for Slovenia and 2 sites in Poland, where the conduct of the study is delegated to [redacted]

The overall implementation and follow-up of computerised systems (IWRS, eCRF), the Data-management and the statistical analysis are delegated to [redacted].

2.3 Pharmacovigilance and safety Institution

The Safety of the study is delegated to the pharmacovigilance provider solutions:

[redacted]

2.4 Other relevant Institutions

Central laboratory, Clinical biology and biomarker assays:

[redacted]

[redacted]

Product Manufacturing:

[redacted]

Clinical Packaging:

[redacted]

Pharmacokinetics Assay Laboratory:

[redacted]

[redacted]

Cardiac Safety, ECG Central Read:

[redacted]

Histology and Central Liver Biopsy Reading:

[redacted]

3. Ethical and Legal Considerations

It is the responsibility of Inventiva to have the prospective approval of the study from the Competant authority (CA) and the Institutional Review Board (IRB)/Independent Ethics Committee (IEC). The IRB/EC and CA receive annual safety and interim reports and are informed about study stop/end in agreement with local requirements.

3.1 Institutional Review Board (IRB)/Independent Ethics Committee (IEC)

The Sponsor must submit this protocol to the appropriate IRB/IEC, and is required to forward to the Investigator a copy of the written and dated approval/favorable signed opinion.

The study (study number, Protocol title and version number), the documents reviewed (protocol, Informed Consent Form, Investigator's brochure, etc.) and the date of the review should be clearly stated on the written IRB/IEC approval/favorable opinion. All correspondence with the IRB/IEC should be retained in the Investigator File.

Investigational Product will not be released at the study site and the trial will not start until a copy of written and dated approval favorable opinion has been received by the Sponsor.

3.2 Protocol Amendments

Any change to this protocol by the Investigator or the Sponsor must be discussed and agreed.

The change, presented as an amendment in written form to the protocol, will be signed by the different parties (Investigator, Sponsor) and submitted to the IRB/IEC and whenever applicable to the CA for their approval.

Following approval, the amendment will be sent to all participating Investigators. The amendment cannot be acted upon prior to the outcome of this decision.

Amendment regarding minor modifications (administrative modifications) will be submitted to the IRB/IEC for information purposes only and whenever applicable to CA.

The only circumstance in which an amendment may be initiated prior to IRB/IEC and CA approval is where the change is necessary to eliminate apparent immediate hazards to the patients. In that event, the Investigator must notify the IRB/IEC and Inventiva in writing immediately after the implementation. All updates to the Investigator's Brochure, Protocol or any amedement-impacted documents will be sent to the IRB/IEC and CA whenever applicable.

If requested, a progress report could be sent to the IRB/IEC annually and a summary of the trial's outcome could also be provided at the end of the clinical trial.

3.3 Ethical Conduct of the Study

This protocol complies with the principles laid down by the 59th World Medical Assembly (Fortaleza 2013, Appendix A: World medical association declaration of Helsinki) and all applicable amendments laid down by the World Medical Assemblies, the guidelines of Good Clinical Practice CPMP/ICH/135/95, the European and applicable regulations per country, and any other relevant local requirement and laws.

3.4 Good Clinical Pratice Responsibilities

The responsibilities of the Sponsor and the Investigator will be as defined in the ICH GCP guidelines and applicable regulatory requirements. The Investigator is responsible for adhering to the responsibilities of Investigators, for dispensing the IMP in accordance with the final protocol or an approved amendment, and for its secure storage and safe handling throughout the study.

3.5 Reporting of safety issues breaches of the protocol or ICH GCP

In the event of any prohibition or restriction imposed by an applicable CA, or if the Investigator is aware of any information which might influence the evaluation of the benefits and risks of the IMP, Inventiva should be informed immediately!

In addition, the Investigator will inform Inventiva immediately of any urgence safety measures taken by the Investigator to protect the study patients against any immediate hazard, and of any serious breaches of this protocol or of ICH GCP that the Investigator becomes aware of.

According to Regulation 29A of the Medicines for Human Use Regulations 2004, as amended by Statutory Instrument 2006/1928 - Guidance for the Notification of Serious Breaches of GCP or the Trial Protocol:

A serious breach is a breach which is likely to effect to a significant degree:

- The safety or physical integrity of the subjects of the trial or

- The scientific value of the trial.

The Sponsor or a person legally authorised by the Sponsor shall notify the licencing authority in writing of any serious breach:

- The conditions and principles of GCP in connection with that trial or

- The protocol relating to that trial within 7 days of becoming aware of that breach.

3.6 Insurance

In case of a damage or injury occurring to a patient in association with the IMP or the participation in the study, the Sponsor has subscribed to an insurance policy covering, in its terms and provisions, its legal liability for injuries caused to participating persons and arising out of this research performed strictly in accordance with the scientific protocol as well as with applicable law and professional standards.

The Sponsor is insured under a liability insurance program subscribed by the Sponsor to cover its liability as a Sponsor of clinical studies on a worldwide basis. All relevant insurance documentation is included in the file submitted to authorities approval of which is required. Each participating Investigator receives the statement on insurance policy applicable in his/her country for filing in the Investigator's Study File.

A copy of the certificate is filed in each Investigator site file and the Trial Master File.

3.7 Patient Information and Consent

The Investigator (according to applicable regulatory requirements), or a person delegated by the Investigator, should fully inform the patient of all pertinent aspects of the clinical trial including the written information given approval/favorable opinion by the Ethics Committee (IRB/IEC).

Prior to a patient's participation in the clinical trial, the Informed Consent Form should be signed and personally dated by the patient or by the patient's legally acceptable representative, and by the person who conducted the informed consent discussion.

The Informed Consent Form used by the Investigator for obtaining the patient's informed consent must be reviewed and approved by the Sponsor prior to submission to the appropriate Ethics Committee (IRB/IEC) for approval/favorable opinion.

Signed consent forms must remain in the Investigator file and must be available for verification by study monitors or authorized regulatory representatives at any time.

The patient should receive a copy of the signed and dated written Informed Consent Form. Any amendments to the written information will be provided to the patients.

3.8 Premature Termination

Both the Investigator and the Sponsor reserve the right to terminate the study at any time. Should this become necessary, the procedures will be agreed after consultation between the two parties. In terminating the study, the Sponsor and the Principal Investigator will ensure that adequate consideration is given to the protection of the best interests of the patients.

3.9 Definition of end of the trial

End of trial in a Member State of the European Union is defined as the last visit (Visit 4, planned at the end of the 30-day follow-up period), of the last patient as defined in the protocol in all the concerned-Member State. Poor recruitment (recruiting less that the anticipated number in the

CTA) by a Member State is not a reason for premature termination but considered as normal conclusion to the study in that Member State.

4. Study Objectives and Investigational Plan

The study objective is to assess the safety and the efficacy on the activity part of the SAF histological score (inflammation and ballooning) of a 24-week treatment with two doses of lanifibranor (800, 1200 mg/24h) in NASH adult patients.

4.1 Study Design

The study is a three arm (placebo, lanifibranor 800 and 1200 mg/day), randomized (1:1:1), double-blind, placebo-controlled, multicenter, 24-week treatment study.

The goal of the proposed study is to show improvement of cardinal NASH histopathologic features as well as safety data after a 6-month treatment.

The study will require to randomize 225 NASH patients (*i.e.* at least 300 screened patients, see section 9.2 for sample size description). The overall study design is shown in Figure 1.

Patients will undergo 6 visits: screening visit (V-1, -8 weeks to -2 weeks), randomization (V0), interim visit 1 (V1, 4 weeks after V0), interim visit 2 (V2, 10 weeks after V1), End of treatment visit (V3, 10 weeks after V2) and a follow-up visit (4 weeks after V3). A detailed list of procedures performed at each visit is presented in section 5.

As the main efficacy goal of the study is to assess treatment effect on histologic features of NASH, there will be a centralized reading of slides of a liver biopsy to confirm the diagnosis and assess the severity at screeing phase (V-1) and end of treatment visit (V3). At the screening phase, the liver biopsy could either have been performed within 6 months before the screening period or performed during the screening process after confirmation of the patient eligibility regarding all others inclusion/exclusion criteria (incl. biological values, physical examination, ECG, FibroScan, and LMS in selected centers). The liver biopsy at the end of the treatment period will be performed as part of the visit 3 (week 24).

Clinical and laboratory safety assessments will be performed at each visit. ECG will be performed at screening, interim visit 1 and end of treatment and will be read centrally.

A FibroScan™ will be performed at screening and end of treatment visit to assess Transient Elastography (TE) and Controlled Attenuation Parameter (CAP).

Inflammatory, glucose metabolism and lipid metabolism markers will also be assessed as well as exploratory biomarkers of NASH activity or PPARs agonist activities.

4.1.1 *Screening Phase*

For the patients with a biopsy performed within 6 months before screening, all diagnostic tests regarding NASH (including a liver biopsy), HCV and the elimination of other causes of ALT elevation must have been performed before the patient would be considered for inclusion in the study as part of the routine medical care and will not be performed specifically for the study.

The goal of the screening is therefore to check inclusion and exclusion criteria mainly by centralized reading of a previous liver biopsy and centralized laboratory tests. For Women with childbearing potential, a serum pregnancy test will also be performed (βHCG testing) at the screening visit.

The screening phase will be of at least of 10 working days, but of no more than 8 weeks, to allow performing the centralized review of liver biopsy and ECG.

For the patients without liver biopsy performed within 6 months before screening, the liver biopsy will be part of the screening tests. However, to avoid unnecessary biopsies, the screening process should be performed according to the 3 sequential steps described below:

Step 1: Review of the medical history, concomitant medications, physical examination, electrocardiogram, biological sampling and FibroScan (mandatory). Patients will be withdrawn from the study at this step in case of TE > 12.5 kPa (i.e. high probability of cirrhosis).

Step 2: MRI/Liver MultiScan technology, only in selected sites. Patients will be withdrawn from the study at this step in case of LIF < 2 or MRI-PDFF ≤5% (see section 5.2.8).

Step 3: A liver biopsy will be performed. In all cases, liver biopsy will be performed upon medical indication and according to the standard practice of the site.

After validation of the histological criteria by the central reading assessment, patients can be randomized.

4.1.2 *Treatment Period*

At the randomization visit, 75 patients in each group will receive either placebo or lanifibranor 800 mg/day or lanifibranor 1200 mg/day for 24 weeks.

4.1.3 *Follow-up Period*

Safety of withdrawal from treatment groups is assessed during a 30-day assessment phase.

4.1.4 *Patient management at the end of treatment*

The trial treatment is stopped after V3.

After the follow-up period (V4, 4 weeks after V3) the Investigator/hepatologists/general practitioner will decide according to each patient, the need to use the lifestyle changes recommendations whenever appropriate and treatment of comorbidities which may play a role in NASH such as, for example, diabetes and hyperlipidaemia.

If the study is positive there will be further trials for which these patients may be eligible.

4.1.5 *Total study duration*

The total duration of the study is up to 38 months, i.e. at least 30 months of enrollment period and up to 8 months of patient participation.

4.2 Study Population

4.2.1 *Inclusion Criteria*

1. Adult subjects, age ≥18 years.
2. NASH histological diagnosis according to the currently accepted definition of both EASL and AASLD (62,66,67), requiring the combined presence of steatosis (any degree ≥ 5%) + lobular inflammation of any degree + liver cell ballooning of any amount, on a liver biopsy performed ≤ 6 months before screening in the study and confirmed by centralized reading during the screening period *and*
    a. SAF Activity score of 3 or 4 (>2)

b. SAF Steatosis score ≥ 1

c. SAF Fibrosis score: < 4.

3. Subject agrees to have a liver biopsy performed after 24 weeks of treatment.

4. Compensated liver disease with the following hematologic and biochemical criteria before randomization:
   - ALT < 10xULN
   - Hemoglobin ≥ 110 g/L (11 g/dL) for females and ≥ 120 g/L (12 g/dL) for males
   - White blood cell (WBC) > 2.5 10^9/L (2.5 10^3/µL)
   - Neutrophil count > 1.5 10^9/L (1.5 10^3/µL)
   - Platelets > 100 10^9/L (100 10^3/µL)
   - Total bilirubin < 35 µmol/L (2.06 mg/dL). Patients with bilirubin ≥ 35 µmol/L can be included if non-conjugated bilirubin in the setting of a Gilbert's syndrome.
   - Albumin > 36 g/L (3.6 g/dL)
   - INR < 1.4
   - Serum creatinin < 115 µmol/L (1.3 mg/dL) (men) or < 97 µmol/L (1.1 mg/dL) (women) or estimated glomerular filtration rate ≥ 60 mL/min/1.73m^2

5. No other causes of chronic liver disease (autoimmune, primary biliary cholangitis, HBV,HCV, Wilson's, α-1-antitrypsin deficiency, hemochromatosis, etc…) considered to have an impact on the patient's safety or on the efficacy evaluation.

6. If applicable, have a stable type 2 diabetes, defined as HbA1c ≤ 8.5% and fasting glycemia <10 mmol/L (180 mg/dL), no introduction of new medication in the previous 6 months, and no new symptoms associated with decompensated diabetes in the previous 3 months. Minor modifications of anti-diabetic treatments or dosages are allowed if done in a context of stable type 2 diabetes, i.e. HbA1c ≤ 8.5% in the previous 6 months.

7. Have a stable weight since the liver biopsy was performed defined by no more than a 5% loss of initial body weight.

8. Negative pregnancy test or post-menopausal. Women with childbearing potential (i.e. fertile, following menarche and until becoming post-menopausal unless permanently sterile) must be using a highly effective method of contraception (i.e. combined (estrogen and progestogen containing) hormonal/ progestogen-only hormonal contraception associated with inhibition of ovulation, intrauterine device, intrauterine hormone-releasing system, bilateral tubal occlusion, vasectomised partner). The contraceptive method will have to be followed for at least one menstruation cycle after the end of the study

9. Subjects having given her/his written informed consent.

4.2.2 *Exclusion Criteria*

1. Evidence of another form of liver disease considered to have an impact on the patient's safety or on the efficacy evaluation.

2. History of sustained excess alcohol ingestion in the year before the pre-study treatment biopsy: daily alcohol consumption > 30 g/day (3 drinks per day) for males and > 20 g/day (2 drinks/day) for females.

3. Unstable metabolic condition: Weight change > 5% in the last three months, diabetes with poor glycemic control (HbA1c > 8.5%), introduction of an antidiabetic or of an anti-obesity drug or restrictive bariatric (weight loss) surgery in the past 6 months prior to screening.
4. History of gastrointestinal malabsorptive bariatric surgery within less than 5 years or ingestion of drugs known to produce hepatic steatosis including oral corticosteroids (at dose >5mg/day prednisone equivalent), estrogens (at doses greater than those used for hormone replacement or contraception), tamoxifen, methotrexate, tetracycline or amiodarone in the previous 6 months. Corticosteroids administered by routes other than oral are allowed. One short (<2 weeks) course of oral corticosteroids, more than 3 months before the pre-study treatment biopsy is also allowed.
5. Significant systemic or major illnesses other than liver disease, including congestive heart failure (class C and D of the AHA), unstable coronary artery disease, cerebrovascular disease, pulmonary disease, renal failure, organ transplantation, serious psychiatric disease, malignancy that, in the opinion of the Investigator, would preclude treatment with lanifibranor and/or adequate follow up.
6. HBs antigen >0, HCV PCR >0 (patients with a history of HCV infection can be included if HCV PCR is negative since more than 3 years), HIV infection.
7. Pregnancy or lactation or inability to adhere to adequate contraception in women of child-bearing potential.
8. Active malignancy except cutaneous basocellular carcinoma.
9. Any other condition which, in the opinion of the Investigator would impede competence or compliance or possibly hinder completion of the study.
10. Body mass index (BMI) >45 kg/m^2.
11. Type 1 diabetes and type 2 diabetic patient on insulin.
12. Diabetic ketoacidosis
13. Fasting Triglycerides > 300 mg/dL (3.39 mmol/L).
14. Hemostasis disorders or current treatment with anticoagulants.
15. Contra-indication to liver biopsy.
16. History of, or current cardiac dysrhythmias and/or a history of cardiovascular disease event, including myocardial infarction, except patients with only well controlled hypertension. Any clinically significant ECG abnormality reported by central ECG reading confirmed by the Investigator to be Clinically Significant.
17. Participation in any other investigational drug study within the previous 3 months.
18. Have a known hypersensitivity to any of the ingredients or excipients of the IMP including: Lactose monohydrate, Hypromellose, Sodium laurilsulfate, Sodium starch glycolate (type A), Magnesium stearate, Opadry™ II 85F18422
19. Be possibly dependent on the Investigator or the Sponsor (e.g., including, but not limited to, affiliated employee).
20. Creatine phosphokinase (CPK) >5 x ULN

21. Patient with history of well documented osteopenia. Patient treated with vitamin D and/or Calcium based supplements for preventive reasons can be included.

(The criteria below are applicable only for patients who will undergo a MRI/LMS in selected centers)

22. Claustrophobia to a degree that prevents tolerance of MRI scanning procedure. Sedation is permitted at discretion of Investigator.
23. Metallic implant of any sort that prevents MRI examination including, but not limited to: aneurysm clips, metallic foreign body, vascular grafts or cardiac implants, neural stimulator, metallic contraceptive device, tattoo, body piercing that cannot be removed, cochlear implant; or any other contraindication to MRI examination.

4.2.3 *Prohibited concomitant medications*

If not specified, the following treatment must be stopped at the latest before randomization

- PPAR Gamma agonists, PPAR Alpha agonists (fibrates), ezetimibe
- Bile salts chelators, phytosterols, fish oils,
- Glucagon like peptide-1 receptor agonists (incl. litaglutide, exenatide),
- Insulin,
- Vitamins E (alpha-tocopherol),
- Anticoagulants (incl. warfarin, dabigatran, rivaroxaban, apixaban),
- Oral corticosteroids.

4.2.4 *Allowable medications for standard care or precautions*

- ***Obesity:*** stable weight since the liver biopsy was performed, defined by no more than a 5 % loss of initial body weight (cfr inclusion criteria 7).
- ***Treatment used for the underlying medical condition***

  Treatments are allowed within certain restrictions (described above and below) and provided they have been kept at ***stable doses for at least 6 months before the pre-treatment biopsy***.

  Type 2 diabetes:

  - Metformin,
  - Dipeptidyl peptidase-4 inhibitors,
  - Sodium-glucose transport protein 2 inhibitors: canagliflozin, dapagliflozin and empagliflozin.

  Minor modification of anti-diabetic treatments or dosages are allowed if done in the context of stable type 2 diabetes, i.e. HbA1c ≤8.5% in the previous 6 months.

  Hyperlipidemia: only statins at stable doses within the past 3 months before pre-study treatment biopsy will be allowed.

  Antiplatelets agents: The antiplatelets agents (incl. low-dose aspirin, ticlopidine, clopidogrel, prasugrel, ticagrelor) are allowed.

Herbal supplements and Others Supplementation: Herbal preparations or vitamin supplements with no exact composition known should not be started during the course of the study as they could be liver toxic.

- ***Other Medications***

  Medications other than the IMP and those mentioned above should only be started during the course of the study with the agreement of the Investigator in order to avoid interference with study assessments. The need for other medication may lead to exclusion of the patient from the study.

  If symptomatic medication is needed to treat adverse events related to IMP, the Investigator will inform the Sponsor about the concomitant medication given.

4.2.5 *Enrolment*

If a patient is not eligible, the main reason for non-inclusion will be documented in the source document and the screening log. After eligibility is confirmed, patients will be assigned a treatment number. Treatment numbers will be allocated in ascending order, the order in which patients are included. The Investigator or a staff member will enter the patient initials and number in a web-based electronic Case Report Form (eCRF), the confidential patient identification list and the drug dispensing log.

4.3 Study Medication

4.3.1 *Identity of Investigational Medicinal Product(s) (IMP)*

The lanifibranor tablets and Placebo To Match (PTM) are white to off-white, ovale shape, bi-convex, film-coated tablets:

- Each active tablet contains 400 mg of the active substance lanifibranor in an immediate release formulation. The chemical name of the active substance is 1-(6-benzothiazolylsulfonyl)-5-chloro-1H-indole-2-butanoic acid.
- The placebo tablet contains a physical mixture of lactose monohydrate, microcrystalline cellulose, and sodium starch glycolate and magnesium stearate.

4.3.2 *Treatments Administered*

This double blinded placebo controlled study involves a therapeutic dose of 800 mg lanifibranor (qd) and 1200 mg lanifibranor (qd).

The lanifibranor tablets and PTM are packaged into High-density polyethylene (HDPE) bottles of 100 ml (38 tablets for a treatment period of one month, plus a use margin of 1 week) with proper closures fitted with a silicagel cartridge. Three bottles each (either active or placebo) and labelled A, B and C with a colored circle sticker are assembled in a carton to compose monthly Kits as detailed below:

- Placebo Kit: Morning (P+P+P)
- 800 mg Kit: Morning (P+A+A)
- 1200 mg Kit: Morning (A+A+A)

Where P denotes the placebo tablet and A denotes the active tablet (400 mg).

Figure 4: IMP Packaging

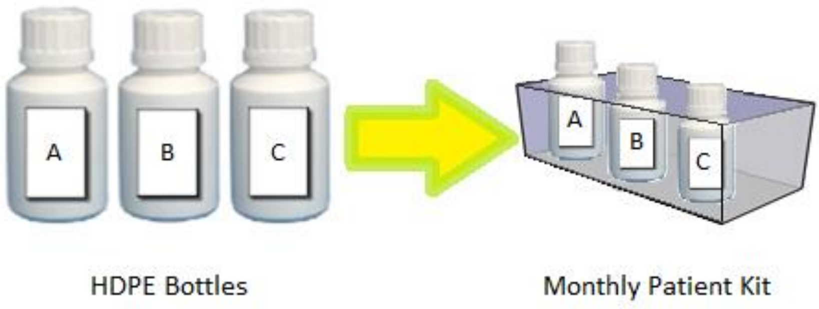

A total of 5 Patient Kits is representing the treatment per patient for the clinical study.

4.3.3 *Medication Dispensing*

The patients receive at each visit one kit per month (i.e one kit at V0, and 2 kits at V1 and V2). They are instructed to take morning 3 tablets with food, i.e. one tablet from each of the containers A, B, and C. The overall treatment duration is 24 weeks.

Dosage modifications are not permitted. Should the patient or the Investigator for any reason wish to discontinue treatment, they may do so at any time. The date of the last dose and or any dosage modifications by the patient are documented in the eCRF.

4.3.4 *Selection and timing of Dose for Each Patient*

The treatment arm will be determined by a centralized electronic randomization at the inclusion visit.

4.3.5 *Labeling*

Packaging and labelling is performed by [redacted]

The immediate containers (bottles) and the outer packaging (medication boxes) will contain the information required by national regulations as described in the requirements of Directive 2003/94/EC.

Table 5: Summary of Labelling Details

Pharmaceutical dosage form	38 tablets of Lanifibranor 400 mg or Placebo to Match. For Oral Use
Batch N°:	XXXX
Bottle ID:	XXXX
Kit N°:	XXXX
Protocol ID:	IVA_01_337_HNAS_16_002
Sponsor:	Inventiva SA, 50, rue de Dijon, 21121 Daix, France<br>Telephone: +33 3 80 44 75 00
Direction for Use:	Take 3 tablets by mouth once daily with food (morning), one from bottle A, one from bottle B, and one from bottle C.
For Clinical Trial Use Only	Return all empty bottles or unused medication to the trial Centre at the next visit.
Storage:	Store in original container at 25°C or below. Do not freeze.
Expiry Date:	MM/YYYY
Keep Out of Reach of Children.<br>**Caution*: New Drug – Limited by Federal (or United States) law to investigational use.**	

* for US only

4.3.6 *Accountability*

The Investigator will maintain accountability records showing the quantities of IMP received at the study site and dispensed to each patient. Any unused IMP, including empty or partially used containers, will be accounted for and then destroyed. At the time of return to the Sponsor, the Investigator must verify that all unused or partially used investigational medicinal product supplies have been returned by the clinical study subject and that no remaining supplies are in the Investigator's possession.

4.3.7 *Treatment Compliance*

The following means will be taken to improve compliance to treatment:

- the Investigator will attempt to select patients able to understand and to comply with instructions,
- the patient should be instructed to return containers at the end of the treatment period,
- remaining tablets will be counted /recorded in the eCRF by sites and monitored by the Clinical Research Associate

4.3.8 *Return or destruction of investigational products*

The products are collected by the Sponsor at the end of the study and are thereafter destroyed.

5. Measurements Assessed and Study Flow Chart

The following section presents the assessments performed at each visit. Target dates are ± 3 days.

5.1 Table of study procedures and assessments

A table is provided in the schedule of Study Procedures (see Table 1 and Table 2).

5.2 Specific procedures for Assessment of outcomes

5.2.1 *Liver Biopsy*

For each patient, five slides of a liver biopsy have to be sent for a central reading assessment, at screening for the validation of the histological inclusion criteria, and at visit 3 for the evaluation of the efficacy evaluation.

At screening visit (V-1), if no slides are available from a liver biopsy performed within the past six months, a liver biopsy will be performed as part of the screening process after having checked that all non-histological inclusion/exclusion criteria are validated (i.e. biological values, ECG, Fibroscan and LMS if applicable).

At end-of-treatment visit (V3), a liver biopsy is performed.

- *Procedures recommendation*

The liver biopsy will be performed according to each site local procedures and the recommendation below.

A large needle (>19G), preferentially 16G, should be used in order to obtain biopsy samples that meet the quality requirements for accurate diagnosis.

Where applicable, antiplatelets agents (i.e. aspirin, ticlopidine, clopidogrel, prasugrel, ticagrelor) will be stopped 10 days before the liver biopsy and will be resumed after 48-72 hours as per guidelines (68). After the procedure, each patient will be monitored during a minimal observation period of 4 hours or more depending on local practices. After discharge from hospital and for a minimum of 48 hours after the liver biopsy, patients will be instructed not to lift heavy weights (68).

- *Slides preparation and shipment*

Fixation and paraffin embedding of the biopsies will be performed according to local procedure and according to precautions detailed in Appendix C.

Five slides have to be sent to [redacted] for a quality control and staining (if requested, see Appendix C for details). The handling of the sample must be prepared and provided with the supplied kit by the Sponsor. Afterwards, the slides will be shipped without delay to [redacted] for the central reading.

All biopsy slides will be stored at [redacted] until the end of the study and then send back to sites. If deemed necessary at the time of the availability of the study results, complementary immunohistochemistry analyses could be performed within the year after the end of the study. In that case, if appropriate, the central laboratory will be responsible to send the slides to the laboratory(ies) in charge of these analyses. Until this timepoint, upon Investigator's request, the biopsy slides might be sent back to the site on an ongoing basis.

5.2.2 *Urine samples*

Urine Samples will be collected at V0 *(including urine pregnancy test),* V2, V3 and V4. Urine analysis by dipstick evaluating hematuria will be done on site. Urine samples will then be stored for further assessment and/or for safety evaluation. Urine specimens will be obtained by providing participants with a collection cup and instructions for collecting as described in the sampling kit supplied by the Sponsor via the central laboratory.

Safety assessments may be performed on the urine samples in case of renal function impairment is observed during the study. Albuminuria and other biomarker like cystatin C will be tested, and

aliquots of urine samples will be stored in case where a urine biomarker specific of NASH is validated during the study.

The tube labels need to contain the following information: Study Site, Subject Number, Day, Time, Visit number

All urines samples will be destroyed by the central laboratory at the end of a scheduled storage period (1 year after the end of the study).

5.2.3 *Blood samples taken*

Blood sample taken are performed at every visit, i.e. V-1 (screening) to V4 (Follow-up). The patient must be fasting overnight from 10:00 pm. Alternatively, blood sampling can be performed in the afternoon provided patients have been fasting for at least eight hours. Total blood volume sampled during the study for each patient (for pharmacokinetics, biomarkers, safety and efficacy biological tests) will be as follows:

Table 6 : Blood volume taken at each visit per type

**Total Blood volume per Sample Type (mL)**	**Screen**	**Re-test***	**V0**	**V1**	**V2**	**V3**	**V4**	***Total***
Serum (SST)	8.5	8.5	34	8.5	8.5	34	8.5	*102*
Whole Blood (EDTA)	3	3	6	3	3	3	3	*21*
Plasma (EDTA)	-	-	3	-	-	3	-	*6*
Plasma (Citrated)	2.7	2.7	2.7	2.7	2.7	2.7	2.7	*16.2*
Plasma (Fluoride-oxalate)	2	2	2	2	2	2	2	*12*
Plasma (Lithium Heparin)	-	-		3	-	3	-	*6*
**Total Blood Volume per visit (mL)**	**16.2**	**16.2**	**47.7**	**19.2**	**16.2**	**47.7**	**16.2**	***179.4***

*if screening period > 4 weeks

Attention must be paid to the kind of blood sample required (Biobank, Safety, Pharmacokinetics) as they vary between visits.

For the monitoring of the individual subject, each sample should be collected under the same conditions (e.g. time ± 1h) as the baseline sample. This is to overcome the effects of the circadian variation of the biomarkers measured.

The samples need be collected at the clinical sites within 48 hours and shipped on dry ice. The tube labels need to contain the following information: Study Site, Subject Number, Day, Time, Visit number, Aliquot number, Sampling times and dates also need to be recorded on the eCRF.

The central laboratory needs be notified of a shipment at least 3 days prior to shipment arrival. The notification should include the study number, the number of samples, the Airway Bill Number, the estimated time of arrival (ETA) and an electronic sample list.

More detailed instructions for handling and preparing the samples are provided with the kit supplied by the Sponsor via the central laboratory.

All blood samples will be destroyed by the central laboratory at the end of a scheduled storage period (1 year after the end of the study).

5.2.4 *Waist measurement*

The Waist measurement must be done at midway between the top of the hip bone and the bottom of the ribs during a normal breath-out (Appendix B).

5.2.5 *Electrocardiogram*

12-lead ECGs will be performed at Screening (V-1), interim visit 1 (V1) and the end-of-treatment visit (V3) and a central assessment will be performed by a specialized CRO.

Two originals ECGs print out using an A4 format (no photocopies) have to be recorded in order to send a blinded one to the CRO (attached with a transmission Form), the second one to be keep in patient's medical file.

Please refer to Appendix B for the detailed technical procedure.

5.2.6 *Vital signs*

Blood pressure will be measured by using a sphygmomanometer. The results will be recorded in millimeters of mercury (mmHg). Pulse rate will be measured for 30 seconds and will be recorded as beats/minute. Blood pressure and pulse will be measured lying after at least a 10-minute rest.

5.2.7 *Fibroscan®*

A Fibroscan® will be performed at screening visit and end of treatment visit (V3), if available.

In case of no historical slides from a liver biopsy performed within 6 months prior the screening process, Friboscan® will be mandatory before scheduling a liver biopsy.

TE and CAP will be evaluated by using Fibroscan® technology. Fibroscan® is a totally non-invasive and completely painless ultrasound technique that will not require the use of anaesthesia or sedation. Any size of probe (M, XL) can be used, but it will have to be reported in the eCRF.

TE is an approved parameter for the diagnosis of cirrhosis in patients with chronic liver diseases. In this study, an optimal cut-off of 12.5 kPa (patients with TE>12.5 kPa) will be set for the discrimination of cirrhotic patients (with negative predictive value higher than 90%) as defined in the Non-invasive tests for liver disease diagnosis guidelines (69).

If available at site, CAP (expressed in $dB.m^{-1}$) will be recorded in the eCRF. CAP has the ability to quantify and detect hepatic steatosis from 10% of fatty infiltration and is not influenced by liver fibrosis and cirrhosis (70–73).

5.2.8 *Liver Multiscan*

Where available, the subject's liver will be evaluated using Liver*MultiScan* (non-invasive MRI technology). Liver*MultiScan* (LMS) is non-invasive and completely painless magnetic resonance technique that will not require the use of anaesthesia, sedation or contrast injection. The imaging protocol takes just 10 minutes, in which parametric maps are taken of the liver, using quantitative imaging. This will allow the study doctor to measure the levels of fat, iron and fibroinflammatory disease.

The quantitative images taken using MR based LiverMultiScan technique will be used to measure 1) hepatic iron content, measured by T2* (ms) and presented as mg/g of dry weight liver, 2) hepatic fat content, measured by IDEAL (MRI-PDFF) and presented as %, and 3) cT1 or iron-corrected T1 that correlates with liver fibrosis and inflammation, derived by an algorithmic correction of T1 (ms) by T2* and presented in ms. cT1 is additionally reported as Perspectum Diagnostics' proprietary metric, the Liver Inflammation and Fibrosis (LIF) score. An MRI-PDFF cut-off of >5% indicates hepatic steatosis, and in this study, a cutoff of LIF <2 will be set to rule

out subjects without significant fibroinflammatory disease (eg NASH). Consider biopsy for subjects with LIF≥2 and MRI-PDFF >5%.

This cT1 metric is standardized across all MRI manufacturers, and presented as the LIF score for easy patient and physician communication. Conditions which increase the extracellular fluid content, such as inflammation and fibrosis, cause a higher LIF score and the LIF score has been shown to predict clinical outcomes in patients with chronic liver disease, as well as correlating with biopsy.

Multiparametric MR is a tool for the evaluation of patients with NAFLD/NASH that provides reliable data more frequently compared to transient elastography, with no differences in the diagnostic accuracy for significant fibrosis or cirrhosis. Furthermore, multiparametric MR has good accuracy for the diagnosis of NASH and ballooning. The particular strength of the MR technique is the ability to assess both the necro-inflammatory and fibrotic components of NASH in a single test that allows accurate evaluation of the overall disease severity (74).

5.2.9 *Quality of Life questionnaire*

SF-36: The SF-36 is a multi-purpose, short-form health survey with 36 questions. It yields an 8-scale profile of functional health and well-being scores as well as psychometrically-based physical and mental health summary measures and a preference-based health utility index (http://www.sf-36.org/tools/sf36.shtml, Appendix D).

FFS: The Flinders Fatigue scale (FFS) is a self-report questionnaire assessing daytime fatigue related to poor sleep. It is a relatively brief measure, containing only 7 items that provide a global measure of fatigue (http://dspace.flinders.edu.au/xmlui/handle/2328/10181, Appendix E)

The patient's information sheet and the patient questionnaires are provided in the local language. The Investigator and medical staff are available for explanations and questions, at the visit and via telephone thereafter.

5.3 Procedures at each visit

5.3.1 *Screening (V-1)*

As part of the patient's direct clinical care team, only the referent doctor will be able to invite potential patients to participate to the study according to his/her patient medical records. The referent doctor will go through the information sheet and answer any questions that the patient may have. The patient will also have the ability to discuss his/her participation with family, friends or family doctor (general practioner: GP).

The patient will be asked to sign and date the attached "Patient Informed Consent" form, prior to any study procedure being performed.

The pre-treatment procedures are listed in the Schedule of Study Procedures (Table 1). Details are provided in section 5.2 of the protocol and in the eCRF.

Blood sampling: Kits with supplies and instructions are provided for the safety blood sampling and a serology test to exclude HIV infection will also be performed.

Fibroscan®: This exam is mandatory if a liver biopsy is done at screening, in order to avoid this procedure in patients with high risk of cirrhosis.

Liver Multiscan: This exam is mandatory in selected centers to assess patient's eligibility to the liver biopsy (if not performed within the past 6 months).

Electrocardiogram: An ECG has to be performed and centrally assessed to validate inclusion and exclusion criteria.

Liver Biopsy: for the patients who do not have a liver biopsy performed within 6 months before screening.

Liver Biopsy Centralized Reading: Details on how to perform the reading of the liver biopsy are provided in Section 5.2.1.

Safety: Any untoward medical event, which occurs from the time of signed Informed Consent to the time of first IMP administration, will be classified as "non treatment-emergent AE

5.3.2 *Retest visit (additional visit during screening process)*

A retest visit could be performed if requested by Investigators and/or Sponsor to confirm/invalidate abnormal biological values at screening process. This retest will be mandatory if the total duration of the screening period exceed 4 weeks (i.e. inclusion/non-inclusion criteria related to safety laboratory tests have to be checked with results of less than 4 weeks before randomization).

The randomization could be done only after receipt of the retest reports and validation of the results by the Investigators and/or Sponsor Referent Physician.

5.3.3 *Randomization and Beginning of the Treatment Period (V0)*

At this visit, the eligibility of the patient is confirmed, and the treatment starts.

The assessments to be performed are listed in the Schedule of Study Procedures (Table 1).

Blood sampling: Kits with supplies and instructions are provided for the safety, efficacy, biobank, pharmacokinetics and genotype blood sampling as early as possible in the morning. Blood samples will be taken in the morning after overnight fasting. The patient must be fasting overnight from 10:00 pm. Alternatively, blood sampling can be performed in the afternoon provided patients have been fasting for at least eight hours.

Safety: AEs that have occurred since the last visit are recorded, irrespective of a potential relationship with the concomitant background treatment. Please observe the reporting requirements for AEs (see Section 6).

Randomization: The randomization code is provided through an online system after identification with a login and a password. Once some details are entered, the system lets you know immediately, which medication kit to use for this particular patient.

Treatment: The patient receives one monthly kit (packaging described in Figure 4), and they are carefully instructed how to take the drug and the importance of bringing back to the site the unused medication at each visit. Patients need to take morning 3 tablets with food, i.e. one tablet from each of the containers A, B, and C.

The date of the next visit in 4 weeks is agreed. The time window for the visits is +/- 3 days.

5.3.4 *Intermediate Study Visit 1 to Visit 2 (V1, Week 4 and V2, Week 14)*

Important: Please note that the visits are not at regular intervals and the assessments vary.

The assessments to be performed are listed in the Schedule of Study Procedures (Table 1).

Treatment: It is therefore important to ensure each time, that the patient has sufficient medication until the next visit. It may be necessary to distribute 2 monthly kits to cover the time to the next visit. The last distribution takes place on Visit 2. Patients are carefully instructed

how to take the drug and the importance of bringing back to the site the unused medication, so that drug accountability can be verified and completed.

Blood sampling: Kits with supplies and instructions are provided for the safety and efficacy blood sampling.

Electrocardiogram (only at V1): An ECG has to be performed and centrally assessed to evaluate the safety of the patients.

The patients withdrawn early from the study should, if at all possible, undergo the Visit 3 assessments and procedures.

Please observe the reporting requirements for AEs (see Section 6).

5.3.5 *End of Treatment Visit or withdrawal (V3, Week 24)*

Visit 3 is the therapy-end visit.The assessments to be performed are listed in the Schedule of Study procedures (Table 1).

Blood sampling: Kits with supplies and instructions are provided for the safety, efficacy, Biobank and pharmacokinetics blood sampling.

Fibroscan®: The parameters recorded will be correlated to those obtain with the liver biopsy performed at the end of treatment.

Electrocardiogram: An ECG has to be performed and centrally assessed to evaluate the safety of the patients.

Liver biopsy: Details on how to perform the biopsy are provided in Section 5.2.1.

Please observe the reporting requirements for AEs (see Section 6).

Important: In case of early withdrawal, the following (i.e. follow-up post-treatment visit, 4 weeks after the end of treatment) should also be performed whenever possible.

5.3.6 *Follow-up Post-Treatment Visit, End of Study Visit (V4, Week 28)*

A follow-up visit takes place 4 weeks after the end of treatment. The assessments to be performed are listed in the Schedule of Study Procedures (Table 1).

Blood sampling: Kits with supplies and instructions are provided for the safety and efficacy blood sampling.

Please observe the reporting requirements for AEs (see Section 6).

5.3.7 *Unscheduled/safety Visits*

During the all treatment and the follow-up period, the Investigator may conduct unscheduled visit that may perform again study procedures, including vital signs assessments, blood sample taken and physical examination. These visits will have to be motivated by the Investigator in the aim of guarantee the safety of the patients.

Specific events listed bellow, will automatically generate an Unscheduled/safety visit:

- ALT increase of 2 times of the baseline value at V0: A safety visit has to be performed 15 days later. A blood taken will be performed to check the ALT level.
- ALT increase of 5 times the upper limit of the normal range (ULN): A safety visit has to be performed 15 days later. A blood taken will be performed to check the ALT level

Please refer to paragraph 10 for withdrawal criteria.

6. Safety Assessment

6.1 Adverse Events

6.1.1 *Definitions accroding to ICH guidelines*

- **Adverse event:** Any untoward medical occurrence in a patient or clinical investigation subject administered a medicinal product and which does not necessarily have a causal relationship with this treatment. An AE can therefore be any unfavourable and unintended sign (including an abnormal laboratory finding), symptom, or disease temporally associated with the use of an IMP, whether or not considered related to the IMP.
- **Adverse reaction of an IMP**: All untoward and unintended responses to an investigational medicinal product related to any dose administered.

All adverse events judged by either the reporting Investigator or the Sponsor as having a reasonable causal relationship (i.e. possible/probable) to a medicinal product qualify as adverse reactions. The expression reasonable causal relationship means to convey in general that there is evidence or arguments to suggest a causal relationship.

6.1.2 *Collection*

The condition of the patients will be monitored throughout the study. Subjective symptoms reported spontaneously by the patient as well as signs observed by medical staff will be recorded in the source records and the eCRF.

Any AE (including laboratory test abnormalities, intercurrent illnesses or injuries, and/or study procedures related AE) reported spontaneously by the subjects, or observed by the Investigator, will be recorded in the trial database.

Any untoward medical event, which occurs from the time of signed Informed Consent to the time of first IMP administration, will be classified as “non treatment-emergent AE”.

6.1.3 *Intensity Rating*

The intensity of an AE will be rated as follows:

- Mild: no interference with the subject’s daily activities and does not require mandatory corrective/symptomatic treatment.
- Moderate: moderate interference with the subject’s daily activities and/or requires minimal medical intervention or corrective treatment required.
- Severe: major and unacceptable interference with the subject’s daily activities and requires mandatory corrective/symptomatic treatment, possible hospitalization.

6.1.4 *Causality Rating*

The causal relationship of an AE to the IMP will be rated as follows:

- Unrelated: Clearly and incontrovertibly due only to extraneous causes, and does not meet criteria listed under unlikely, possible or probable.
- Unlikely: Does not follow a reasonable temporal sequence from administration of the IMP, or is most likely related to another etiology than the trial drug such as the patient’s clinical state, environmental factors or other therapies.
- Possible: Follows a reasonable temporal sequence from administration of the IMP, and/or a causal relationship cannot be excluded and remains likely.

- Probable: good reason (such as clear-cut temporal association with improvement on cessation of the IMP or reduction in dose, or reappears upon (accidental) rechallenge, or follows a known pattern of response to the IMP) and sufficient documentation to assume a causal relationship.

6.1.5 *Action Taken*

The action taken with the IMP for an AE will be rated as product withdrawn, temporary interruption, or dose not changed. AEs requiring therapy will be treated with recognized standards of medical care to protect the health and the well-being of the patient.

6.1.6 *Outcome*

The outcome of an AE will be rated as recovered, recovering, sequelae, not recovered, fatal or unknown. The Investigator will follow up any AE until it is resolved or until the medical condition of the patient is stable. All relevant follow-up information will be collected.

For AEs that are ongoing at the last visit, the Investigator will make thorough efforts to document the outcome in the eCRF, until 28 days after end of study treatment for AEs considered by the Investigator as unrelated or unlikely related to study treatment, and until the database lock for AEs considered by the Investigator as possibly or probably related to the study treatment.

6.1.7 *Multiple Signs or Symptoms*

If an AE consists of several signs or symptoms that can be represented by one single syndrome or diagnosis, the syndrome or diagnosis will be recorded in the eCRF as the AE instead of the individual signs or symptoms.

6.1.8 *Worsening Signs*

Symptoms, syndromes or diagnoses present before the first administration of the IMP will be considered as AEs if they worsen after the start of the IMP.

6.2 Serious Adverse Events or Reactions

6.2.1 *Definitions*

A Serious Adverse Event (SAE) is any untoward medical occurrence or effect that at any dose:

- results in death;
- is life-threatening (at the time of the event);
- requires in patient hospitalization or prolongation of existing hospitalization;
- results in persistent or significant disability/incapacity;
- is a congenital anomaly/birth defect;
- or is an important medical event.

Death: the death of a patient enrolled in a clinical study is per se not an event, but an outcome. An AE resulting in a fatal outcome must be fully documented and reported, including if the death occurred after treatment end, and regardless of the causality relationship of the death to the IMP. The cause of the death is usually the AE. If the cause cannot be determined, the case will be considered an unexplained death.

Life-threatening: an AE that places the patient, in the view of the initial reporter (Investigator), at immediate risk of death from the AE as it occurred, i.e. it does not include an AE that, had it occurred in a more severe form, might have caused death.

Hospitalization: an AE requiring an unplanned inpatient hospital admission, including at least an overnight stay. Of note: when standard practice for liver biopsy requires overnight hospitalization, this shall not be considered as an adverse event.

Disability: a substantial disruption of a person's ability to conduct normal life functions.

Important medical event: an important medical event that may not result in death, be life-threatening, or require hospitalization may be considered as a SAE when, based upon appropriate medical judgement, it may jeopardize the patient and may require medical or surgical intervention to prevent one of the outcomes listed in the definition. The concept includes AEs which suggest a significant hazard, contraindication or precaution for use, occurrence of malignancy or development of drug dependency or drug abuse.

Overdose: an overdose of an IMP is the accidental or intentional administration of a dose higher than the highest dose under clinical investigation. An overdose will be documented and reported to the safety officer (SO) in the same way as a SAE even if no toxic effects were observed.

Pregnancy: The occurrence of a pregnancy of a study patient or a partner of a study patient discovered during IMP administration or within 1 week after last administration of the IMP, is to be communicated to the Sponsor in an expedited manner with the same procedure and timelines as for SAEs (see section 6.2.2), independently from the occurrence of an AE. Specific follow-up of the event will be requested by the Sponsor until pregnancy outcome, at least on a quarterly basis as described in paragraph 10.1.

6.2.2 *Immediate reporting*

The Investigator will immediately, i.e. within 24 hours from first knowledge, report any SAE occurring during the study to the safety officer (SO) acting on behalf of the Sponsor of the study:

[redacted]

The Investigator will complete in English and sign a SAE Report Form and transmit it to the SO by email or telefax not later than 24 hours after the first knowledge of the SAE. The SO acknowledges the receipt of the SAE information by email to the investigational site within one working day. In the absence of email acknowledging the receipt or in case of issue in sending the fax or email, the Investigator shall contact the SO by any means for ensuring the receipt of SAE information at the earliest opportunity.

Any follow-up information will be reported to the SO and to the Sponsor as soon as it becomes known, with the same process and timelines as described here above for initial reports. As per Article 17 of the European Directive 2001/20/EC, the Sponsor will ensure that all relevant information about SAE and suspected serious unexpected adverse reactions (SUSAR) is recorded and reported to the national CA in all Member States concerned and to Ethics Committee within the applicable timeframe.

Serious adverse events occurring after the last study visit will only be reported if the Investigator believes that the event may have been caused by the IMP or a protocol procedure.

Hospitalization for a diagnosis or therapeutic procedure planned before study enrolment but performed after the enrolment should not be considered for SAE and should be reported in eCRF in concomitant procedures page.

6.3 Safety Variables

All safety variables will be primary ones. Safety evaluations will be completed as given in section 9.7 and as indicated below.

The Investigator will rate all safety variables as to clinical relevance or not. In case of a clinically relevant abnormality, the safety evaluation will be repeated. If the abnormality is confirmed and clinically relevant, it will be recorded as an AE term in the eCRF. The abnormality will be followed up until the value has returned to normal or an adequate explanation is found.

6.3.1 *Physical Examination*

Physical examinations will be conducted by the Investigator. Any findings will be documented in the source document and recorded in the eCRF.

6.3.2 *Laboratory Tests*

Laboratory tests will be analyzed by a central laboratory (CL). In the aim of maintining the "double-blind", some biological values will be blinded on Central laboratory reports/records until the end of the study and could be unblinded only on DSMB members request. In this case, an independent data-management team will be indetify at [redacted] to perform the data-transfert of theses biological value.

Additional samples for laboratory safety variables may be drawn at any time during the study at the Investigators judgement. The laboratory assessments performed at each visit are listed in the following table (see Table 7 and Table 8).

Table 7 : Central laboratory tests for safety assessment

Domain	Goal	Test	Screen	V0	V1	V2	V3	V4
Bone	Safety	B-Crosslaps	-	✓	-	-	✓	-
	Safety	Osteocalcin	-	✓	-	-	✓	-
Chemistry	Safety	Creatinine	✓	✓	✓	✓	✓	✓
	Safety	CPK	✓	✓	✓	✓	✓	✓
	Safety	Urea	✓	✓	✓	✓	✓	✓
	Safety	Albumin	✓	✓	✓	✓	✓	✓
Hematology	Safety	Hb	✓	✓	✓	✓	✓	✓
	Safety	RBC	✓	✓	✓	✓	✓	✓
	Safety	Platelets	✓	✓	✓	✓	✓	✓
	Safety	WBC (Diff Count)	✓	✓	✓	✓	✓	✓
	Safety	MCV	✓	✓	✓	✓	✓	✓
	Safety	CCMH	✓	✓	✓	✓	✓	✓
	Safety	Hematocrit	✓	✓	✓	✓	✓	✓
	Safety	Reticulocytes	✓	✓	✓	✓	✓	✓
Inflammation	Safety	Nt-ProBNP	-	✓	-	-	✓	-
liver	Safety	INR	✓	✓	✓	✓	✓	✓

Table 8 : Central Laboratory tests for efficacy assessment

Domain	Goal	Test	Screen	V0	V1	V2	V3	V4
Fibrosis	Efficacy	TIMP-1	-	✓	-	-	✓	-
	Efficacy	TIMP-2	-	✓	-	-	✓	-
	Efficacy	cytokeratin K18	-	✓	-	-	✓	-
	Efficacy	hyaluronic acid	-	✓	-	-	✓	-
	Efficacy	P3NP	-	✓	-	-	✓	-
	Efficacy	FGF 21	-	✓	-	-	✓	-
Glucids	Efficacy	Insulin	-	✓	-	-	✓	-
	Efficacy	Fasting glucose	✓	✓	✓	✓	✓	✓
	Efficacy	HbA1c	✓	✓	✓	✓	✓	✓
	Efficacy	peptide C	-	✓	-	-	✓	-
	Efficacy	fructosamine	-	✓	-	-	✓	-
Chemistry	Efficacy	Plasma Iron	✓	-	-	-	✓	-
	Efficacy	Transferrin	✓	-	-	-	✓	-
	Efficacy	Ferritin	✓	-	-	-	✓	-
Inflammation	Efficacy	Fibrinogen	-	✓	-	-	✓	-
	Efficacy	hs-CRP	-	✓	-	-	✓	-
	Efficacy	Alpha2-macrogulin	-	✓	-	-	✓	-
	Efficacy	Haptoglobin	-	✓	-	-	✓	-
Lipids	Efficacy	Triglycerides	✓	✓	✓	✓	✓	✓
	Efficacy	HDL-C	✓	✓	✓	✓	✓	✓
	Efficacy	LDL-C	✓	✓	✓	✓	✓	✓
	Efficacy	Total-C	✓	✓	✓	✓	✓	✓
	Efficacy	FFA	-	✓	-	-	✓	-
	Efficacy	APO A1	-	✓	-	-	✓	-
	Efficacy	Adiponectin	-	✓	-	-	✓	-
	Efficacy	Leptin	-	✓	-	-	✓	-
Liver	Efficacy	ALT	✓	✓	✓	✓	✓	✓
	Efficacy	AST	✓	✓	✓	✓	✓	✓
	Efficacy	AlcPhos	✓	✓	✓	✓	✓	✓
	Efficacy	Bilirubin total	✓	✓	✓	✓	✓	✓
	Efficacy	GGT	✓	✓	✓	✓	✓	✓

6.3.3 *Vital Signs*

Vital signs will be assessed at each visit including the screening visit and from V0 to V4. Please refer to section 5.2.6 for the detailed technical procedure. For methods and normal values definition, see Appendix B

6.3.4 *Electrocardiogram*

Please refer to section 5.2.4 for the detailed technical procedure.

6.3.5 *Biobank Samples*

Biobank samples will be taken at the randomization visit (V0) before treatment initiation and at the end-of-treatment visit (V3). The Biobank assessments to be performed are listed in the following table (see Table 9). Based on scientific knowledge at the end of the study, this list might be updated to perform the most appropriate tests in the scope of NASH.

Table 9 : Biobank tests

Domain	Goal	Biomarker name	Screen	V0	V1	V2	V3	V4
Inflammation	Efficacy	IL-13	-	✓	-	-	✓	-
	Efficacy	IL-17A	-	✓	-	-	✓	-
	Efficacy	IL-6		✓			✓	
	Efficacy	IL-1b	-	✓	-	-	✓	-
	Efficacy	TNF-a	-	✓	-	-	✓	-
	Efficacy	INFg	-	✓	-	-	✓	-
Lipids	Efficacy	APO B	-	✓	-	-	✓	-
	Efficacy	APO C3	-	✓	-	-	✓	-
Fibrosis	Efficacy	cytokeratin M30	-	✓	-	-	✓	-
	Efficacy	cytokeratin M65	-	✓	-	-	✓	-
	Efficacy	liver iron content	-	✓	-	-	✓	-
	Efficacy	MMP2	-	✓	-	-	✓	-
	Efficacy	MMP9	-	✓	-	-	✓	-
	Efficacy	Pro-C3	-	✓	-	-	✓	-
Genotype	Other	PNPLA3	-	✓	-	-	-	-
	Other	TM6FS2	-	✓	-	-	-	-

- *Biobank*

Biobank means that the blood and urine samples will be stored and analyzed only at the end of the study according to the efficacy results of the study and the need of further investigations to improve the knowledge of the mechanisms of action of lanifibranor.

All blood and urine samples will be destroyed at the end of the scheduled storage period (1 year after the end of the study), when all analyses will be completed.

DNA sample will be stored up to 1 year after the end of the study for genetic analysis only related to the NASH Condition.

- *Genotype:*

Genetic polymorphism of PNPLA3 and TM6FS2 could be associated to progression from NAFLD to NASH and to liver fibrosis. Genotyping will therefore be performed to assess if variants strongly associated with NAFLD progression may confound treatment effect as well as to characterise the population that is included in this trial.

DNA sample will be stored up to 1 year after the end of the study for genetic analysis only related to NASH.

6.4 Data Safety Monitoring Committee

6.4.1 *Mission*

The DSMB will be responsible for safeguarding the interests of trial participants, assessing safety of the interventions during the trial.

The DSMB will review the protocol and other clinical trial material to ensure that the study will provide useful information regarding the safety of lanifibranor in NASH and that it fulfills ethical requirements.

The DSMB will provide recommendations about continuing or stopping the trial.

The DSMB will be advisory to the steering committee.

6.4.2 *Membership*

The DSMB is an independent multidisciplinary group consisting in clinicians and a biostatistician that, collectively, has experience with the conduct of randomized clinical trials.

The DSMB membership will be restricted to individuals free of apparent significant conflict of interest. The source of these conflicts can be financial, scientific or regulatory in nature. Thus neither study Investigators nor individuals employed by the Sponsor, nor individuals who might have regulatory responsibilities for the trial product, can be members of the DSMB.

6.4.3 *Safety monitoring*

The DSMB will be provided in a semi-unblinded way (treatment A, B, C), with ECG parameters (heart rate, QT, QTc Bazett and Fridericia, delta QTc Bazett and Fridericia, PR interval, ECG interpretation) and main laboratory assessments. In addition, they will be provided with a listing of all AE/SAEs. The DSMB may request a break of the blind, and SUSAR will be provided unblinded.

The frequency of the DSMB and the criteria for ad-hoc meeting will be specified in the DSMB charter.

6.4.4 *Organization*

The first meeting of the DSMB will be an organizational meeting. It will be held during the final stage of protocol development, to provide advisory review of the scientific and ethical issues relating to the study design and conduct, to discuss the standard operating procedures for the role and functioning of the DSMB, and to discuss the format of closed reports that will be used to present trial results at future DSMB meetings.

The organizational meeting will be attended by the DSMB, and by representatives of the Sponsor, the principal Investigator, and the CRO. The DSMB will be provided with draft of the protocol, the current version of the case report form (eCRF screens) and the initial draft of the closed report.

The statistical analysis for the DSMB will be performed by an independent statistical team of the [redacted]. This team will not be co-localised with the team of [redacted] involved the monitoring/data management and project-management activities.The formal analysis meetings will be held to review data every 50 patients for the duration of the trial.

6.4.5 *Procedures to ensure confidentiality and proper communication*

To enhance the integrity and credibility of the trial, procedures will be implemented to ensure that the DSMB has sole access to evolving information from the clinical trial regarding safety data aggregated by treatment arm in a semi-unblinded way, with a possibility of requesting a break of the blind.

Confidential reports for the DSMB will be prepared in an automated way, based on electronic data capture and consolidation of ECG, laboratory data and SAE/SUSAR reports.

The Chair of the DSMB will only communicate to the Trial Steering Committee (TSC) the recommendation to continue or stop the trial. Should there be a need to discuss any other matter the chair of the DSMB will convene a formal meeting with the TSC with a jointly agreed agenda. The trial steering committee will include a representative of each country involved in the study.

6.4.6 *Minutes of the DSMB meetings*

The DSMB will prepare closed minutes of their meeting. The reports will be signed by all three members and originals kept by the chair until completion and archiving of the study.

6.4.7 *Recommendations to the steering committee*

At each meeting of the DSMB during the conduct of the trial, the DSMB will make a recommendation to the TSC to continue or terminate the trial. This recommendation will be based on safety considerations and will be guided by the statistical monitoring guidelines defined in this section.

7. Pharmacokinetic Assessments

Pharmacokinetic sampling will be completed as described as indicated below.

7.1 IMP Concentration Measurements

7.1.1 *Plasma Concentration*

Two pharmacokinetic blood samples (3 ml) will be drawn during the whole study, in the morning just before the drug intake of that day (trough level)

- At V1 (week 4), first interim visit,
- At V3 (week 24), end-of-treatment visit.

The patient will be instructed to take his daily treatment after the samples taken.

The exact times of the last drug intake and the sampling need to be noted in the eCRF.

7.1.2 *Handling and Shipment of Samples*

The kits with all the required supplies are provided by CL.

The whole blood samples (3 mL) for determination of lanifibranor will be collected by venous puncture or indwelling venous catheter into polypropylene tubes containing lithium heparin (Li-Heparin).

Samples will be immediately placed on ice water and subsequently (within 30 minutes of collection) centrifuged at 1760 g and at temperatures between +3°C to +5°C (if possible) otherwise at ambient temperature and for 10 minutes.

For each 3 mL blood sample, two aliquots of exactly 0.5 mL plasma will be rapidly transferred into a screw-capped and stoppered (airtight) polypropylene tube containing 20 µL of orthophosphoric acid H3PO4 2.5 mol/L in order to stabilize the lanifibranor metabolites in plasma.

The plasma samples will be homogenized by hand or with a vortex system and immediately stored at -80°C (if possible), otherwise at -20°C. The samples will be collected by DHL within 48 hours and shipped on dry ice to CL.

7.2 Non-Compartimental Pharmacokinetic analysis

Descriptive statistic of trough levels of lanifibranor will be presented. Individual plasma trough concentration of lanifibranor will be presented in listings. For V1 and V2 individual data of trough lanifibranor plasma concentration will be tabulated with descriptive statistics such as number of valid samples (n), mean, geometric mean, median, SD, minimum, maximum and coefficient of variation. Summary statistics will also be presented separately by gender

8. Data Quality Assurance

8.1 Monitoring

During study conduct, INVENTIVA or its representative will conduct periodic monitoring visits. During monitoring visits, the monitors will verify the adherence to the protocol, the maintenance of all study-related records and the accuracy and completeness of all eCRF entries compared with source data in order to ensure that:

- Data are authentic, accurate, and complete.
- Safety and rights of subjects are being protected.
- Study is conducted in accordance with the currently approved protocol, ICH-GCP and all applicable regulatory requirements

Any discrepancies identified must be resolved. The monitors will review source documents to confirm that the data recorded on eCRF is accurate. The Investigator and institution will allow INVENTIVA or its representative monitors and appropriate regulatory authorities' direct access to source documents to perform the verification.

The study sites may be subject to review by the Institutional Review Board (IRB)/Independent Ethics Committee (IEC), and/or to quality assurance audits performed by INVENTIVA, or companies working with or on behalf of INVENTIVA, and/or to inspection by appropriate regulatory authorities.

It is important that the Investigator(s) and their relevant personnel are available during the monitoring visits and possible audits or inspections and that sufficient time is devoted to the process.

8.2 Data recording

Electronic Data Capture (EDC) will be used for this study, meaning that all data will be entered on an eCRF at the investigational site. This eCRF will be specifically designed for the study and developed by the Data Management Department of [redacted] using Marvin®, a validated Electronic Records/Electronic Signature-compliant (21 CFR Part 11) application provided by the clinical company.

In this clinical trial, only anonymized data will be recorded based on the clinical data collected. The anonymized data of medical information includes the results of the trials tests and will be communicated to the Sponsor or its representative.

Only members of the research team, who also form part of the patient's direct clinical care team, at the participating sites will have access to patient records as part of their medical care and will use these records to screen for potentially eligible patients. Patients who consent to enter the trial are informed that staff from the research team, the Sponsor (and its representatives), relevant regulatory authorities and ethical committees may require access to their full medical records for monitoring and auditing purposes.

After the end of the study (validation of the final report), the data will be totally anonymized and will be stored on a dedicated and secured slot on an Inventiva's server. Only authorized Inventiva employees or its representative will have access to the data.

8.3 Data management

8.3.1 *Responsibilities*

The KBP Monitors and/or Data Managers will provide all tools, instructions, and training necessary to complete the eCRF, and each user will be issued a unique username and password.

The Data Management of KBP will be responsible for data processing, in accordance with the CRO data management procedures.

The Investigators will have to verify that all data entries in the eCRF are accurate and correct. If some assessments are not done, or if certain information is not available, not applicable, or unknown, the Investigators will have to indicate this in the eCRF. The Investigators will be required to electronically sign off the clinical data.

The Monitors will review the eCRFs and evaluate them for completeness and consistency. The eCRF will be compared with the source documents to ensure that there are no discrepancies.

All entries, corrections, and alterations will be made by the Investigator or his/her delegate. The Monitors cannot enter data in the eCRFs.

8.3.2 *Data collection and validation*

All clinical data will be entered in the eCRF at the investigational sites. For each patient of a given site (screen failure and enrolled), an eCRF must be completed in English, by the Investigator or designee, and signed by the Investigator.

A unique subject code will identify the subjects on the eCRF.

Once clinical data of the eCRF have been submitted to the central server, corrections to the data fields will be audit trailed, meaning that the reason for change and the name of the person who performed the change, together with time and date, will be logged. Roles and rights of the site personnel responsible for entering the clinical data into the eCRF will be determined in advance and documented on the "delegation form".

Automatic checks and listings will be designed and performed according to the data validation plan, developed by [redacted] Data Managers and approved by Inventiva. In case of missing values, out of range values, data inconsistencies or values that fail logical checks, queries will be edited in the EDC application. In addition, the Monitors and Data Manager(s) can raise manual queries in the EDC application.

The appropriate investigational staff will answer automatic and manual queries. This will be audit-trailed by the EDC application, meaning that the name of the person who answered and the time and date stamp are captured.

8.3.3 *Data coding*

AEs and Medical History (MH) will be coded using the last version of MedDRA terminology. Concomitant medications will be coded using WHO Drug 2016 Q1 (or a more recent version) terminology.

The medical coding will be performed by [redacted] coding specialist and reviewed by a [redacted] physician before being submitted to Inventiva for approval.

8.3.4 *Database lock*

Once validated, the database will be locked so that no more change will be possible on the frozen data.

After database lock, the Principal Investigator will receive a CD-ROM of the patient data (eCRF data + audit trail) for archiving at the investigational site.

8.3.5 *Database transfer*

Final validated data will be transferred in a secure way to Inventiva and the Biostatistical team in SAS format (SAS datasets format).

8.4 Independent Audit

The Investigator will permit an independent audit by an auditor mandated by the Sponsor, after reasonable notice. An audit or a regulatory inspection is intended to determine if the study was conducted as per protocol (PP), GCP and applicable regulatory requirements, if the rights and well-being of the patients were protected, and if the data relevant for the evaluation of the IMP were captured, processed and reported in compliance with the planned arrangements.

8.5 Regulatory Inspection

Regulatory authorities may perform an inspection of the study including several years after its completion. As for an audit, the Investigator will permit a direct access to all study documents, drug accountability records, source records and source data. If an inspection is announced, the Sponsor will be informed without delay.

9. Statistical Methods

9.1 Efficacy Assessments

Primary endpoint: The primary efficacy endpoint will be a binary variable (responder / non-responder) based on the change from baseline to Week 24 of the SAF activity score. Responder is defined as a decrease from baseline to week 24 of at least 2 points of the SAF activity score combining hepatocellular inflammatory and ballooning without worsening of fibrosis. Any stage increase of fibrosis is considered fibrosis progression. The main question of interest is:

"Does at least one of the two doses increases the percentage of responders compared to placebo?". The main comparison of interest is therefore each of the two verum groups versus placebo.

Secondary endpoints: The following changes from baseline to 24 weeks of treatment will be evaluated:

- Percent of patients with resolution of NASH (Steatosis with or without mild inflammation and no worsening of fibrosis) from baseline to end of treatment (week 24).
- Percent of patients with a change in components of SAF score from baseline to end of treatment (week 24):
    - Steatosis: -1 point
    - Lobular inflammation: -1 point
    - Ballooning: -1 point
- Percent of patients with at least 1-point improvement of fibrosis score on a 4-point scale (SAF) without worsening of NASH, defined as no increase for ballooning, inflammation, or steatosis, using the SAF scoring system, from baseline to end of treatment (week 24).
- Change in fibrosis score on a 4-points scale (SAF) and modified Ishak: - one point from baseline to end of treatment (week 24).
- Liver enzymes (ALT, AST, γGT) change from baseline to end of treatment (week 24).
- Inflammatory markers (fibrinogen, hs-CRP, alpha2 macroglobulin and haptoglobin levels) change from baseline to end of treatment (week 24).
- Glucose metabolism (fasting glucose and insulin, HOMA index and, in subjects with T2DM, HbA1c) change from baseline to end of treatment (week 24).
- Main plasma lipids levels (TC, HDL-C, calculated LDL-C, TG and apoA1) change from baseline to end of treatment (week 24).
- Adiponectin change from baseline to end of treatment (week 24).

Exploratory endpoints:

- Specific markers of inflammation, lipids, glucids, fibrosis, bone remodeling change from baseline to end of treatment (week 24).
- Transient Elastography (TE) and Controlled Attenuation Parameter (CAP) change from baseline to end of treatment (week 24).
- If deemed necessary: Immunohistochemistry: change in the semi quantitative score of ballooning, stellate cell activation, or any other analysis which will be considered relevant in the scope of the study, i.e. to understand the safety and efficacy of lanifibranor in patients with NASH, from baseline to end of treatment (week 24).

9.2 Sample Size

The expected rate of responders according to the retained definition was estimated to 10% for the placebo by a group of clinical expert. An excess rate of responders of 20% was accepted as

clinically pertinent. It corresponds to a multiplication of chance of success (responders) of 3 and a NNT of 5. The sample size required to reach a power of at least 80% is 72 patients per group with a two-sided alpha of 0.025 (adjustment for multiplicity). The sample size is sensitive to the rate of responders in the placebo group. For example, if the rate of responders is 15% instead of 10% then 85 patients are required. If the true rate is lower than 10% then less patients may be sufficient. The sample size is therefore rounded in the conservative side of 75 patients per group.

Based on the experience of previous clinical trials, it is estimated that 100 patients per group will need to be screened to randomize 75 patients after central reading of liver biopsy confirms the NASH diagnosis and activity. In case the end of study liver biopsy slides are not available or not interpretable in more than 5% of patients, an amendment will be submitted to increase sample size accordingly.

9.3 Randomization

Patients will be randomized equally 1:1:1 to either dose of lanifibranor or placebo stratified on presence/absence of diabetes. Strata will be self-weighted *i.e.* patients meeting inclusion/exclusion criteria will be recruited as they come (no quota).

The randomization list will be setup with blocs of size equal to 6.

After identification with a login and a password, the randomization process will be provided through an Interactive Web Response System (IWRS) integrated to the eCRF. Once a set of medical carateristics are entered, the system lets you know immediately, which medication kit to use for this particular patient.

9.4 Protocol Deviations

Protocol deviators will be identified and classified at the blind review. Only patients with major deviations will be discussed in the Clinical Study Report and excluded from the per protocol analysis.

9.5 Data sets Analyzed

- **Full analysis set (FAS)**

The full analysis set consists of all randomized patients who received at least one dose of the assigned treatment. In case of error in the assignment of treatment, the actual treatment will be used in the FAS instead of the treatment assigned in the randomization list.

- **Randomized set of patients**

The randomized set of patients consists of all randomized patients. In case of treatment error, the treatment assigned in the randomization list will prevail over the actual received treatment.

- **Set of evaluable patients**

The set of evaluable patients will consist of the FAS after exclusion of all patients without post-randomization assessments. Concerning the primary endpoint, the absence of post randomization assessment will be considered a failure and the patient will therefore be evaluable if the withdrawal is possibly due to lack of efficacy or safety concern. The same rule will be applied for other binary efficacy variables.

- **Per protocol set of patients**

This set is composed of all patients free from major protocol deviation which can bias the estimate of the treatment effect. Patients with major deviations will be listed during the blind review and the reason for exclusion will be provided.

- **Safety set of patients**

The safety set of patients consists of the FAS who will have taken at least one dose of treatment and who have at least one post-baseline assessment of safety, regardless any protocol deviations. In case of treatment error, the actual treatment will be used.

Safety analyses will be conducted on the safety population which includes all the exposed population, i.e. all patients who will have taken at least one dose of IVA337 and who have at least one post-baseline assessment of safety, regardless any protocol deviations.

9.6 Demographic and Other Baseline Characteristics

9.6.1 *Patient Demographic Characteristics, Medical History and Diagnoses*

Safety characteristics, including demography, medical history, entrance criteria deviations will be summarized using the descriptive statistics (N, mean, standard deviation, minimum and maximum for quantitative values and counts and percents for qualitative variables).

9.6.2 *Previous Medications*

The use of prior medications will be summarized.

9.7 Safety Analysis

The review of safety and tolerance will be performed on the safety population. The safety analysis will be based on the reported AEs and other safety information. The effect of the demographic differences (gender, age, etc.) and risk factors of clinical relevance will be explored.

9.7.1 *Extent of Exposure*

The extent of exposure will be summarized descriptively (N, mean, median, standard deviation and range) using treatment exposure duration. Treatment exposure duration (in days) is defined as: (Date of last dose of study product - First intake of study product date). The distribution and cumulative distribution of patients by treatment exposure duration will be summarized for the safety population.

9.7.2 *Adverse Events*

Each AE will be coded to a "preferred term" and associated "system-organ class" according to an established and validated adverse reaction dictionary (MedDRA). The AE endpoints are number of patients experiencing: - at least one event - an event under each recorded preferred term, - an event under each recorded system-organ class. These endpoints apply to all AEs, regardless of relationship of the event to lanifibranor.

- **Definitions:**

Adverse events will be coded according to Medical Dictionary for Regulatory Affairs (MedDRA) and coded as treatment emergent (TEAE) / non-treatment emergent (non-TEAE) according to the following definitions. TEAEs are defined as events occurring on or after the day of first dose intake of study product and up to follow up visit. Additionally, events present before the first dose of study product, but worsening under treatment are considered as TEAEs. Although

every effort will be made to establish the onset date and time, events with missing onset date will be considered as TEAEs. An AE that will not qualify as a TEAE will be considered as a Non-TEAE. Non-TEAES will be summarized separately from TEAES, and will be presented in the same manner as TEAEs.

- **Treatment Emergent Adverse Events**

A given TEAE will be counted once only per patient. The percentages will be calculated in relation to the population exposed (i.e., safety population). All TEAES will be analyzed, irrespective of their causal relationship with lanifibranor. Recapitulative tables will be provided, showing the following:

- count of exposed patients,
- count and percentage of patients with at least one TEAE,
- count and percentage of each TEAE: TEAEs will be sorted by decreasing order of organ system frequency then by decreasing order of preferred term frequency,
- count and percentage of each TEAE, not taking into account organ system.

In addition, TEAEs will be described according to their time to onset with same categories as for duration of exposure, their maximal intensity and their relationship to lanifibranor, gender and age of patient, time from first intake, outcome, seriousness criteria, corrective treatment, duration, treatment received and action taken. Patient data listings will be provided for all AEs, TEAEs, AEs leading to study discontinuations and SAEs.

Deaths and Serious Adverse Events Incidence of SAEs (irrespective of their emergence classification and relationship to lanifibranor) and deaths will be listed and summarized.

- **Adverse Events leading to treatment discontinuation**

Adverse event leading to discontinuation of the study will be tabulated, irrespective of their emergence classification and irrespective of their relationship to lanifibranor.

9.7.3 *Other Observations Related to Safety*

- **Laboratory tests**

The analysis will include patients exposed to study medication who have at least one laboratory test performed after the first study product intake and, when required by the definition of the abnormality, with an available baseline value and available normal ranges. For these descriptions, the baseline value will be the latest available measure before the first study product dose intake. International units will be used in all listings, tables and graphs. Results and changes from baseline of laboratory parameters will be summarized by mean, standard deviation, median, minimum and maximum at each time point and for the final on-treatment visit. Unscheduled and/or repeated results will only be listed and not summarized.

Shift tables and other tabular and graphical methods will be used to present the results for tests of interest. Listings will be provided with flags indicating clinically significant out-of-range values, clinically non-significant out-of-range values.

In addition, out-of-range values will be assessed by the investigator as clinically significant or not clinically significant. Abnormal laboratory tests assessed by the investigator as clinically significant will be reported as an AE.

- **Vital signs and ECG**

Descriptive statistics by time point will be computed on actual values and changes from baseline for vital signs and ECG data. The baseline value is defined as the last measure before study product intake. ECG parameters collected are heart rate, PR interval, QRS interval, QT, QTcB (Bazett) intervals. Descriptive statistics (means, standard deviations, and ranges) and changes from baseline values for all visits will be provided. The summaries will include patients who have at least one measurement performed after the first study product intake. Results and changes from baseline of the following vital sign parameters will be summarized by mean, standard deviation, median, minimum and maximum at each time point and the final on-treatment visit:

- Heart rate: supine,
- Systolic blood pressure: supine,
- Diastolic blood pressure: supine,
- Weight.

Shift tables and other tabular and graphical methods (plots of mean over time) may be used to present the results for parameters of interest.

Confirmatory–inferential analysis may be done for parameters of interest. Listings will be provided with flags indicating clinically significant out-of-range values, clinically non-significant out-of-range values.

Any ECG modification or change in vital sign compared to screening, considered as clinically significant by the Investigator, will be reported as an AE.

- **Physical examination**

For physical examination anomalies will be listed and frequencies tables will be computed.

When considered clinically relevant by the Investigator, any new finding of the physical examination, compared to the screening visit, will be reported as an AE.

9.7.4 *Efficacy Analyses*

<u>*Primary efficacy analysis*</u>

The primary population will be the intent to treat FAS population composed of all randomized subject who received at least one dose.

The primary efficacy endpoint will be a binary variable (responder / non responder) based on the change from baseline to Week 24 of the SAF activity score. Responder is defined as a decrease from baseline to week 24 of at least 2 points of the SAF activity score combining hepatocellular inflammatory and ballooning without worsening of fibrosis.

The primary <u>tests</u> of hypotheses will be the Cochran Mantel Haenszel test stratified on diabetes comparing the high dose to placebo and the low dose to placebo. The ascending Hochberg procedure will be used for adjusting the type one error which is globally set at 0.05 two-sided. The <u>primary measure</u> of the effect size will be the CMH odds ratio with an adjustment for diabetes. Because the odds ratio is not easy to interpret when the rate of responders in the placebo group is not very small the odds ratio (OR) and its confidence interval will be converted in Risk Ratio (RR) using the following formula $RR = (P_1/P_0) = OR/[(1-P_0) + (P_0 \times OR)]$ (75). The log-binomial model will preferentially be used in the absence of convergence or estimability issue for estimating the effect size with RR.

The primary rule for handling the missing data is to consider non-responders all patients who are not confirmed responders.

The primary handling of centers is to ignore in the primary analysis the center effect because 30+ centers should be involved in the study and the number of empty cells in the breakdown of the site by treatment by stratum table can be large (sparse contingency table).

The primary handling of multiplicity of testing will be done through the Hochberg procedure.

The primary handling of heterogeneity of result across strata will be performed through the Breslow-Day test. If the homogeneity hypothesis is rejected, then the estimate of the difference in percentage points between each dose and placebo along with the 95% C.I. will be calculated within each stratum. If the interaction between stratum and treatment (Breslow Day test) is significant it means that the treatment effect has not the same magnitude in diabetic and non-diabetic patients. In the absence of qualitative interaction there will be no further adjustment of the type one error for multiplicity of testing.

The generalizability of the treatment effect across geographical area will be tested and the statistical strategy will be defined in the SAP once the sample size will be known by stratum and country and before the break of the randomization code.

There is no planned interim analysis for success. However, the DSMB may decide to perform some futility analyses in particular in case of safety concerns.

A series of sensitivity analyses will be performed to assess the robustness of results such as the use of a stratified test (country as a stratification factor), the use of other sets of patients (PP set, Evaluable set, Randomized set, completer set). In case of missing assessment at W24, the patient will be considered a non-responder. A responder is consequently a confirmed responder.

Secondary efficacy analyses:

- NASH improver rates in each dose will be compared to placebo using the Cochran Mantel Haenszel test stratified on diabetes.
- Percent of patients with resolution of NASH in each dose will be compared to placebo using the Cochran Mantel Haenszel test stratified on diabetes.
- Percent of patients with a change in components of SAF score in each dose will be compared to placebo using the Cochran Mantel Haenszel test stratified on diabetes.
- Percent of patients with at least 1-point improvement of fibrosis without worsening of NASH in each dose will be compared to placebo using the Cochran Mantel Haenszel test stratified on diabetes.

Planned exploratory analyses:

- Inflammatory markers (fibrinogen, hs-CRP, alpha2 macroglobulin and haptoglobin levels) change from baseline to end of treatment (week 24) will be compared using GLM with the diabetes and dose as covariates. A mixed model for repeated measures will also be used to compare the time course of the response of each treatment group.
- Transient Elastography and Controlled Attenuation Parameter change from baseline to end of treatment (week 24) will only be described.
- Glucose metabolism (fasting glucose and insulin, HOMA index and, in subjects with T2DM, HbA1c) change from baseline to end of treatment (week 24) will be compared

using GLM with the diabetes and dose as covariates. A mixed model for repeated measures will also be used to compare the time course of the response of each treatment group.

- Main plasma lipids levels (TC, HDL-C, calculated LDL-C, TG and apoA1) change from baseline to end of treatment (week 24) will be compared using GLM with the diabetes and dose as covariates. A mixed model for repeated measures will also be used to compare the time course of the response of each treatment group.
- Adiponectin change from baseline to end of treatment (week 24). Comparison tests and estimates of the difference in the change from baseline will be performed using GLM with the diabetes and dose as covariates. A mixed model for repeated measures will also be used to compare the time course of the response of each treatment group.
- Immunohistochemistry if deemed necessary: change in the semi-quantitative score of ballooning, stellate cell activation, or any other analysis which will be considered relevant in the scope of the study, i.e. to understand the safety and efficacy of lanifibranor in patients with NASH, from baseline to end of treatment (week 24) will be compared using a Van Elteren rank test stratified on diabetes.

The SAP will provide more details on the presented analyses as well as on other exploratory criteria such as quality of life assessment.

All secondary efficacy analyses will be performed using a mixed model for repeated measures or an ANCOVA model if only one post-randomization measure is available. CMH test stratified on diabetes will be used for qualitative variables.

Exploratory analyses on the biomarkers measurements and other evaluations will be proposed and described in the statistical analysis plan.

The final version of the Statistical Analysis Plan will be issued before freezing of the database the break of the randomization code. Any clarification brought in the SAP concerning handling of data and statistical analyses will prevail over any other interpretations of the protocol unless it is the source of major inconsistencies.

10. Patient Withdrawal & Replacement

10.1 Patient Withdrawal

Participation in the study is strictly voluntary. The patients have the right to withdraw from the study at any time for any reason, without the need to justify. The Investigator also has the right to withdraw patients in case of safety concerns, protocol deviations or administrative reasons. Since an excessive rate of withdrawals can render the study uninterpretable, the unnecessary withdrawal of patients must be avoided.

Reasons of withdrawal include, but are not limited, to the following:

- Patient decision
- Lost to follow-up, death
- Any AE, laboratory abnormality or illness which, in the opinion of the Investigator, indicates that continued treatment with study therapy and participation in the trial is not in the best interest of the subject.

- Major protocol deviation: e.g. lack of compliance with scheduled visits, non-compliance with study treatment, treatment with prohibited medication during the study (see 22)
- Any deterioration in cardiac status
- ALT or AST > 8 x ULN; or if ALT or AST > 5xULN for more than 2 weeks; or if ALT or AST > 3xULN and bili (total) > 2xULN (or INR>1.5); or ALT or AST > 3xULN and symptoms (Right Upper Quadrant pain, nausea, …)
- Investigator's decision for any other reason

If a patient discontinues the study, the Investigator will make reasonable efforts to obtain the reason and to perform all protocol-defined end of study assessments as soon as possible. The reason and circumstances for premature discontinuation (e.g. consent withdrawal, AE, lost to follow up, etc.), and the date of withdrawal must be documented in the eCRF. If a patient discontinues the study for safety reasons, the outcome must be known.

In the case of withdrawal due to pregnancy (see paragraph 6.2.1), the patient will be followed until outcome, i.e. at least on a quarterly basis for follow-up evaluation of the pregnancy, foetus, delivery and new born.In the case of withdrawal due to an AE the patient will be followed until resolution of the AE, or until in the opinion of the Investigator the event has stabilized, and the patient is referred to their primary physician for appropriate management of the ongoing event. Reasonable efforts will be made to contact a patient who fails to attend any follow-up appointments, in order to ensure that he/she is in satisfactory health.

Health data and biological samples collected during the whole participation of the patient will be analysed at the end of the study, and the biological samples stored up to one year after the completion of the study and destroyed.

10.2 Unblinding Process

10.2.1 *Unblinding for emergency*

The code breaks will be available 24 hours a day and 7 days a week using an Interactive Web Response System (IWRS) implemented via the eCRF.

The IWRS will require an access code/password/PIN and are only available to staff members named on the delegation log for the trial recorded in the Trial Master file of each site.

The Investigators and delegated members with unblinding responsibilities are responsible for testing their username and password prior to the treatment of subjects to ensure unblinding is possible, or for ensuring appropriately trained staff members are available to action code breaks when required for medical emergencies which may be required out of normal working hours.

Sealed envelopes containing the code-break information will be provided to the Pharmacovigilance and safety Institution ([redacted], see 2.3).

Details of any emergency unblinding shall be documented fully in the Sponsor file, Investigator TMF and Pharmacy and Site File(s). This includes, but may not be limited to:

1) Date,

2) Subject details,

3) Reason for unblinding,

4) The results,

5) Name and role of the individual requesting the unblinding,

6) Name and role of the individual carrying out the unblinding.

If the Clinical Trials Pharmacy or an individual as named on the Delegation Log has performed the procedure, they will inform the Sponsor the trial identifier, subject number and name and title of the person making the request, but NOT the result. The details shall be included in the statistical report.

10.2.2 *Unblinding for DSMB*

Informations will provided to the DSMB unblinded, if requested.

To that effect, a named statistician not involved with the final data analysis or with the study, shall receive the relevant codes. A record shall be kept in the TMF of the name of the statistician, the date they were supplied the relevant code breaks and the location of the results. The unblinded data and the results supplied to the DSMB shall not be accessible by the Investigators or trial staff.

10.2.3 *Unblinding at end of trial*

The Statistical Analysis Plan shall be provided in the protocol or be finalized prior to the release of the randomization codes. Changes to the statistical analysis plan shall be version controlled. A record shall be kept in the Investigator TMF to confirm when the randomization code was requested and when provided.

11. Training & Information

11.1 Training

All persons involved in the study will be trained in an effort to standardize relevant methods, ratings and data capture and to prevent deviations.

11.2 Information of the Investigator

The Investigator will receive all the relevant information for a safe use of the IMP as the study proceeds.

12. Records & Data

12.1 Source Records & Data

Source data are all the information in original records and certified copies of original records of clinical findings, observations, or other activities in the study, which are necessary for the reconstruction and evaluation of the study. The Investigator will permit study-related monitoring, audits, IEC reviews and regulatory inspections, with a direct access to all the required source records. For each patient enrolled, the Investigator will note in the source records that the patient participates in this study, and will record the following information: concomitant therapies, clinically significant adverse events and a statement at patient end of participation.

12.2 Case Report Forms

Electronic Case Report Form (eCRF) will be provided by the CRO institution. The Investigator is responsible for maintaining adequate and accurate data into the eCRF which has been designed to record all observations and other data pertinent to the clinical investigation.

All data requested on the eCRF must be filled out completely by the Investigator or the Investigator team. All data captured for the study is planned to be electronic. The eCRF should be reviewed and electronically approved by the Investigator.

All missing data must be explained. If any entry error has been made, to correct such an error, enter the correct data above it. All such changes will be must electronically signed with the reason for the correction if necessary, by an authorized (Investigator/co-worker) person.

eCRF and all other source data must be easily accessible for review during the programmed monitoring visits. Once the clinical monitor has verified the contents of the completed eCRF against the source data, the system will be verified electronically by the clinical monitor for those pages. Queries may be raised if the data are unclear or contradictory, which must be addressed by the Investigator.

Patients are not to be identified in the eCRF by name or initials and birth date. Appropriate coded identification, e.g. patient number in combination with year of birth must be used.

13. Confidentiality

13.1 Confidentiality of Patient Data

The Investigator will ensure that the confidentiality of the patients' data is preserved. On eCRFs or any other documents for the Sponsor, the patients will not be identified by their names, but by their initials and numbers in the study. Documents not for transmission to the Sponsor, e.g. the confidential patient identification list and the signed informed consent forms will be maintained by the Investigator in strict confidence.

13.2 Confidentiality of Sponsor Data

Any information on the IMP, this study and its results is confidential information. The Investigator, study site personnel and IEC members will not use, publish, or otherwise disclose any confidential information without the prior written authorization from the Sponsor. Any data, results, inventions and patents that may arise from this study will be the exclusive property of the Sponsor.

14. Reporting & Publication

14.1 Study Report

All the relevant data and information will be reported in a study report prepared by CRO ([redacted], section 2.2) and submitted to the Sponsor and the Investigators for review comments and signature. The final study report will be used for the further development of the IMP and regulatory submissions.

14.2 Disclosure of Data and Publications

No information provided by INVENTIVA S.A. to the Investigators for the purposes of performing the study, will be published, or passed on to a third party, without prior written approval by INVENTIVA.

The Investigators will have full access to all of the study data and will take complete responsibility for the integrity of the data and the relevance of the data analysis and reporting.

After regulatory clearance, the study will be registered by the Sponsor in the ClinTrials.gov database. The Principal Investigator or anyone else working on the study will submit all proposed publications, papers, abstracts or other written materials or an outline of any proposed oral presentation related to the study to INVENTIVA S.A. at least 1 month prior to (i) submission of such written materials for publication, or (ii) any proposed oral disclosure to a third party. INVENTIVA S.A. shall have the right to comment on such written material/outline and to take any necessary action to protect its intellectual property; the Principal Investigator, in determining the final form of disclosure, shall consider such comments in good faith. Notwithstanding any of the above, the Principal Investigator or anyone else working on the Study may not include any confidential information unrelated to the study in any such publication or disclosure.

The Investigator will provide INVENTIVA S.A. with complete test results and all data derived from the study in accordance with the protocol.

Only INVENTIVA S.A. and its authorized contractor may make information obtained during the study available to regulatory agencies, except as required by regulation.

15. Record Keeping

15.1 Study Site Records

The Investigator is responsible for maintaining all the records which enable the conduct of the study at the study site to be fully understood. The study documentation and source records will be archived for the maximum period of time permitted by local requirements. The Investigator will complete the confidential patient identification list which provides the sole link between source records and anonymous eCRF data. The Investigator will retain this confidential list and the signed consent forms for at least fifteen years after the completion or discontinuation of the study. No study site records may be destroyed without prior written agreement between the investigator and the Sponsor. If the Investigator intends to assign the study documents to another party, or to move them to another location, the Sponsor must be notified.

15.2 Study Master File

The Sponsor will archive the study master file in accordance with GCP and applicable regulatory requirements.

Bibliography

1. Farrell GC LC. Nonalcoholic fatty liver disease: from steatosis to cirrhosis. Hepatology. 2006;43:S99–S112.

2. Ratziu V, Poynard T. Assessing the outcome of nonalcoholic steatohepatitis? It's time to get serious. Hepatology. 2006 Oct;44(4):802–805.

3. Ratziu V, Giral P, Charlotte F, Bruckert E, Thibault V, Theodorou I, et al. Liver fibrosis in overweight patients. Gastroenterology. 2000 Jun;118(6):1117–1123.

4. Ratziu V, Poynard T. NASH: a hidden and silent fibroser finally revealed? J Hepatol. 2005 Jan;42(1):12–14.

5. Ratziu V, Bonyhay L, Martino VD, Charlotte F, Cavallaro L, Sayegh-Tainturier M-H, et al. Survival, liver failure, and hepatocellular carcinoma in obesity-related cryptogenic cirrhosis. Hepatology. 2002;35:1485–93.

6. Sanyal AJ, Banas C, Sargeant C, Luketic VA, Sterling RK, Stravitz RT, et al. Similarities and differences in outcomes of cirrhosis due to nonalcoholic steatohepatitis and hepatitis C. Hepatology. 2006 Apr;43(4):682–689.

7. Targher G. Non-alcoholic fatty liver disease, the metabolic syndrome and the risk of cardiovascular disease: the plot thickens. Diabet Med. 2007 Jan;24(1):1–6.

8. Francque SM, van der Graaff D, Kwanten WJ. Non-alcoholic fatty liver disease and cardiovascular risk: Pathophysiological mechanisms and implications. J Hepatol [Internet]. 2016 Jun [cited 2016 Jun 7]; Available from: http://linkinghub.elsevier.com/retrieve/pii/S0168827816301076

9. Ekstedt M, Franz�n LE, Mathiesen UL, Thorelius L, Holmqvist M, Bodemar G, et al. Long-term follow-up of patients with NAFLD and elevated liver enzymes. Hepatology. 2006 Oct;44(4):865–873.

10. Adams LA, Lymp JF, Sauver JS, Sanderson SO, Lindor KD, Feldstein A, et al. The natural history of nonalcoholic fatty liver disease: a population-based cohort study. Gastroenterology. 2005 Jul;129(1):113–121.

11. Bugianesi E, Leone N, Vanni E, Marchesini G, Brunello F, Carucci P, et al. Expanding the natural history of nonalcoholic steatohepatitis: from cryptogenic cirrhosis to hepatocellular carcinoma. Gastroenterology. 2002 Jul;123(1):134–140.

12. Ratziu V, Vispo M, Tahiri M, Bonyhay L, Torres M de, Bernhardt C, et al. Can metabolic steatohepatitis be treated ? Gastroenterol Clin Biol. 2007 Mar;31(3):333–340.

13. Vilar-Gomez E, Martinez-Perez Y, Calzadilla-Bertot L, Torres-Gonzalez A, Gra-Oramas B, Gonzalez-Fabian L, et al. Weight Loss Through Lifestyle Modification Significantly Reduces Features of Nonalcoholic Steatohepatitis. Gastroenterology. 2015 Aug;149(2):367–378.

14. Belfort R, Harrison SA, Brown K, Darland C, Finch J, Hardies J, et al. A placebo-controlled trial of pioglitazone in subjects with nonalcoholic steatohepatitis. N Engl J Med. 2006 Nov;355(22):2297–2307.

15. Bugianesi E, Gentilcore E, Manini R, Natale S, Vanni E, Villanova N, et al. A randomized controlled trial of metformin versus vitamin E or prescriptive diet in nonalcoholic fatty liver disease. Am J Gastroenterol. 2005 May;100(5):1082–1090.

16. Ratziu V, Giral P, Jacqueminet S, Charlotte F, Hartemann-Heurtier A, Serfaty L, et al. Rosiglitazone for nonalcoholic steatohepatitis: one-year results of the randomized placebo-controlled Fatty Liver Improvement with Rosiglitazone Therapy (FLIRT) Trial. Gastroenterology. 2008 Jul;135(1):100–110.

17. Musso G, Gambino R, Tabibian JH, Ekstedt M, Kechagias S, Hamaguchi M, et al. Association of non-alcoholic fatty liver disease with chronic kidney disease: a systematic review and meta-analysis. PLoS Med. 2014 juillet;11(7):e1001680.

18. Pavlides M, Banerjee R, Tunnicliffe EM, Kelly C, Collier J, Wang LM, et al. Multiparametric magnetic resonance imaging for the assessment of non-alcoholic fatty liver disease severity. Liver Int Off J Int Assoc Study Liver. 2017 Jul;37(7):1065–73.

19. Day CP, James OF. Steatohepatitis: a tale of two “hits”? Gastroenterology. 1998 Apr;114(4):842–845.

20. Sanyal AJ, Campbell-Sargent C, Mirshahi F, Rizzo WB, Contos MJ, Sterling RK, et al. Nonalcoholic steatohepatitis: association of insulin resistance and mitochondrial abnormalities. Gastroenterology. 2001 Apr;120(5):1183–1192.

21. Poulsen L la C, Siersbæk M, Mandrup S. PPARs: Fatty acid sensors controlling metabolism. Semin Cell Dev Biol. 2012 Aug;23(6):631–9.

22. Feige JN, Gelman L, Michalik L, Desvergne B, Wahli W. From molecular action to physiological outputs: Peroxisome proliferator-activated receptors are nuclear receptors at the crossroads of key cellular functions. Prog Lipid Res. 2006 Mar;45(2):120–59.

23. Lefebvre P. Sorting out the roles of PPAR in energy metabolism and vascular homeostasis. J Clin Invest. 2006 Mar 1;116(3):571–80.

24. Zambon A. Modulation of Hepatic Inflammatory Risk Markers of Cardiovascular Diseases by PPAR- Activators: Clinical and Experimental Evidence. Arterioscler Thromb Vasc Biol. 2006 Feb 9;26(5):977–86.

25. Lee C-H, Olson P, Hevener A, Mehl I, Chong L-W, Olefsky JM, et al. PPARδ regulates glucose metabolism and insulin sensitivity. Proc Natl Acad Sci U S A. 2006;103(9):3444–3449.

26. Tailleux A, Wouters K, Staels B. Roles of PPARs in NAFLD: Potential therapeutic targets. Biochim Biophys Acta BBA - Mol Cell Biol Lipids. 2012 May;1821(5):809–18.

27. Odegaard JI, Ricardo-Gonzalez RR, Red Eagle A, Vats D, Morel CR, Goforth MH, et al. Alternative M2 Activation of Kupffer Cells by PPARδ Ameliorates Obesity-Induced Insulin Resistance. Cell Metab. 2008 Jun;7(6):496–507.

28. Lalloyer F, Staels B. Fibrates, Glitazones, and Peroxisome Proliferator-Activated Receptors. Arterioscler Thromb Vasc Biol. 2010 May 1;30(5):894–9.

29. Vanni E, Bugianesi E, Kotronen A, De Minicis S, Yki-Järvinen H, Svegliati-Baroni G. From the metabolic syndrome to NAFLD or vice versa? Dig Liver Dis. 2010 May;42(5):320–30.

30. Rockey DC. Current and Future Anti-Fibrotic Therapies for Chronic Liver Disease. Clin Liver Dis. 2008 Nov;12(4):939–62.

31. Peroxisome proliferator-activated receptor-γ as a therapeutic target for hepatic fibrosis: from bench to bedside. - PubMed - NCBI [Internet]. [cited 2016 Feb 11]. Available from: http://www.ncbi.nlm.nih.gov/pubmed/?term=zhang+kong+lu+PPAR#

32. Sanyal AJ, Chalasani N, Kowdley KV, McCullough A, Diehl AM, Bass NM, et al. Pioglitazone, vitamin E, or placebo for nonalcoholic steatohepatitis. N Engl J Med. 2010;362(18):1675–1685.

33. Francque S, Verrijken A, Caron S, Prawitt J, Paumelle R, Derudas B, et al. PPARÎ± gene expression correlates with severity and histological treatment response in patients with non-alcoholic steatohepatitis. J Hepatol. 2015 Jul;63(1):164–173.

34. Ip E. Central role of PPARα-dependent hepatic lipid turnover in dietary steatohepatitis in mice. Hepatology. 2003 Jul;38(1):123–32.

35. Ip E, Farrell G, Hall P, Robertson G, Leclercq I. Administration of the potent PPAR? agonist, Wy-14,643, reverses nutritional fibrosis and steatohepatitis in mice. Hepatology. 2004 May;39(5):1286–96.

36. Shiri-Sverdlov R, Wouters K, Gorp PJ van, Gijbels MJ, Noel B, Buffat L, et al. Early diet-induced non-alcoholic steatohepatitis in APOE2 knock-in mice and its prevention by fibrates. J Hepatol. 2006 Apr;44(4):732–41.

37. Nagasawa T, Inada Y, Nakano S, Tamura T, Takahashi T, Maruyama K, et al. Effects of bezafibrate, PPAR pan-agonist, and GW501516, PPARδ agonist, on development of steatohepatitis in mice fed a methionine- and choline-deficient diet. Eur J Pharmacol. 2006 Apr;536(1–2):182–91.

38. Shan W, Nicol CJ, Ito S, Bility MT, Kennett MJ, Ward JM, et al. Peroxisome proliferator-activated receptor-β/δ protects against chemically induced liver toxicity in mice. Hepatology. 2007 Nov 25;47(1):225–35.

39. Adenovirus-mediated peroxisome proliferator activated receptor gamma overexpression prevents nutritional fibrotic steatohepatitis in mice. - PubMed - NCBI [Internet]. [cited 2016 Feb 11]. Available from: http://www.ncbi.nlm.nih.gov/pubmed/20969493

40. PPARgamma is essential for protection against nonalcoholic steatohepatitis. - PubMed - NCBI [Internet]. [cited 2016 Feb 11]. Available from: http://www.ncbi.nlm.nih.gov/pubmed/20376096

41. Yu J, Zhang S, Chu ESH, Go MYY, Lau RHY, Zhao J, et al. Peroxisome proliferator-activated receptors gamma reverses hepatic nutritional fibrosis in mice and suppresses activation of hepatic stellate cells in vitro. Int J Biochem Cell Biol. 2010 Jun;42(6):948–57.

42. Rosiglitazone attenuates age- and diet-associated nonalcoholic steatohepatitis in male low-density lipoprotein receptor knockout mice. - PubMed - NCBI [Internet]. [cited 2016 Feb 11]. Available from: http://www.ncbi.nlm.nih.gov/pubmed/20938947

43. Leclercq IA. Limited therapeutic efficacy of pioglitazone on progression of hepatic fibrosis in rats. Gut. 2006 Feb 16;55(7):1020–9.

44. Hsiao P-J, Hsieh T-J, Kuo K-K, Hung W-W, Tsai K-B, Yang C-H, et al. Pioglitazone retrieves hepatic antioxidant DNA repair in a mice model of high fat diet. BMC Mol Biol. 2008;9(1):82.

45. Zhang W, Xu Y-Z, Liu B, Wu R, Yang Y-Y, Xiao X-Q, et al. Pioglitazone Upregulates Angiotensin Converting Enzyme 2 Expression in Insulin-Sensitive Tissues in Rats with High-Fat Diet-Induced Nonalcoholic Steatohepatitis. Sci World J. 2014;2014:1–7.

46. Leclercq IA, Lebrun VA, Stärkel P, Horsmans YJ. Intrahepatic insulin resistance in a murine model of steatohepatitis: effect of PPARγ agonist pioglitazone. Lab Invest. 2007;87(1):56–65.

47. Hazra S. Peroxisome Proliferator-activated Receptor Induces a Phenotypic Switch from Activated to Quiescent Hepatic Stellate Cells. J Biol Chem. 2004 Jan 26;279(12):11392–401.

48. Marra F, Efsen E, Romanelli RG, Caligiuri A, Pastacaldi S, Batignani G, et al. Ligands of peroxisome proliferator-activated receptor γ modulate profibrogenic and proinflammatory actions in hepatic stellate cells. Gastroenterology. 2000 Aug;119(2):466–78.

49. Galli A, Crabb D, Price D, Ceni E, Salzano R, Surrenti C, et al. Peroxisome proliferator-activated receptor γ transcriptional regulation is involved in platelet-derived growth factor–induced proliferation of human hepatic stellate cells. Hepatology. 2000;31(1):101–108.

50. Miyahara T, Schrum L, Rippe R, Xiong S, Yee HF, Motomura K, et al. Peroxisome Proliferator-activated Receptors and Hepatic Stellate Cell Activation. J Biol Chem. 2000 Nov 17;275(46):35715–22.

51. Staels B, Rubenstrunk A, Noel B, Rigou G, Delataille P, Millatt LJ, et al. Hepatoprotective effects of the dual peroxisome proliferator-activated receptor alpha/delta agonist, GFT505, in rodent models of nonalcoholic fatty liver disease/nonalcoholic steatohepatitis. Hepatology. 2013 Dec;58(6):1941–1952.

52. Iwaisako K, Haimerl M, Paik Y-H, Taura K, Kodama Y, Sirlin C, et al. Protection from liver fibrosis by a peroxisome proliferator-activated receptor agonist. Proc Natl Acad Sci. 2012 May 22;109(21):E1369–76.

53. Ratziu V, Harrison S, Francque S, Bedossa P, Lehert P, Serfaty L, et al. Elafibranor, an Agonist of the Peroxisome Proliferator−Activated Receptor−α and −δ, Induces Resolution of Nonalcoholic Steatohepatitis Without Fibrosis Worsening. Gastroenterology [Internet]. 2016 Feb [cited 2016 Mar 26]; Available from: http://linkinghub.elsevier.com/retrieve/pii/S0016508516001402

54. Sanyal AJ, Brunt EM, Kleiner DE, Kowdley KV, Chalasani N, Lavine JE, et al. Endpoints and clinical trial design for nonalcoholic steatohepatitis. Hepatology. 2011 juillet;54(1):344–353.

55. Sanyal AJ, Friedman SL, McCullough AJ, Dimick-Santos L. Challenges and opportunities in drug and biomarker development for nonalcoholic steatohepatitis: Findings and recommendations from an American Association for the Study of Liver Diseases-U.S. Food and Drug Administration Joint Workshop: SANYAL ET AL. Hepatology. 2015 Apr;61(4):1392–405.

56. Cadranel J-F, Rufat P, Degos F, for the Group of Epidemiology of the French Association for the Study of the Liver. Practices of Liver Biopsy in France: Results of a Prospective Nationwide Survey. Hepatology. 2000 Sep;32(3):477–81.

57. Takyar V, Etzion O, Heller T, Kleiner DE, Rotman Y, Ghany MG, et al. Complications of percutaneous liver biopsy with Klatskin needles: a 36-year single-centre experience. Aliment Pharmacol Ther. 2017 Mar;45(5):744–53.

58. Firpi RJ, Soldevila-Pico C, Abdelmalek MF, Morelli G, Judah J, Nelson DR. Short recovery time after percutaneous liver biopsy: should we change our current practices? Clin Gastroenterol Hepatol Off Clin Pract J Am Gastroenterol Assoc. 2005 Sep;3(9):926–9.

59. Van Der Poorten D, Kwok A, Lam T, Ridley L, Jones DB, Ngu MC, et al. Twenty-year audit of percutaneous liver biopsy in a major Australian teaching hospital. Intern Med J. 2006 Nov;36(11):692–9.

60. Myers RP, Fong A, Shaheen AAM. Utilization rates, complications and costs of percutaneous liver biopsy: a population-based study including 4275 biopsies: Population-based study of percutaneous liver biopsy. Liver Int. 2008 Apr 10;28(5):705–12.

61. West J, Card TR. Reduced Mortality Rates Following Elective Percutaneous Liver Biopsies. Gastroenterology. 2010 Oct;139(4):1230–7.

62. Sanyal AJ, Brunt EM, Kleiner DE, Kowdley KV, Chalasani N, Lavine JE, et al. Endpoints and clinical trial design for nonalcoholic steatohepatitis. Hepatology. 2011 Jul;54(1):344–353.

63. Bedossa P, Poitou C, Veyrie N, Bouillot JL, Basdevant A, Paradis V, et al. Histopathological algorithm and scoring system for evaluation of liver lesions in morbidly obese patients. Hepatology. 2012 Nov;56(5):1751–1759.

64. Bedossa P, Bedossa P, Burt AD, Gouw AS, Lackner C, Schirmacher P, et al. Utility and appropriateness of the fatty liver inhibition of progression (FLIP) algorithm and steatosis, activity, and fibrosis (SAF) score in the evaluation of biopsies of nonalcoholic fatty liver disease. Hepatology. 2014 Aug;60(2):565–575.

65. Kleiner DE, Brunt EM, Natta MV, Behling C, Contos MJ, Cummings OW, et al. Design and validation of a histological scoring system for nonalcoholic fatty liver disease. Hepatology. 2005 juin;41(6):1313–1321.

66. Marchesini G. EASL–EASD–EASO Clinical Practice Guidelines for the management of non-alcoholic fatty liver disease. J Hepatol [Internet]. 2016; Available from: http://dx.doi.org/10.1016/j.jhep.2015.11.004

67. Chalasani N, Younossi Z, Lavine JE, Diehl AM, Brunt EM, Cusi K, et al. The Diagnosis and Management of Non-alcoholic Fatty Liver Disease: Practice Guideline by the American Gastroenterological Association, American Association for the Study of Liver Diseases, and American College of Gastroenterology. Gastroenterology. 2012 Jun;142(7):1592–609.

68. Rockey DC, Caldwell SH, Goodman ZD, Nelson RC, Smith AD. Liver biopsy. Hepatology. 2009 Mar;49(3):1017–44.

69. EASL-ALEH Clinical Practice Guidelines: Non-invasive tests for evaluation of liver disease severity and prognosis. J Hepatol. 2015 Jul;63(1):237–64.

70. de Lédinghen V, Hiriart J-B, Vergniol J, Merrouche W, Bedossa P, Paradis V. Controlled Attenuation Parameter (CAP) with the XL Probe of the Fibroscan®: A Comparative Study with the M Probe and Liver Biopsy. Dig Dis Sci. 2017 Sep;62(9):2569–77.

71. Sasso M, Beaugrand M, de Ledinghen V, Douvin C, Marcellin P, Poupon R, et al. Controlled Attenuation Parameter (CAP): A Novel VCTE™ Guided Ultrasonic Attenuation Measurement for the Evaluation of Hepatic Steatosis: Preliminary Study and Validation in a Cohort of Patients with Chronic Liver Disease from Various Causes. Ultrasound Med Biol. 2010 Nov;36(11):1825–35.

72. Mikolasevic I, Orlic L, Franjic N, Hauser G, Stimac D, Milic S. Transient elastography (FibroScan ®) with controlled attenuation parameter in the assessment of liver steatosis and

fibrosis in patients with nonalcoholic fatty liver disease - Where do we stand? World J Gastroenterol. 2016;22(32):7236.

73. Karlas T, Petroff D, Sasso M, Fan J-G, Mi Y-Q, de Lédinghen V, et al. Individual patient data meta-analysis of controlled attenuation parameter (CAP) technology for assessing steatosis. J Hepatol. 2017 May;66(5):1022–30.

74. Pavlides M, Banerjee R, Tunnicliffe EM, Kelly C, Collier J, Wang LM, et al. Multiparametric magnetic resonance imaging for the assessment of non-alcoholic fatty liver disease severity. Liver Int. 2017 Jul;37(7):1065–73.

75. Zhang J, Yu KF. What's the relative risk? A method of correcting the odds ratio in cohort studies of common outcomes. JAMA. 1998 Nov 18;280(19):1690–1.

76. Kleiner DE, Brunt EM, Natta MV, Behling C, Contos MJ, Cummings OW, et al. Design and validation of a histological scoring system for nonalcoholic fatty liver disease. Hepatology. 2005 Jun;41(6):1313–1321.

APPENDICES

Appendix A: World medical association declaration of Helsinki 79
Appendix B: Methods and normal values for vital signs, waist and ECG 87
Appendix C: Liver Biopsy and histology methods 90
Appendix D : SF-36 Questionnary 95
Appendix E : The Flinders Fatigue Scale (FFS) 100

APPENDIX A: WORLD MEDICAL ASSOCIATION DECLARATION OF HELSINKI

WMA

WMA Declaration of Helsinki - Ethical Principles for Medical Research Involving Human Subjects

Adopted by the 18th WMA General Assembly, Helsinki, Finland, June 1964
and amended by the:
29th WMA General Assembly, Tokyo, Japan, October 1975
35th WMA General Assembly, Venice, Italy, October 1983
41st WMA General Assembly, Hong Kong, September 1989
48th WMA General Assembly, Somerset West, Republic of South Africa, October 1996
52nd WMA General Assembly, Edinburgh, Scotland, October 2000
53rd WMA General Assembly, Washington DC, USA, October 2002 (Note of Clarification added)
55th WMA General Assembly, Tokyo, Japan, October 2004 (Note of Clarification added)
59th WMA General Assembly, Seoul, Republic of Korea, October 2008
64th WMA General Assembly, Fortaleza, Brazil, October 2013

Preamble

1. The World Medical Association (WMA) has developed the Declaration of Helsinki as a statement of ethical principles for medical research involving human subjects, including research on identifiable human material and data.

The Declaration is intended to be read as a whole and each of its constituent paragraphs should be applied with consideration of all other relevant paragraphs.

2. Consistent with the mandate of the WMA, the Declaration is addressed primarily to physicians. The WMA encourages others who are involved in medical research involving human subjects to adopt these principles.

General Principles

3. The Declaration of Geneva of the WMA binds the physician with the words,

“The health of my patient will be my first consideration,” and the International Code of Medical Ethics declares that, “A physician shall act in the patient's best interest when providing medical care.”

4. It is the duty of the physician to promote and safeguard the health, well-being and rights of patients, including those who are involved in medical research. The physician's knowledge and conscience are dedicated to the fulfilment of this duty.

5. Medical progress is based on research that ultimately must include studies involving human subjects.

6. The primary purpose of medical research involving human subjects is to understand the causes, development and effects of diseases and improve preventive, diagnostic and therapeutic interventions (methods, procedures and treatments). Even the best proven interventions must be evaluated continually through research for their safety, effectiveness, efficiency, accessibility and quality.

7. Medical research is subject to ethical standards that promote and ensure respect for all human subjects and protect their health and rights.

8. While the primary purpose of medical research is to generate new knowledge, this goal can never take precedence over the rights and interests of individual research subjects.

9. It is the duty of physicians who are involved in medical research to protect the life, health, dignity, integrity, right to self-determination, privacy, and confidentiality of personal information of research subjects. The responsibility for the protection of research subjects must always rest with the physician or other health care professionals and never with the research subjects, even though they have given consent.

10. Physicians must consider the ethical, legal and regulatory norms and standards for research involving human subjects in their own countries as well as applicable international norms and standards. No national or international ethical, legal or regulatory requirement should reduce or eliminate any of the protections for research subjects set forth in this Declaration.

11. Medical research should be conducted in a manner that minimises possible harm to the environment.

12. Medical research involving human subjects must be conducted only by

individuals with the appropriate ethics and scientific education, training and qualifications. Research on patients or healthy volunteers requires the supervision of a competent and appropriately qualified physician or other health care professional.

13. Groups that are underrepresented in medical research should be provided appropriate access to participation in research.

14. Physicians who combine medical research with medical care should involve their patients in research only to the extent that this is justified by its potential preventive, diagnostic or therapeutic value and if the physician has good reason to believe that participation in the research study will not adversely affect the health of the patients who serve as research subjects.

15. Appropriate compensation and treatment for subjects who are harmed as a result of participating in research must be ensured.

Risks, Burdens and Benefits

16. In medical practice and in medical research, most interventions involve risks and burdens.

Medical research involving human subjects may only be conducted if the importance of the objective outweighs the risks and burdens to the research subjects.

17. All medical research involving human subjects must be preceded by careful assessment of predictable risks and burdens to the individuals and groups involved in the research in comparison with foreseeable benefits to them and to other individuals or groups affected by the condition under investigation.

Measures to minimise the risks must be implemented. The risks must be continuously monitored, assessed and documented by the researcher.

18. Physicians may not be involved in a research study involving human subjects unless they are confident that the risks have been adequately assessed and can be satisfactorily managed.

When the risks are found to outweigh the potential benefits or when there is conclusive proof of definitive outcomes, physicians must assess whether to continue, modify or immediately stop the study.

Vulnerable Groups and Individuals

19. Some groups and individuals are particularly vulnerable and may have an increased likelihood of being wronged or of incurring additional harm.

All vulnerable groups and individuals should receive specifically considered protection.

20. Medical research with a vulnerable group is only justified if the research is responsive to the health needs or priorities of this group and the research cannot be carried out in a non-vulnerable group. In addition, this group should stand to benefit from the knowledge, practices or interventions that result from the research.

Scientific Requirements and Research Protocols

21. Medical research involving human subjects must conform to generally accepted scientific principles, be based on a thorough knowledge of the scientific literature, other relevant sources of information, and adequate laboratory and, as appropriate, animal experimentation. The welfare of animals used for research must be respected.

22. The design and performance of each research study involving human subjects must be clearly described and justified in a research protocol.

The protocol should contain a statement of the ethical considerations involved and should indicate how the principles in this Declaration have been addressed. The protocol should include information regarding funding, sponsors, institutional affiliations, potential conflicts of interest, incentives for subjects and information regarding provisions for treating and/or compensating subjects who are harmed as a consequence of participation in the research study.

In clinical trials, the protocol must also describe appropriate arrangements for post-trial provisions.

Research Ethics Committees

23. The research protocol must be submitted for consideration, comment, guidance and approval to the concerned research ethics committee before the study begins. This committee must be transparent in its functioning, must be independent of the researcher, the sponsor and any other undue influence and must be duly qualified. It must take into consideration the laws and regulations of the country or countries in which the research is to be performed as well as applicable international norms and

standards but these must not be allowed to reduce or eliminate any of the protections for research subjects set forth in this Declaration.

The committee must have the right to monitor ongoing studies. The researcher must provide monitoring information to the committee, especially information about any serious adverse events. No amendment to the protocol may be made without consideration and approval by the committee. After the end of the study, the researchers must submit a final report to the committee containing a summary of the study's findings and conclusions.

Privacy and Confidentiality

24. Every precaution must be taken to protect the privacy of research subjects and the confidentiality of their personal information.

Informed Consent

25. Participation by individuals capable of giving informed consent as subjects in medical research must be voluntary. Although it may be appropriate to consult family members or community leaders, no individual capable of giving informed consent may be enrolled in a research study unless he or she freely agrees.

26. In medical research involving human subjects capable of giving informed consent, each potential subject must be adequately informed of the aims, methods, sources of funding, any possible conflicts of interest, institutional affiliations of the researcher, the anticipated benefits and potential risks of the study and the discomfort it may entail, post-study provisions and any other relevant aspects of the study. The potential subject must be informed of the right to refuse to participate in the study or to withdraw consent to participate at any time without reprisal. Special attention should be given to the specific information needs of individual potential subjects as well as to the methods used to deliver the information.

After ensuring that the potential subject has understood the information, the physician or another appropriately qualified individual must then seek the potential subject's freely-given informed consent, preferably in writing. If the consent cannot be expressed in writing, the non-written consent must be formally documented and witnessed.

All medical research subjects should be given the option of being informed about the general outcome and results of the study.

27. When seeking informed consent for participation in a research study the physician must be particularly cautious if the potential subject is in a dependent relationship with the physician or may consent under duress. In such situations the informed consent must be sought by an appropriately qualified individual who is completely independent of this relationship.

28. For a potential research subject who is incapable of giving informed consent, the physician must seek informed consent from the legally authorised representative. These individuals must not be included in a research study that has no likelihood of benefit for them unless it is intended to promote the health of the group represented by the potential subject, the research cannot instead be performed with persons capable of providing informed consent, and the research entails only minimal risk and minimal burden.

29. When a potential research subject who is deemed incapable of giving informed consent is able to give assent to decisions about participation in research, the physician must seek that assent in addition to the consent of the legally authorised representative. The potential subject's dissent should be respected.

30. Research involving subjects who are physically or mentally incapable of giving consent, for example, unconscious patients, may be done only if the physical or mental condition that prevents giving informed consent is a necessary characteristic of the research group. In such circumstances the physician must seek informed consent from the legally authorised representative. If no such representative is available and if the research cannot be delayed, the study may proceed without informed consent provided that the specific reasons for involving subjects with a condition that renders them unable to give informed consent have been stated in the research protocol and the study has been approved by a research ethics committee. Consent to remain in the research must be obtained as soon as possible from the subject or a legally authorised representative.

31. The physician must fully inform the patient which aspects of their care are related to the research. The refusal of a patient to participate in a study or the patient's decision to withdraw from the study must never adversely affect the patient-physician relationship.

32. For medical research using identifiable human material or data, such as research on material or data contained in biobanks or similar repositories, physicians must seek informed consent for its collection, storage and/or reuse. There may be exceptional situations where consent would be impossible or impracticable to obtain

for such research. In such situations the research may be done only after consideration and approval of a research ethics committee.

Use of Placebo

33. The benefits, risks, burdens and effectiveness of a new intervention must be tested against those of the best proven intervention(s), except in the following circumstances:

Where no proven intervention exists, the use of placebo, or no intervention, is acceptable; or

Where for compelling and scientifically sound methodological reasons the use of any intervention less effective than the best proven one, the use of placebo, or no intervention is necessary to determine the efficacy or safety of an intervention

and the patients who receive any intervention less effective than the best proven one, placebo, or no intervention will not be subject to additional risks of serious or irreversible harm as a result of not receiving the best proven intervention.

Extreme care must be taken to avoid abuse of this option.

Post-Trial Provisions

34. In advance of a clinical trial, sponsors, researchers and host country governments should make provisions for post-trial access for all participants who still need an intervention identified as beneficial in the trial. This information must also be disclosed to participants during the informed consent process.

Research Registration and Publication and Dissemination of Results

35. Every research study involving human subjects must be registered in a publicly accessible database before recruitment of the first subject.

36. Researchers, authors, sponsors, editors and publishers all have ethical obligations with regard to the publication and dissemination of the results of research. Researchers have a duty to make publicly available the results of their research on human subjects and are accountable for the completeness and accuracy of their reports. All parties should adhere to accepted guidelines for ethical reporting. Negative and inconclusive as well as positive results must be published or otherwise made

publicly available. Sources of funding, institutional affiliations and conflicts of interest must be declared in the publication. Reports of research not in accordance with the principles of this Declaration should not be accepted for publication.

Unproven Interventions in Clinical Practice

37. In the treatment of an individual patient, where proven interventions do not exist or other known interventions have been ineffective, the physician, after seeking expert advice, with informed consent from the patient or a legally authorised representative, may use an unproven intervention if in the physician's judgement it offers hope of saving life, re-establishing health or alleviating suffering. This intervention should subsequently be made the object of research, designed to evaluate its safety and efficacy. In all cases, new information must be recorded and, where appropriate, made publicly available.

Disclaimer: © 2013 World Medical Association, Inc. All Rights Reserved. All intellectual property rights in the Declaration of Helsinki are vested in the World Medical Association. Previous permissions granted to publish or reproduce otherwise WMA policy don't apply to this version of the Declaration of Helsinki until January 1st, 2014. For further questions, please contact the WMA secretariat at secretariat@wma.net.

© World Medical Association, Inc. - All Rights reserved.
© Asociación médica mundial - Todos los derechos reservados.
© L'Association Médicale Mondiale - Tous droits réservés.

Appendix B: Methods and normal values for vital signs, waist and ECG

1. VITAL SIGNS

Systolic and diastolic blood pressure, and heart rate measures are performed by a sphygmomanometer, using the oscillometric method lying after 10 minutes rest (DBP: 50≥N≥90 mm Hg; SBP: 100≥N≥140 mm Hg; HR: 50≤N≤100 bpm).

2. WAIST MEASUREMENT

The Waist measurement must be done at midway between the top of the hip bone and the bottom of the ribs during a normal breathe out

3. ELECTROCARDIOGRAM RECORDINGS

a. ECG expected format

[redacted] minimum recommendations on the paper quality are following ones:

- a 12-lead ECG recording including :
- ECG print out using an A4 format with all ECG leads displayed on a single page

There are many suitable ECG formats, and the two preferred ones are 6x2 and 4x3.

With the 6x2 format, the six standard leads are shown on the left part of the A4 page (lead I down to lead aVF) and the six precordial leads on the right part (Lead V1 down to lead V6).

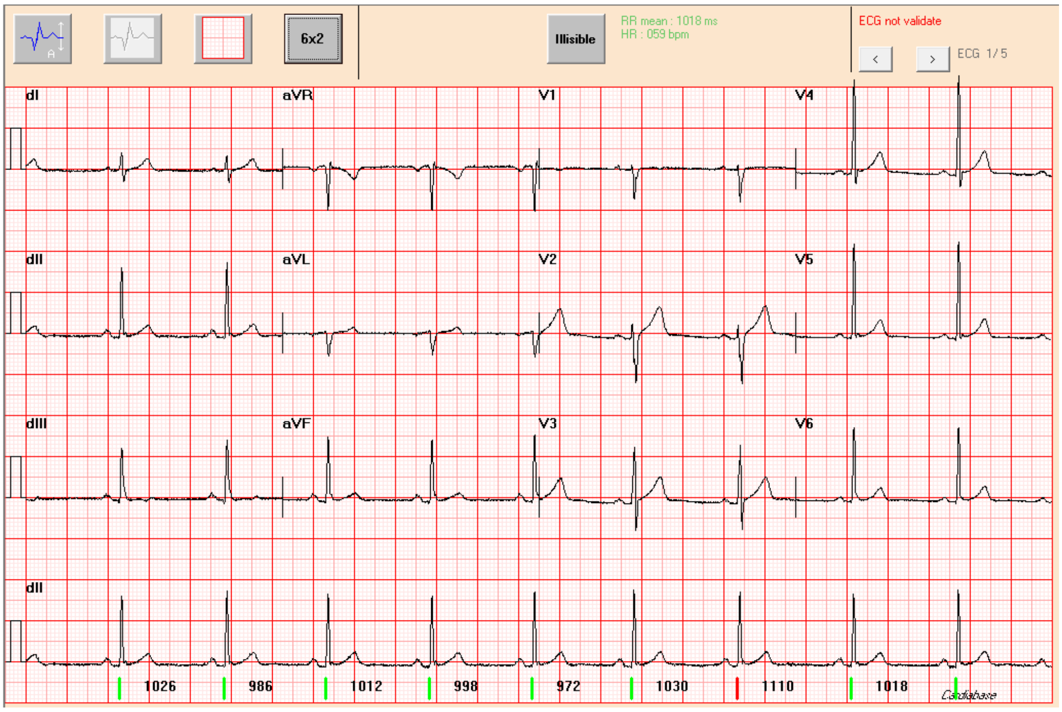

- With the 4x3 format, the order sequence is I/II/III then aVR/aVL/aVF followed by

V1/V2/V3 and V4/V5/V6.

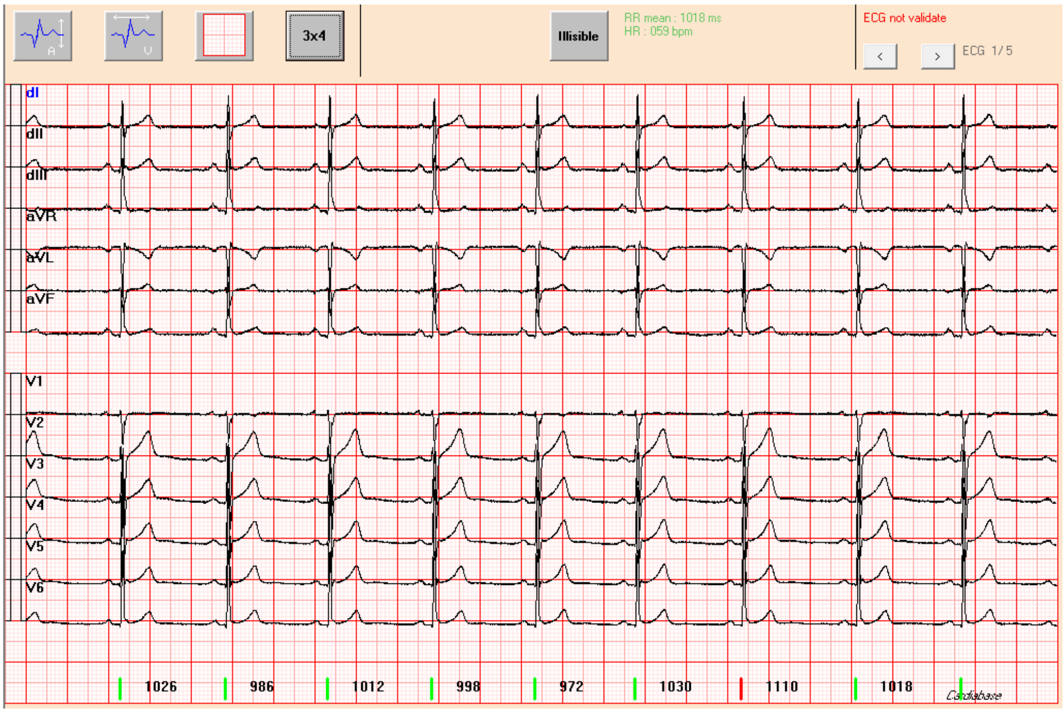

- Regardless of the ECG format, it should include a “rhythm lead” with all individual cardiac beats, typically lead II to allow rhythm statements
- The tracings should clearly show lead ID, the calibration pulse ,the recording speed and finally the gridlines should be clearly visible
- A rhythm strip of 6 to 10 seconds, using the multi-channel (12 leads simultaneously)

Note: should the machine you have on-site not be able to print in A4 format, [redacted] is able to accommodate other formats but the A4 format is the preferred one.

b. ECG Code

For each patient, one ECG per visit is performed.

3 visits are expected for each patient:

- V-1, which is the Baseline ECG
- V1 which is the WEEK4 ECG,
- V3 which is the WEEK24 ECG.

c. Documents provided to Sites by [redacted]

Electronic version (pdf format) of the following documents will be sent:

- User manual for sites
- Transmission form

Other documents, as UPS Airway bills, pre-identified envelopes, black labels and labels customized for the study to identify each ECG print-out, will be sent by courier to each site.

d. Preparation of ECGs for shipment

Review of each ECG will be performed in timeframe of the study.

This ECG review will be performed upon receipt of each ECG after query resolution.

Printouts of ECGs with the corresponding completed transmission form have to be sent to [redacted] by the site just after the recording.

Sites should record ECG in double print-out (i.e. 2 originals, NO PHOTOCOPIES) in order to keep one in patient's medical file and to send the other one to [redacted].

If the ECG device set-up does not allow to double-print the same ECG, and only in this case, the site will print the ECG, copy it. The copy will be kept on site and the original tracing will be sent to [redacted].

The site will send a blinded print-out of each standard ECG with the corresponding transmission form duly completed. All handwritten information or automatic ECG analysis will be hidden with the black labels sent by [redacted].

The paper speed and the ECG date should be kept visible.

A label will be stuck on each ECG page to well identify the study, patient and ECG (visit and date) with paying attention to leave the leads available for analysis.

NATIVE IVA337
Site |__|__|__|__|
PatientNumber:|__|__|__|__|__||__|__|__|
Visit: |__|__|__|
ECG Date : |__|__|/|__|__|__|/|__|__|__|__|__|

APPENDIX C: LIVER BIOPSY AND HISTOLOGY METHODS

1. THE SAF SCORING SYSTEM

The SAF assesses both and separately the grade of steatosis (S), the grade of activity (A), and the stage of fibrosis (F), the latter according to the NASH Clinical Research Network (CRN)(76). Steatosis is assessed by the percentage of hepatocytes containing large and medium-sized intracytoplasmic lipid droplets (but not foamy microvesicles), on a scale of 0 to 3 (S0: <5%; S1: 5%-33%, S2: 34%-66%, S3:>67%). Ballooning of hepatocytes is graded from 0 to 2 (0: normal hepatocytes with cuboidal shape, sharp angles and pink eosinophilic cytoplasm; 1: presence of clusters of hepatocytes with a rounded shape and pale cytoplasm, usually reticulated where, although the shape is different, the size is similar to that of normal hepatocytes; 2, as for grade 1, but where there was also at least one enlarged ballooned hepatocyte (at least 2-fold size compared with that of normal cells within a cluster of hepatocytes with grade 1 ballooning). Lobular inflammation is defined as a focus of two or more inflammatory cells within the lobule organized either as microgranulomas or located within the sinusoids. Foci are counted at 20x magnification (grade 0: none; 1: < 2 foci per lobule; 2: >2 foci per lobule). The grade of activity (A from A0 to A4) is calculated by addition of grades of ballooning and lobular inflammation. Aspects of grading are illustrated Figure 5.

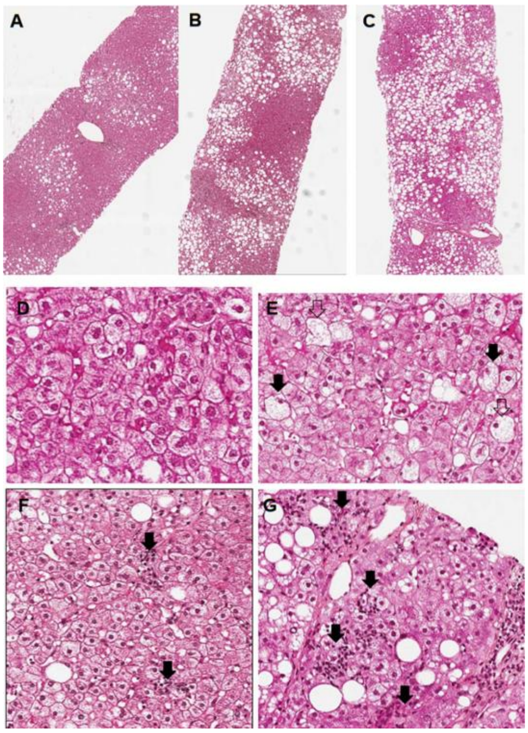

Figure 5: Histological grading used in SAF score. Grading of steatosis.

(A) Grade 1, lipid droplets in 30% of hepatocytes (H&E, x2.5). (B) Grade 2, steatosis in 60% of hepatocytes, (H&E, x2.5). (C) Grade 3: steatosis in >80% of hepatocytes (H&E, x2.5). Hepatocyte ballooning and clarification. (D) Ballooning grade 1: rounded hepatocyte with pale cytoplasm and size quite similar to that of normal hepatocytes (H&E, x20). (E) Ballooning grade 2: presence of enlarged hepatocyte at least 2-fold that of normal cells in a background of clear and rounded hepatocytes, arrow (H&E, x20). Lobular inflammation. (F) Lobular inflammation grade 1: two small foci of inflammatory cells, arrow (H&E, x20).(G) Lobular inflammation grade 2: several large inflammatory foci (arrow) (H&E, x20).

2. LIST OF VARIABLES DERIVED FROM LIVER BIOPSY

1. Biopsy length ______ mm (to be completed)

2. Tissue adequacy for evaluation

Yes ☐ **No** ☐

3. Steatosis

3a Grade (according to NASH CRN):

Grade 0 ☐

Grade 1 ☐

Grade 2 ☐

Grade 3 ☐

3b Location:

Zone 3 (central) ☐

Zone 1 (periportal) ☐

Azonal ☐

Panacinar ☐

4. Hepatocyte Ballooning /clarification (according to SAF Activity score)

Grade 0 ☐

Grade 1 ☐

Grade 2 ☐

5. Lobular inflammation (according to SAF Activity score)

Grade 0 ☐

Grade 1 ☐

Grade 2 ☐

6. Lobular inflammation (according to NASH CRN)

Grade 0 ☐

Grade 1 ☐

Grade 2 ☐

Grade 3 ☐

7. Activity score according to SAF (4+5) **____ (to be completed)**

8. NAS according to NASH CRN (3a+4+6) **____ (to be completed)**

9. Portal inflammation:

None ☐

Mild ☐

More than mild ☐

10. Mallory-Denk bodies:

Absent ☐

Present ☐

11. Fibrosis

11a. Fibrosis stage (According to NASH CRN Scoring for Fibrosis):

Stage 0 ☐

Stage 1a ☐

Stage 1b ☐

Stage 1c ☐

Stage 2 ☐

Stage 3 ☐

Stage 4 ☐

11b. Fibrosis stage (Adapted from Ishak Scoring for Fibrosis):

☐ 0=No fibrosis

☐ 1=Fibrous expansion of portal or central vein area, with or without short fibrous septa

☐ 2=Fibrous expansion of most portal or central areas, with or without short fibrous septa

☐ 3=Fibrous expansion of most portal or central areas, with occasional portal to portal bridging

☐ 4=Fibrous expansion of portal or central areas, with marked bridging

☐ 5=Marked bridging with occasional nodules (incomplete cirrhosis)

☐ 6=Cirrhosis

12. Iron overload

12.a Intensity

Absent ☐

Mild ☐

Moderate ☐

Severe ☐

12.b Location

None ☐

Macrophages ☐

Hepatocytes ☐

Both ☐

13. Diagnosis

Not NAFLD ☐

NAFLD, not NASH ☐

NASH ☐

3. RECOMMENDATIONS FOR SLIDES PREPARATION:

- Five unstained slides must be prepared by the local pathology laboratory for transfer to the central lab
- In case where unstained slides are not available, stained slides could be accepted (H&E plus one slide stained with a staining of fibrous tissue either Masson's trichrome, picrosirius red or reticulin)
- Fixation and paraffin embedding will be performed according to local procedure
- Quality of section is crucial. A sharp knife should be used to recut liver sections on a good microtome by an experienced technician. Optimal technical quality without knife scratches should be obtained.
- Each slide should be cut 4 microns thick and come from the same liver biopsy core whenever possible
- Sections should be mounted preferably on positively charged slides (Superfrost Plus® treated slides)
- Each slide should contain sections 2 cm in length or greater and no less than 15 mm

PREPARATION AND SHIPMENT OF SLIDES TO THE CENTRAL LABORATORY

- Place slides in the plastic slide holder
- Protect the glass slide within the slide holder with adequate material
- Affix an adequate label on the glass slide and on the outside of the slide holder
- Ship the slide holder to the central lab using an Ambient shipper
- Slides will be stained (Hematoxylin & Eosin, Picrosirius red staining, Perls staining) in the central histology laboratory.

PROCEDURE FOR SLIDE READING IN THE DEPARTMENT OF PATHOLOGY

- Shipment will be received on working hours (from Monday to Friday 8:00 AM to 5:00 PM) at the department of the Central reader ([redacted]).
- The day before the shipment, an e-mail will be sent to [redacted] with mention of the number of samples and patient protocol references.
- On reception in the department of pathology, an authorized person will check the number of slides and control that slides are not broken and staining is adequate. In case of problem the central lab will be immediately alerted by returned e-mail.
- In the lab, sections will be transferred to pathologist office for reading. Other slides are stored unstained until immunostaining will be performed by batches.
- After slides reading, the central pathologist will complete a report that will be sent to the central lab to be recorded in the study database.
- The duration between reception of slides and return of eCRF should not exceed 10 working days
- Slides will be stored at [redacted] until the end of the study and then send back to sites. If deemed necessary at the time of the availability of the study results, additional immunohistochemistry analyses could be performed within the year after the end of the study. Until this timepoint, upon Investigator's request, the biopsy slides might be sent back to the site on an ongoing basis.

APPENDIX D : SF-36 QUESTIONNARY

1 **In general, would you say your health is:**

a. Excellent ☐(1)

b. Very good ☐(2)

c. Good ☐(3)

d. Fair ☐(4)

e. Poor ☐(5)

2 **<u>Compared to one year ago</u>, how would you rate your health in general <u>now</u>?**

a. Much better now than one year ago ☐(1)

b. Somewhat better now than one year ago ☐(2)

c. About the same as one year ago ☐(3)

d. Somewhat worse than one year ago ☐(4)

e. Much worse than one year ago ☐(5)

3 **The following items are about activities you might do during a typical day. Does your health now limit you in these activities? If so, how much?**

3.1 Vigorous activities, such as running, lifting heavy objects, participating in strenuous sports

a. Yes, limited a lot ☐(1)
b. Yes, limited a little ☐(2)
c. No, not limited at all ☐(3)

3.2 Moderate activities, such as moving a table, pushing a vacuum cleaner, bowling, or playing golf

a. Yes, limited a lot ☐(1)
b. Yes, limited a little ☐(2)
c. No, not limited at all ☐(3)

3.3 Lifting or carrying groceries

a. Yes, limited a lot ☐(1)
b. Yes, limited a little ☐(2)
c. No, not limited at all ☐(3)

3.4 Climbing several flights of stairs

a. Yes, limited a lot ☐(1)

b. Yes, limited a little ☐(2)
c. No, not limited at all ☐(3)

3.5 Climbing one flight of stairs

a. Yes, limited a lot ☐(1)
b. Yes, limited a little ☐(2)
c. No, not limited at all ☐(3)

3.6 Bending, kneeling or stooping

a. Yes, limited a lot ☐(1)
b. Yes, limited a little ☐(2)
c. No, not limited at all ☐(3)

3.7 Walking more than a mile

a. Yes, limited a lot ☐(1)
b. Yes, limited a little ☐(2)
c. No, not limited at all ☐(3)

3.8 Walking several blocks

a. Yes, limited a lot ☐(1)
b. Yes, limited a little ☐(2)
c. No, not limited at all ☐(3)

3.9 Walking one block

a. Yes, limited a lot ☐(1)
b. Yes, limited a little ☐(2)
c. No, not limited at all ☐(3)

3.10 Bathing or dressing yourself

a. Yes, limited a lot ☐(1)
b. Yes, limited a little ☐(2)
c. No, not limited at all ☐(3)

4 **During the past 4 weeks, have you had any of the following problems with your work or other regular daily activities as a result of your physical health?**

4.1 Cut down on the amount of time you spent on work or other activities

a. Yes☐(1)
b. No ☐(2)

4.2 Accomplished less than you would like

a. Yes ☐(1)
b. No ☐(2)

4.3 Were limited in the kind of work or other activities

a. Yes ☐(1)
b. No ☐(2)

4.4 Had difficulty performing the work or other activities (for example, it took extra effort)

a. Yes ☐(1)
b. No ☐(2)

5 **During the past 4 weeks, have you had any of the following problems with your work or other regular daily activities as a result of any emotional problems (such as feeling depressed or anxious)? (Mark each answer with an X)**

5.1 Cut down the amount of time you spent on work or other activities

a. Yes ☐(1)
b. No ☐(2)

5.2 Accomplished less than you would like

a. Yes ☐(1)
b. No ☐(2)

5.3 Didn't do work or other activities as carefully as usual

a. Yes ☐(1)
b. No ☐(2)

6 **During the past 4 weeks, to what extent has your physical health or emotional problems interfered with your normal social activities with family, friends, neighbors or groups?**

a. Not at all ☐(1)
b. Slightly ☐(2)
c. Moderately ☐(3)
d. Quite a bit ☐(4)
e. Extremely ☐(5)

7 **How much bodily pain have you had during the past 4 weeks?**

a. None ☐(1)
b. Very mild ☐(2)
c. Mild ☐(3)
d. Moderate ☐(4)
e. Severe ☐(5)
f. Very severe ☐(6)

8 **During the past 4 weeks, how much did pain interfere with your normal work**

(including both work outside the home and housework) ?

a. Not at all ☐(1)
b. A little bit ☐(2)
c. Moderately ☐(3)
d. Quite a bit ☐(4)
e. Extremely ☐(5)

9 **These questions are about how you feel and how things have been with you during the past 4 weeks. For each question, please give the one answer that comes closest to the way you have been feeling. How much of the time during the past 4 weeks – (Mark each answer with an X)**

	All of the Time (1)	Most of the Time (2)	A Good Bit SF- of the Time (3)	Some of the Time (4)	A Little of the Time (5)	None of the Time (6)
**9.1 Did you feel full of pep?**						
**9.2 Have you been a very nervous person?**						
**9.3 Have you felt so down in the dumps that nothing could cheer you up?**						
**9.4 Have you felt calm and peaceful?**						
**9.5 Did you have a lot of energy?**						
**9.6 Have you felt downhearted and blue?**						
**9.7 Did you feel worn out?**						
**9.8 Have you been a happy person?**						
**9.9 Did you feel tired?**						

10 **During the past 4 weeks, how much of the time has your physical health or emotional problems interfered with your social activities (like visiting with friends, relatives, etc.)?**

f. All of the time ☐(1)
g. Most of the time ☐(2)
h. Some of the time ☐(3)
i. A little of the time ☐(4)
j. None of the time ☐(5)

11 **How TRUE or FALSE is each of the following statements for you?**

	Definitely True (1)	Mostly True (2)	Don't Know (3)	Mostly False (4)	Definitely False (5)
**11.1 I seem to get sick a little easier than other people**					
**11.2 I am as healthy as anybody I know**					
**11.3 I expect my health to get worse**					
**11.4 My health is excellent**					

APPENDIX E : THE FLINDERS FATIGUE SCALE (FFS)

FATIGUE SCALE

Name______________________________ Date____________________

We are interested in the extent that you have felt **fatigued** (tired, weary, exhausted) over the last **two weeks**. We **do not** mean feelings of **sleepiness** (the likelihood of falling asleep). Please circle the appropriate response in accordance with your average feelings over this two-week period.

1 **Was fatigue a problem for you?**

0	1	2	3	4
Not at all		Moderately		Entirely

2 **Did fatigue cause problems with your everyday functioning (e.g., work, social, family)?**

0	1	2	3	4
Not at all		Moderately		Entirely

3 **Did fatigue cause you distress?**

0	1	2	3	4
Not at all		Moderately		Entirely

4 **How often did you suffer from fatigue?**

0	1	2	3	4
0 days/week	1-2 days/week	3-4 days/week	5-6 days/week	7 days/week

5 **At what time(s) of the day did you typically experience fatigue? (Please tick box(es))**

Early morning	☐	Late afternoon	☐
Mid morning	☐	Early evening	☐
Midday	☐	Late evening	☐
Mid afternoon	☐		

6 **How severe was the fatigue you experienced?**

0	1	2	3	4
Not at all		Moderately		Entirely

7 **How much was your fatigue caused by poor sleep?**

0	1	2	3	4
Not at all		Moderately		Entirely

SAS® code/procedures

Mean ± standard deviation and 95% confidence interval

```
****************************************;
* Used in Figure 1, Figure 2C, Figure 3, Figure 4, Supplementary Table 2, Table 4,
Table 5, Table 6, Table 7, Table 8, Table 12 and Figure 5;
****************************************;
proc means data=[dataset];
     by [group] [visit];
     var [parameter];
     output out=[results dataset] n=n mean=mean lclm=lclm uclm=uclm std=std min=min
     max=max q1=q1 median=median q3=q3;
run;
```

Frequency and 95%confidence interval

```
****************************************;
* Used in Figure 2A, Figure 2D, Figure 5, Supplementary Table 2, Table 3, Table 4,
Table 6, Table 7, Table 10, Figure 1 and Figure 3;
****************************************;
proc freq data=[dataset];
     exact Binomial;
     * if the modality is not present in the dataset => IC modality = 1-IC of all
     other values;
     tables var / CL Alpha=0.05 exact Binomial (all
          %if %sysfunc(exist(_val_exist_)) ne 0 %then %do;
               level="ok" P=0.5
          %end;
     );
     output out=[results dataset] BINOMIAL;
run;
```

Student test, ANOVA, Wilcoxon-Mann-Whitney test and Kruskall-Wallis test

```
****************************************;
* Used in Figure 2C, Supplementary Table 4, Table 8 and Table 12;
****************************************;
proc npar1way data=[dataset] edf wilcoxon anova;
     class [group];
     var [parameter];
     output out=[results dataset];
run;
```

Chi² test and Fisher test

```
****************************************;
* Used in Figure 2D, Supplementary Table 3 and Table 10;
****************************************;
proc freq data=[dataset];
     tables *[group]*[parameter] / chisq exact;
     exact fisher / MAXTIME=10;
     output out=[results dataset] nmiss n chisq exact;
run;
```

Mixed Model for Repeated Measures

```
******************************************;
* Used in Table 1, Figure 1, Supplementary Table 13 and Figure 5;
* with:
*    &nb chg vis.: the number of post-baseline visits;
*    &mvExplQLvar.: any other explicative parameter(s) (e.g. diabetic status);
******************************************;
proc mixed data=[dataset];
     class [subject identifier] [visit] [group] &mvExplQLvar.;
     model [change from baseline] = [visit] [group] [group]*[visit] &mvExplQLvar.
     [baseline value] / ddfm=kr residual;
     * if only one visit then no repeated;
     %if &nb chg vis. ne 1 %then %do;
          repeated [visit] / subject=[subject identifier] type=UN;
     %end;
     lsmeans [group]*[visit] / pdiff cl;
     ods output LSMeans=LSMeans Diffs=Diffs;
run;
```

Cochran-Mantel-Haenszel test

```
******************************************;
* Used in Figure 2A
* with:
*    "pink”: the group modalities to be compared;
******************************************;
proc freq data=[dataset];
     where [group] in ("modality to be compared to" "modality to compare");
     tables [diabetic status]*[group]*[parameter] / CMH CHISQ;
     output out=[results dataset] CMH CHISQ;
run;
```

Spearman correlation

```
******************************************;
* Used in Figure 2B, Supplementary Table 9, Table 11, Figure 2, Figure 4
******************************************;
proc corr data=[dataset] spearman fisher (biasadj=no);
     id [subject identifier];
     var [parameter Y] [parameter X];
     ods output SpearmanCorr=CorrS FisherSpearmanCorr=CorrS_CI;
run;
```

BoxPlot

```
******************************************;
* Used in Figure 2B, Supplementary Figure 2 and Figure 4
* with:
*    &GRP_ORDER: the [group] modalities in ascending order
******************************************;
Proc sgplot data=[dataset]
     vbox [parameter] / group=[group] nofill; * Figure 2B and Supplementary Figure 4
     or
     vbox [parameter] / group=[group] category=[group] nooutliers nofill; *
     Supplementary Figure 2

     xaxis values=(&GRP_ORDER.);
run;
```
