# Supplementary material for: The pan-PPAR agonist lanifibranor improves cardiometabolic health in patients with metabolic dysfunction-associated steatohepatitis
Source: Nat Commun. 2024 May 10;15:3962. doi: 10.1038/s41467-024-47919-9 (PMC11087475; doi:10.1038/s41467-024-47919-9)
Supplement: Supplementary file 3 — Reporting Summary [file 41467_2024_47919_MOESM3_ESM.pdf]

nature portfolio

Corresponding author(s): Michael P. Coorman

Last updated by author(s): Mar 26, 2024

Reporting Summary

Nature Portfolio wishes to improve the reproducibility of the work that we publish. This form provides structure for consistency and transparency in reporting. For further information on Nature Portfolio policies, see our Editorial Policies and the Editorial Policy Checklist.

Statistics

For all statistical analyses, confirm that the following items are present in the figure legend, table legend, main text, or Methods section.

n/a	Confirmed	
☐	☒	The exact sample size (*n*) for each experimental group/condition, given as a discrete number and unit of measurement
☐	☒	A statement on whether measurements were taken from distinct samples or whether the same sample was measured repeatedly
☐	☒	The statistical test(s) used AND whether they are one- or two-sided<br>*Only common tests should be described solely by name; describe more complex techniques in the Methods section.*
☐	☒	A description of all covariates tested
☐	☒	A description of any assumptions or corrections, such as tests of normality and adjustment for multiple comparisons
☐	☒	A full description of the statistical parameters including central tendency (e.g. means) or other basic estimates (e.g. regression coefficient) AND variation (e.g. standard deviation) or associated estimates of uncertainty (e.g. confidence intervals)
☐	☒	For null hypothesis testing, the test statistic (e.g. *F*, *t*, *r*) with confidence intervals, effect sizes, degrees of freedom and *P* value noted<br>*Give P values as exact values whenever suitable.*
☒	☐	For Bayesian analysis, information on the choice of priors and Markov chain Monte Carlo settings
☒	☐	For hierarchical and complex designs, identification of the appropriate level for tests and full reporting of outcomes
☐	☒	Estimates of effect sizes (e.g. Cohen's *d*, Pearson's *r*), indicating how they were calculated

Our web collection on statistics for biologists contains articles on many of the points above.

Software and code

Policy information about availability of computer code

Data collection	Clinical data were collected using eCRF, external laboratory data were quantified at the central lab CERBA (former BARC).
Data analysis	All statistics were calculated using the SAS software, version 9.4. Descriptive statistics were presented using the mean and standard deviation, median, interquartile ranges and minimum-maximum for continous variables, and frequency counts and percentages for categorical variables. For statistical tests, the type-I error risk was set at 5% (2-sided) as per protocol. No multiplicity adjustments were performed for the analyses presented in this paper, that are considered exploratory. Point estimates, 95% confidence intervals (CI) and p-values are provided. The CIs have not been adjusted for multiple comparisons. Comparisons of continuous parameters between treatment groups at EOT were done using Mixed Model for Repeated Measures (MMRM), using change from baseline as endpoint, the time (Weeks 4, 12 and 24), treatment, the diabetic status, the interaction (treatment*time) and the baseline value as fixed effects, a time repeated measure within each subject and an unstructured variance covariance matrix, or using Student or Wilcoxon tests depending on tests of normality. Comparison of categorical parameters between groups was done using Cochran–Mantel–Haenszel test stratified on the diabetic status at Baseline, or from Chi^2 or Fisher tests. The correlation between continuous parameters was estimated using Spearman correlation (coefficient [Rs] and p-value). The SAS® codes that support the findings of this study are available from the corresponding author upon reasonable request.

For manuscripts utilizing custom algorithms or software that are central to the research but not yet described in published literature, software must be made available to editors and reviewers. We strongly encourage code deposition in a community repository (e.g. GitHub). See the Nature Portfolio guidelines for submitting code & software for further information.

Data

Policy information about availability of data

All manuscripts must include a data availability statement. This statement should provide the following information, where applicable:

- Accession codes, unique identifiers, or web links for publicly available datasets
- A description of any restrictions on data availability
- For clinical datasets or third party data, please ensure that the statement adheres to our policy

The data that support the findings of this study are available from the corresponding author upon reasonable request. Proposals should be directed to Michael.COOREMAN@inventivapharma.com. All requests for data will be reviewed by the corresponding author, who will make sure that the requested data are available, consistent with participant privacy and informed consent. A response will be provided within three months. Data requestors will need to sign a data access agreement. Source data have been provided with this paper and its supplementary information files.

Research involving human participants, their data, or biological material

Policy information about studies with human participants or human data. See also policy information about sex, gender (identity/presentation), and sexual orientation and race, ethnicity and racism.

Reporting on sex and gender	Sex was collected in the eCRF by investigators.<br>No a priori sex- or gender-based analyses were defined or performed for this study and no sex- or gender-based analyses are provided in the paper. For the analyses that are reported in this paper, a post-hoc based sex-based analysis showed that a sex-based difference was only observed for weight change. There were no other sex-based differences, and this difference observed for weight change had no effect on the analyses presented in the paper, including the correlation between weight gain and cardiometabolic health. As this sex-based information does not contribute to message and conclusion on this topic, i.e. that with lanifibranor therapy improvement of cardiometabolic health is not related to weight change.
Reporting on race, ethnicity, or other socially relevant groupings	Race was collected in the eCRF by Investigators but was not used in the manuscript.
Population characteristics	Subjects of ≥18 years old with MASH confirmed by a centrally read biopsy performed at screening or in the preceding six months and a histological Steatosis Activity Fibrosis score for disease activity (inflammation and ballooning) ≥3.<br>Patients with cirrhosis, hemoglobin A1c >8.5% (69 mmol/mol), recent change in anti-diabetic medication, type-1 diabetes or T2D on insulin therapy and other causes of chronic liver disease, including significant daily alcohol consumption, were excluded.
Recruitment	Patients were invited to participate in the study by their refering doctor according to the patient's medical records. Patients had to fulfil all the inclusion and none of the exclusion criteria to be eligible.
Ethics oversight	This clinical trial has been registered at ClinicalTrials.gov, number NCT03008070 (first posted January 2, 2017). This study and all amendments have been approved by independent ethics committees and the appropriate authorities (Supplementary Table 1) in 16 countries (France, United States of America, Australia, Belgium, Bulgaria, Germany, Canada, Italy, Spain, Poland, the United Kingdom, Czech Republic, Switzerland, Slovenia, Austria and Mauritius), where at least one patient underwent randomization. The trial was conducted in accordance with the principles of the Declaration of Helsinki, the International Council for Harmonisation Good Clinical Practice guidelines, and all relevant regulations. Prior to the trial entry, written informed consent was obtained from all participants.

Note that full information on the approval of the study protocol must also be provided in the manuscript.

Field-specific reporting

Please select the one below that is the best fit for your research. If you are not sure, read the appropriate sections before making your selection.

[x] Life sciences [ ] Behavioural & social sciences [ ] Ecological, evolutionary & environmental sciences

For a reference copy of the document with all sections, see nature.com/documents/nr-reporting-summary-flat.pdf

Life sciences study design

All studies must disclose on these points even when the disclosure is negative.

Sample size	The expected rate of responders was estimated to 10% for the placebo by a group of clinical experts. An excess rate of responders of 20% was accepted as clinically pertinent. The sample size required to reach a power of at least 80% was 72 patients per group with a two-sided alpha of 0.025 (adjustment for multiplicity). The sample size is sensitive to the rate of responders in the placebo group: the sample size was therefore rounded up on the conservative side to 75 patients per group.<br>Finally 247 patients were actually randomised, mainly due to the multicentric nature of the study.
Data exclusions	No data were excluded from the analyses.
Replication	All data were monitored and quality controlled. At least 2 statisticians verified the reproducibility of the experimental findings. All

	attempts at replication were successful.
Randomization	Patients were randomized 1:1:1 to receive lanifibranor 1200 mg, 800 mg, or placebo, once daily for 24 weeks, and the randomization was stratified on type 2 diabetes (presence/absence).
Blinding	NATIVE was a double-blind trial therefore investigators/participants were blinded to group allocation during data collection and analysis.

Reporting for specific materials, systems and methods

We require information from authors about some types of materials, experimental systems and methods used in many studies. Here, indicate whether each material, system or method listed is relevant to your study. If you are not sure if a list item applies to your research, read the appropriate section before selecting a response.

Materials & experimental systems

n/a	Involved in the study
☒	☐ Antibodies
☒	☐ Eukaryotic cell lines
☒	☐ Palaeontology and archaeology
☒	☐ Animals and other organisms
☐	☒ Clinical data
☒	☐ Dual use research of concern
☒	☐ Plants

Methods

n/a	Involved in the study
☒	☐ ChIP-seq
☒	☐ Flow cytometry
☒	☐ MRI-based neuroimaging

Clinical data

Policy information about clinical studies

All manuscripts should comply with the ICMJEguidelines for publication of clinical research and a completedCONSORT checklist must be included with all submissions.

Clinical trial registration	ClinicalTrials.gov number: NCT03008070
Study protocol	Trial protocol is described at https://doi.org/10.1016/j.cct.2020.106170, Contemporary Clinical Trials, Volume 98, November 2020, A randomised, double-blind, placebo-controlled, multi-centre, dose-range, proof-of-concept, 24-week treatment study of lanifibranor in adult subjects with non-alcoholic steatohepatitis: Design of the NATIVE study, Francque Sven M & All
Data collection	This multicenter, parallel-group, randomised, double-blind, 24-week treatment, placebo-controlled Phase 2 NATIVE trial was conducted in 16 countries (France, United States of America, Australia, Belgium, Bulgaria, Germany, Canada, Italy, Spain, Poland, the United Kingdom, Czech Republic, Switzerland, Slovenia, Austria and Mauritius), from 07Feb2017 (first patient first visit) to 16Mar2020 (last patient last visit). A total of 247 patients were enrolled and randomly assigned to one of the 3 treatment groups. Study visits took place at Screening, Baseline, Week 4, Week 14 and Week 24 and at follow-up visit 4 weeks after trial drug termination (Week 28). Efficacy and Safety assessments were performed at each visit.
Outcomes	For the assessment of the primary endpoint, on an ongoing basis, a single pathologist reviewed the pre and post-treatment biopsies, blinded for timing (screening vs. end of treatment) and treatment allocation. He completed a list of variables derived from liver biopsy for each patient/biopsy. The collected variables included scoring of steatosis, hepatocyte ballooning, lobular and portal inflammation, NAFLD activity score (NAS) and fibrosis stage according to SAF (Steatosis-Activity-Fibrosis) and CRN scoring systems. The SAF-Activity score combined hepatocellular inflammatory and ballooning, and was derived as the sum of SAF-Inflammation and SAF-Ballooning.<br>The primary efficacy outcome was a binary variable (responder / non responder) based on the change from baseline to Week 24 of the SAF-Activity score. Responder was defined as a decrease from baseline to week 24 of at least 2 points of the SAF activity score without worsening of fibrosis.<br>Main secondary outcomes included histological outcomes (NASH resolution without worsening of fibrosis, Improvement of Fibrosis without worsening of NASH, Change in components of SAF-Steatosis, Lobular inflammation and Ballooning, Change in components of CRN-Steatosis, Lobular inflammation, Ballooning and Fibrosis), changes from baseline to 24 weeks of treatment in Liver enzymes, Inflammatory markers, Glucose metabolism, plasma lipids levels and Adiponectin. Safety was assessed using Adverse Events, vital signs, electrocardiogram and laboratory parameters.

Plants

Seed stocks	Not applicable to the study, such data were not collected.
Novel plant genotypes	Not applicable to the study, such data were not collected.
Authentication	Not applicable to the study, such data were not collected.
